# Visible-Light-Induced Singlet Oxygen-Promoted Arylation and Alkylation of Quinoxalin-2(1H)-ones and Quinolines

**DOI:** 10.3390/molecules29215113

**Published:** 2024-10-29

**Authors:** Renjun Tan, Hequn Yang, Min Jiang, Peijun Song

**Affiliations:** 1 School of Science, Wuhan University of Technology, Wuhan 430070, China; 2 Key Laboratory of Organosilicon Chemistry and Material Technology, Ministry of Education, Key Laboratory of Organosilicon Material Technology, College of Material Chemistry and Chemical Engineering, Hangzhou Normal University, Hangzhou 311121, China; yanqun1419@163.com (R.T.);

**Keywords:** metal-free, ESR, singlet oxygen, visible-light-induced, arylation and alkylation

## Abstract

We report a green and efficient visible-light-driven method for the arylation and alkylation of quinoxalin-2(1H)-ones and quinolines. This catalyst-free process utilizes air as the oxidant, offering mild reaction conditions, environmental sustainability, and broad functional group compatibility. The approach enables the synthesis of aryl and alkyl derivatives of quinoxalin-2(1H)-ones and quinolines with high to excellent yields.

## 1. Introduction

Nitrogen-containing aromatic heterocycles are important structural motifs found in a wide range of pharmaceutical drugs and bioactive compounds [1,2,3]. Among these, 3-substituted quinoxaline-2(1H)-ones and quinolone derivatives have notable applications in synthetic chemistry, materials science, natural products, and pharmaceuticals due to their strong biological activities and excellent chemical properties [1,2,3,4,5,6,7]. In recent years, several methods for the direct C3 arylation [8,9,10,11,12,13,14,15,16,17] and alkylation [18,19,20,21,22,23] of quinoxaline-2(1H)-ones have been developed. However, many of these approaches rely on transition metals, toxic organic solvents, or high reaction temperatures, falling short of green chemistry standards [24,25,26,27,28,29,30,31,32,33]. As a result, there is ongoing interest in developing sustainable and environmentally friendly methods for C3 arylation and alkylation. Air, being a cost-effective, abundant, and safe oxidant, aligns perfectly with green chemistry principles. Over the past decade, photocatalytic reactions using molecular oxygen (or air) [10,34,35,36,37] as a terminal oxidant have gained prominence in green chemistry [31,34,35,36,37,38,39,40,41,42]. Photocatalysis, with its environmentally benign nature, mild conditions, and functional group tolerance, is increasingly viewed as an ideal green chemistry approach, in line with the 12 principles of green chemistry [27,28,43,44,45].

## 2. Results

### 2.1. Electron Paramagnetic Resonance Studies of Reaction Intermediates

**Figure 1:**
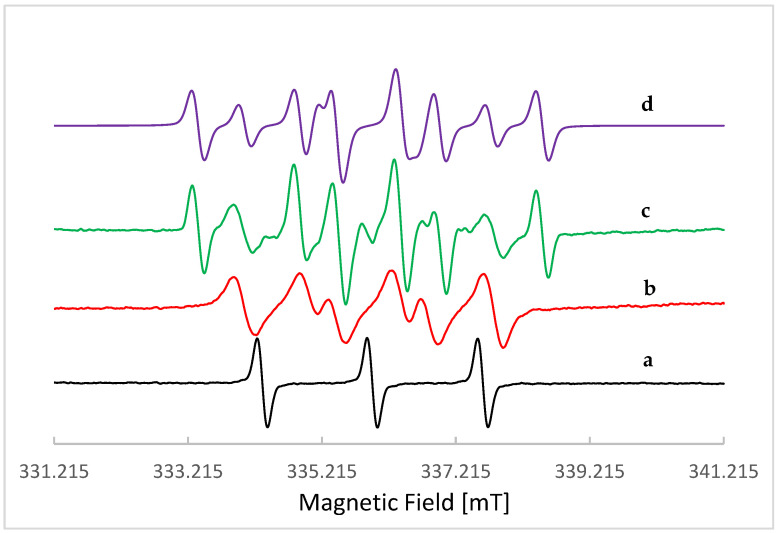
ESR spectrum of the intermediates produced from (**a**) 1-methylquinoxalin-2(1H)-one (20 mM) and TEMP (30 mM) in CH_3_CN, irradiated with 400 nm LEDs for 5 min; (**b**) mixture of 1-methylquinoxalin-2(1H)-one (20 mM), phenylhydrazine (20 mM), and DMPO (50 mM) in CH_3_CN, irradiated with 400 nm LEDs for 20 s; (**c**) mixture of 1-methylquinoxalin-2(1H)-one (20 mM), phenylhydrazine (20 mM), and DMPO (50 mM) in CH_3_CN, irradiated with 400 nm LEDs for 5 min; (**d**) simulated spectrum of spectrum (**c**).

To address these challenges, we developed a more environmentally friendly and efficient synthesis approach. It has been reported that singlet oxygen (^1^O_2_) [10,44,46,47] can be easily generated from air or oxygen in the presence of quinoxaline-2(1H)-ones or their derivatives under irradiation with suitable wavelength light. Singlet oxygen, a reactive oxygen species can oxidize organic compounds to form organic radicals, which subsequently react with quinoxaline-2(1H)-ones to produce C3 radical addition products. In our study, organic hydrazines, which are stable and readily available radical sources, were employed, and their involvement was confirmed through electron spin resonance (ESR) experiments (Figure 1).

A strong singlet oxygen signal with g = 2.0034 and AN = 1.56 mT was detected when a mixture of 1-methylquinoxalin-2(1H)-one (20 mM) and TEMP (2,2,6,6-tetramethylpiperidin) (30 mM) in CH_3_CN was irradiated with visible light (400 nm) for 5 min (Figure 1a). In a separate experiment, a mixture of 1-methylquinoxalin-2(1H)-one (20 mM), phenylhydrazine (20 mM), and DMPO (50 mM) in CH_3_CN, when irradiated for 20 s with visible light (400 nm), produced a superoxide anion radical with g = 2.0034, AN = 1.15 mT, and AH = 0.96 mT (Figure 1b). Upon extending the irradiation time to 5 min, a sextet signal appeared alongside the superoxide anion radical, characterized by g = 2.0026, AN = 1.51 mT, and AH = 2.15 mT, which is indicative of a carbon-centered radical formed through the oxidation of phenylhydrazine by singlet oxygen (Figure 1c).

### 2.2. Reaction Condition Optimization C-3 Arylation of 1-Methylquinoxalin-2(1H)-One

Based on the ESR results, we investigated the feasibility of visible-light-induced C3 arylation and alkylation using 1-methylquinoxalin-2(1H)-one (**1a**) and phenylhydrazine (**2a**) as model substrates. As shown in Table 1, the optimal conditions were achieved with MeCN as the solvent under purple LED (400 nm) irradiation for 12 h in air (entry 2). Various solvents were tested, including acetone, DMSO, EtOH, and dioxane, which significantly reduced the reaction efficiency (entries 3–14). Water, considered a green solvent [27,40,48,49,50], produced only a 30% yield (entry 11). Mixed solvents were also examined, but they did not improve the reaction outcome (entries 12–14). Increasing the amount of 2a to 2–3 equivalents led to a decline in yield (entries 15–17), and extending the reaction time also resulted in lower yields (entries 18–19). Control experiments confirmed that light irradiation is essential for optimal performance (entry 22). Additionally, conducting the reaction under nitrogen, air, or oxygen atmospheres resulted in yields of 0%, 93%, and 95%, respectively (entries 21, 23), suggesting that oxygen plays a critical role in the photocatalytic cycle.

### 2.3. Substrate Expansion

**Scheme 1:**
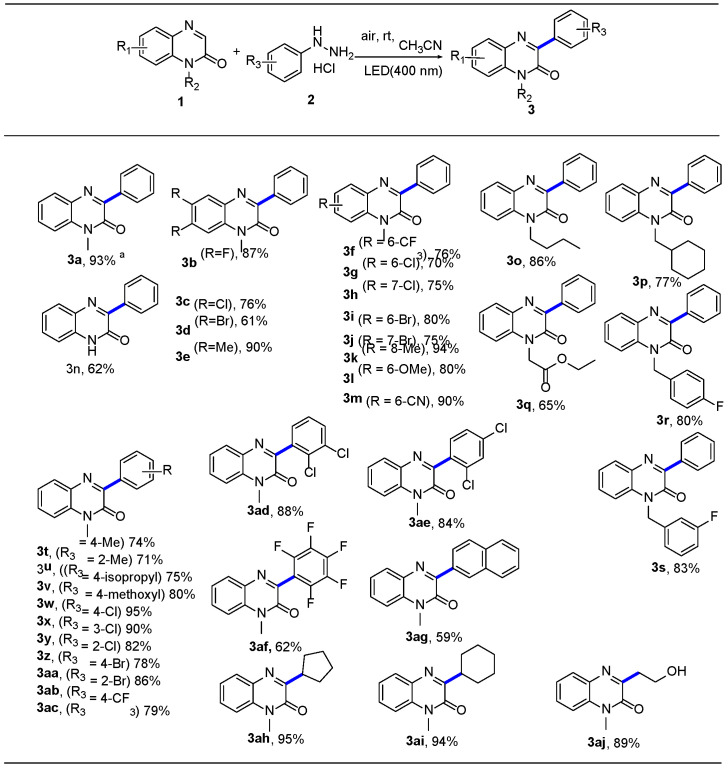
Scope of quinoxalin-2(1H)-ones. Reaction conditions: **1** (0.2 mmol), **2** (0.2 mmol), in 2 mL CH_3_CN at room temperature, 12 h, isolated yields. ^a^ Reaction was performed in 1.0 mmol.

With the optimized conditions for visible-light-induced C3 arylation, we extended this heterogeneous photocatalytic system to various substituted quinoxalin-2(1H)-ones and arylhydrazine hydrochlorides. As shown in Scheme 1, N-methylated quinoxalin-2(1H)-ones with halogen, electron-donating, or electron-withdrawing groups (such as methyl, methoxy, and trifluoromethyl) on the benzene ring (**3b**–**3m**) produced the desired C3 arylated products in moderate to good yields. In addition, N-free, N-alkylated, and N-benzylated quinoxalin-2(1H)-ones also yielded the target products in satisfactory amounts (**3n**–**3r**). A variety of arylhydrazine hydrochlorides, particularly those with halogen substituents at different positions on the benzene ring, provided good yields of C3 arylated quinoxalin-2(1H)-ones (**3x**–**3ab**, **3ad**–**3af**) (Scheme 1). Experiments further demonstrated that arylhydrazines with electron-donating or electron-withdrawing groups (e.g., alkyl, methoxy, trifluoromethyl) on the ortho or para positions of the benzene ring also gave high yields of C3-arylated products (**3t**–**3w**, **3ac**, **3ag**). We then applied the system to visible-light-induced alkylation of quinoxalin-2(1H)-ones, which successfully yielded C3-alkylquinoxalin-2(1H)-ones (**3ah**–**3aj**). To the best of our knowledge, this is the first reported reaction of alkyl hydrazine with quinoxalin-2(1H)-ones under metal-, base-, oxidant-, and catalyst-free conditions.

**Scheme 2:**
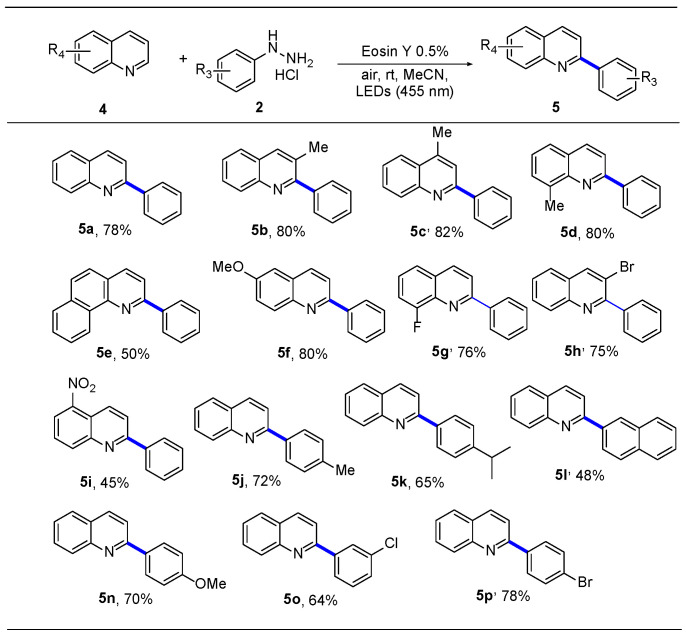
Scope of quinoxalin-2(1H)-ones. Reaction conditions: **4** (0.2 mmol), **2** (0.2 mmol), in 2 mL CH_3_CN at room temperature, 12 h, isolated yields. Reaction was performed in 1.0 mmol.

To showcase the practical utility of this method, a gram-scale synthesis of C-3-arylated quinoxalin-2(1H)-ones (**3a**) was carried out, yielding the product in 91% yield (Scheme 2). Building on the ESR results, we further applied the reaction to the arylation of quinolines. Using Eosin Y (0.5%), which was a green and easily available photocatalyst to generate singlet oxygen from oxygen as a photosensitizer to activate oxygen into singlet oxygen, the arylation of quinolines was successfully achieved with moderate to good (Scheme 2). These findings suggest that this method holds broad potential for the arylation of nitrogen-containing aromatic heterocycles.

### 2.4. Mechanism Study

**Scheme 3:**
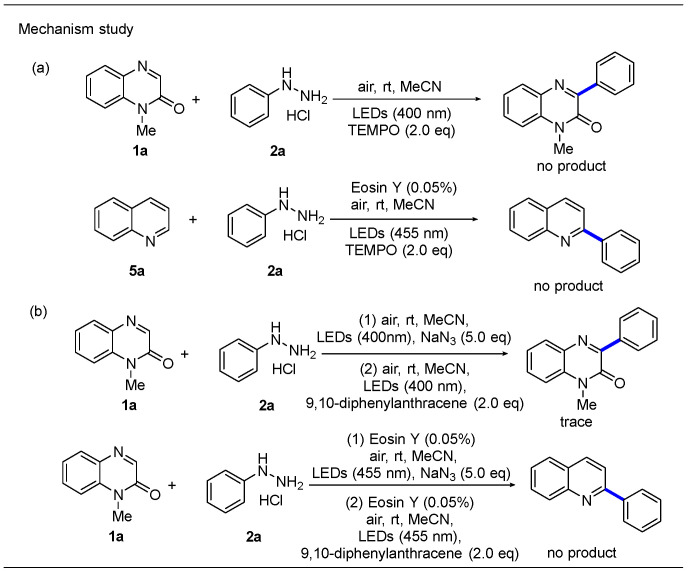
(**a**) TEMPO trapping experiment; (**b**) Singletoxygen quenching experiments with NaN_3_ and 9,10-diphenylanthracene.

**Figure 2:**
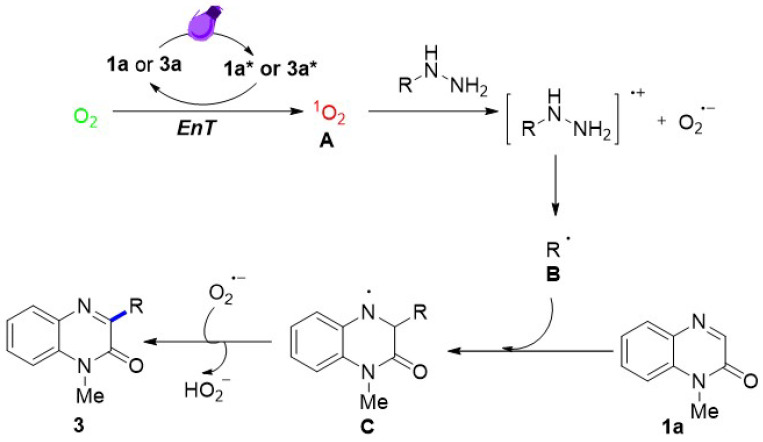
Plausible mechanism of metal-free visible-light-induced arylation and alkylation of nitrogen-containing aromatic heterocycles.

TEMPO (2.0 equivalent) was added to the model reaction to further verify the mechanism of the reaction; no product was obtained. This further confirmed the mechanism as a radical reaction process. The same phenomenon occurred when singlet oxygen quencher NaN_3_ (5.0 equivalent) and 9,10-diphenylanthracene (2.0 equivalent) were added to the model reaction (Scheme 3). Combining the previous reports [10,18,39,40,51,52,53,54,55,56] with our ESR experiments (Figures S1–S3), a plausible mechanism was proposed. Quinoxaline-2(1H)-one (**1a**) or the arylation/alkylation products (**3a**) was activated by a purple LED (400 nm) to generate a triplet 1a* or 3a*, then the triplet 1a* or 3a* activated oxygen to singlet oxygen A via an energy transfer, the high-oxidizing singlet oxygen oxidized organic hydrazines into corresponding carbon-centered radicals B and superoxide oxygen anionic radical, B then attacked 1a to give the intermediate C, after C was oxidized by superoxide oxygen anionic radical to produce 3 (Figure 2).

## 3. Discussion

In this study, we developed a green and efficient visible-light-induced method for the C3 arylation and alkylation of nitrogen-containing aromatic heterocycles with a specific focus on quinoxaline-2(1H)-ones. The use of air as the oxidant and the absence of photocatalysts highlight the environmentally friendly nature of this protocol. Our optimized reaction conditions—utilizing MeCN as a solvent and visible light (400 nm) irradiation—proved effective, with the target products obtained in moderate to excellent yields.

The ESR studies provided crucial mechanistic insights into the reaction. Singlet oxygen, generated from the air in the presence of light and quinoxaline-2(1H)-ones, plays a pivotal role in oxidizing organic compounds to produce the necessary radicals. These radicals then undergo C3 radical addition, resulting in the desired products. The use of organic hydrazines as stable radical sources further streamlined the reaction, confirming their role through ESR spectroscopy.

Extending this methodology, we applied the optimized conditions to a variety of substituted quinoxaline-2(1H)-ones and arylhydrazine hydrochlorides. The results demonstrated broad substrate scope and functional group tolerance. N-methylated quinoxaline-2(1H)-ones bearing halogens, electron-donating, and electron-withdrawing groups yielded the desired arylated products in moderate to good yields. Moreover, N-free, N-alkylated, and N-benzylated quinoxaline-2(1H)-ones also showed satisfactory results. The successful application to a wide range of arylhydrazine hydrochlorides, including those with halogen groups, further validated the versatility of the approach.

We also explored the alkylation of quinoxaline-2(1H)-ones under visible light, achieving the C-3-alkylquinoxaline products in good yields. Notably, this is the first reported instance of alkyl hydrazine and quinoxaline-2(1H)-ones reacting under photocatalyst- and oxidant-free conditions, which positions this method as a significant advance in the field of green chemistry.

The gram-scale synthesis of C-3-arylated quinoxaline-2(1H)-ones showcased the scalability of the method, achieving a high yield of 91%. Furthermore, extending the reaction to the arylation of quinolines using Eosin Y as a photocatalyst highlights the broader applicability of this strategy. The efficient arylation of quinolines in moderate to good yields underscores the potential of this method for the functionalization of other nitrogen-containing heterocycles.

In summary, this work offers a sustainable, metal-free, and mild approach for the arylation and alkylation of quinoxaline-2(1H)-ones and quinolones. The use of singlet oxygen as the reactive species, generated under visible light, exemplifies the alignment of this method with green chemistry principles. Future work could explore further expansion of the substrate scope and detailed mechanistic studies to enhance the understanding of these photocatalytic transformations.

## 4. Materials and Methods

C3-arylated/alkylated of quinoxalin-2(1H)-ones: To a 5 mL vial was added substituted quinoxalin-2(1H)-ones 1 (0.2 mmol, 1.0 equivalent) and arylhydrazines or arylhydrazines hydrochloride/alkylhydrazines or alkylhydrazines hydrochloride 2 (0.2 mmol, 1.0 equivalent), subsequently, MeCN (2 mL) was added. The reaction mixture was stirred under an air irradiated by purple LED (λmax = 400 nm) from a 3.0 cm distance for 12 h at room temperature. The mixture was evaporated in a vacuum, then it was purified by silica gel column chromatography with an elution of PE-EA to afford the desired product.

Arylation of quinolines: To a 5 mL vial was added substituted quinolines 4 (0.2 mmol, 1.0 equivalent) and arylhydrazines hydrochloride 2 (0.3 mmol, 1.5 equivalent), Eosin Y (0.001 mmol, 5%); subsequently, MeCN (2 mL) was added. The reaction mixture was stirred under an air irradiated by blue LED (λmax = 455 nm) from a 3.0 cm distance for 12 h at room temperature. The mixture was evaporated in a vacuum, then it was purified by silica gel column chromatography with an elution of PE-EA to afford the desired product.

## 5. Conclusions

We developed a green, visible-light-induced method for the C3 arylation and alkylation of quinoxaline-2(1H)-ones and arylation of quinolones, using air as the oxidant and without photocatalysts. This approach offers mild conditions, broad substrate scope, and high yields, aligning with sustainable chemistry principles. ESR studies confirmed singlet oxygen’s role in generating reactive radicals, enabling the efficient transformation of various quinoxaline-2(1H)-one derivative. The method was also applied to the arylation of quinolines and alkylation reactions, showing its versatility and scalability. This work provides a practical and eco-friendly approach for functionalizing quinoxaline-2(1H)-ones and quinolones.

## Figures and Tables

**Table 1:** Reaction condition optimization ^a^.

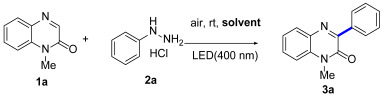		
Entry	Solvent	Yield (%) ^b^
1	Acetone	no
2	CH_3_CN	93
3	Dioxane	43
4	DCM	56
5	EtOH	52
6	DMSO	49
7	DMF	55
8	H_2_O	50
9	THF	35
10	TBME	32
11	Dioxane	40
12	CH_3_CN:EtOH = 1:1	62
13	CH_3_CN:EtOH = 5:1	65
14	HCl:H_2_O = 1:10	45
15 ^c^	CH_3_CN	72
16 ^d^	CH_3_CN	69
17 ^e^	CH_3_CN	72
18 ^f^	CH_3_CN	70
19 ^g^	CH_3_CN	69
20 ^h^	CH_3_CN	0
21 ^i^	CH_3_CN	95
22 ^j^	CH_3_CN	trace
23 ^k^	CH_3_CN	0

^a^ Reaction conditions: **1a** (1.0 equivalent), **2a** (1.0 equivalent), in 2 mL solvent at room temperature, 12 h. ^b^ Yield of isolated product. ^c^ 2.0 equivalent of 1a was used. ^d^ 2.0 equivalent of **2a** was used. ^e^ 3.0 equivalent of **2a** was used. ^f^ The reaction time was 24 h. ^g^ The reaction time was 48 h. ^h^ The reaction was added TEMPO (2.0 equivalent). ^i^ O_2_ instead of air. ^j^ The Reaction light avoidance treatment. ^k^ N_2_ instead of air.

## Data Availability

Data are contained within the article and Supplementary Materials.

## Supplementary Materials

The following supporting information can be downloaded at: https://www.mdpi.com/article/10.3390/molecules29215113/s1, References [57,58,59,60,61] are cited in the Supplementary Materials.
