# Supplementary material for: Visible-Light-Induced Singlet Oxygen-Promoted Arylation and Alkylation of Quinoxalin-2(1H)-ones and Quinolines
Source: Molecules. 2024 Oct 29;29(21):5113. doi: 10.3390/molecules29215113 (PMC11547374; doi:10.3390/molecules29215113)
Supplement: Supplementary file 1 [file molecules-29-05113-s001.zip › molecules-3185885-supplementary.pdf]

Metal-free visible-light-induced C3 arylation and arylation of quinoxalin-2(1H)-ones in the absence of a photosensitizer

Wenbo Wei[a] , Wenbo Liu[a], Yiting Tong[a], Liyuan Ding[a], Guofu Zhong[b,c*], Min Jiang[a*]

Contents

I General information……………………..………………………S2

II Optimization of reaction conditions…………………….…….…………S4

III General procedures and synthesis of quinoxalinones………………S5

IV ESR study of the reaction ……………………………………….....S6

V Characterization of products………………………………………S8

VI References……………………………………….....………....S30

VII ^{1}H and ^{13}C NMR of all the products………………………...…..S31

I General information:

Analytical thin layer chromatography (TLC) was performed using Merck 60 F254 precoated silica gel plate (0.2 mm thickness). Subsequent to elution, plates were visualized using UV radiation (254 nm) on Spectroline Model ENF-24061/F 254 nm.

Flash column chromatography was performed using Merck aluminium oxide90 active neutral with freshly distilled solvents. Columns were typically packed as slurry and equilibrated with the appropriate solvent system prior to use.

Proton nuclear magnetic resonance spectras (^{1}H NMR) were recorded on Bruker AMX 500 spectrophotometer ($CDCl_3$ as solvent). Chemical shifts for ^{1}H NMR spectra are reported as δ in units of parts per million (ppm) downfield from $SiMe_4$ (0.0) and relative to the signal of CDCl3 (7.26, singlet). Multiplicities were given as: s (singlet), d (doublet), t (triplet), dd (doublets of doublet) or m (multiplets). The number of protons (n) for a given resonance is indicated by nH. Coupling constants are reported as a *J* value in Hz. Carbon nuclear magnetic resonance spectra (^{13}C NMR) are reported as δ in units of parts per million (ppm) downfield from $SiMe_4$ (0.0) and relative to the signal of $CDCl_3$ (77.0, triplet).

Electron spin resonance (ESR) spectras were recorded on a JEOL JES X320 spectrometer, X-band (10 GHz).

Purple LEDs (20 W, λ_{max} = 400 nm) were used for irradiation. The light source was placed in 3.0 cm distance from the reaction vessel.

General Procedure

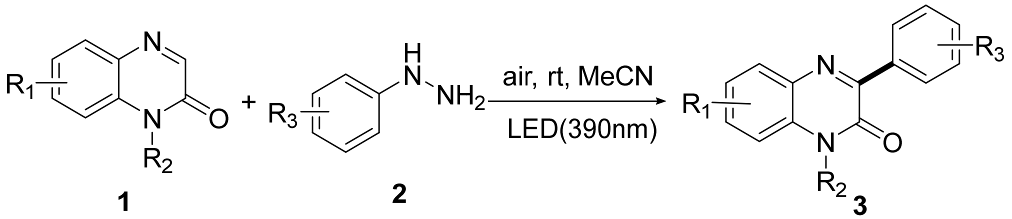

1 2 3

1 (0.15 mmol, 1.0 equiv) and **2** (0.15 mmol, 1.0 equiv) were added into a 3 ml vial. Subsequently, MeCN (2 mL) was added. The reaction mixture was stirred under an air irradiated by purple LED (λ_{max} = 390 nm) from a 3.0 cm distance for 8 h at room temperature. The mixture was evaporated in vacuum, then it was purified by silica gel column chromatography to afford the desired product.

II Optimization of reaction conditions

Table S1 Reaction condition optimization[a]

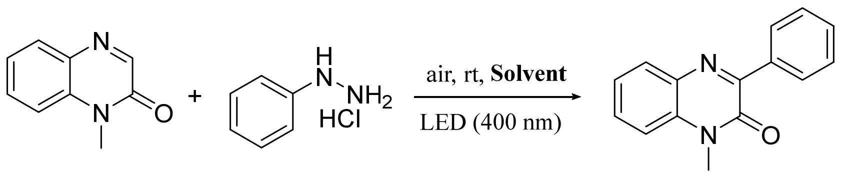

Entry	Solvent	Yield (%)[b]
1	Acetone	no
2	CH_3CN	93
3[c]	CH_3CN	91
4[d]	CH_3CN	93
5[e]	CH_3CN	67
6	Dioxane	43
7	DCM	56
8	EtOH	52
9	DMSO	49
10	DMF	55
11	H_2O	50
12	THF	35
13	TBME	32
14	Dioxane	40
15	CH_3CN:EtOH=1:1	62
16	CH_3CN:EtOH=5:1	65
17	HCl:H_2O=1:10	45
18[g]	CH_3CN	72
19[g]	CH_3CN	69
20[h]	CH_3CN	72
21[i]	CH_3CN	70
22[j]	CH_3CN	69
23[k]	CH_3CN	0
24[l]	CH_3CN	94
25[m]	CH_3CN	trace
26[n]	CH_3CN	0

[a] Reaction conditions: **1a** (1.0 equiv), **2a** (1.0 equiv), in 2 mL solvent at room temperature, 12 h.,[b] 365 nm light was used, [c] 390 nm light was used, [d] 455 nm light was used, [e] Yield of isolated product. [f] 2.0 Equiv of **1a** was used. [g] 2.0 Equiv of **2a** was used. [h] 3.0 Equiv of **2a** was used. [i] The reaction time was 24 h. [j] The reaction time was 48 h. [k] The reaction was added TEMPO. [l] O_2 instead of air. [m] The Reaction light avoidance treatment. [n] N_2 instead of air.

III General procedures and synthesis of Quinoxalinone:

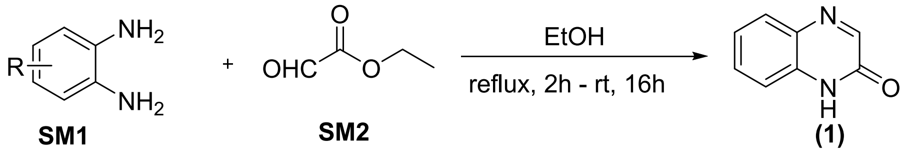

quinoxalin-2(1H)-one (1) was synthesized according to literature [1]**:** A 100 mL Schlenk flask equipped with a stir bar and a septum was flushed with air and charged with EtOH (30 mL). The benzene-1,2-diamine (1.0 equiv.), ethyl acrylate (1.2 equiv.) were added. The mixture was refluxed at 85°C for two hours, then transferred to room temperature and stirred for 16 hours. Monitor the completion of the reaction by TLC.Filter the precipitate and wash the mixture with ethanol and dry to obtain the product.

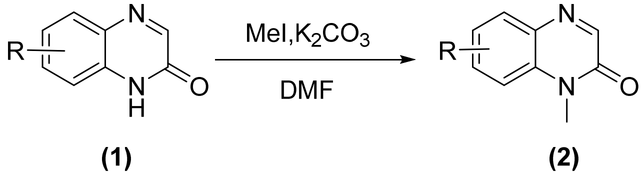

1-methylquinoxalin-2(1H)-one(2): Add a solution of quinoxalin-2(1H)-one (5.0 mmol, 1.0 equiv.) and K_2CO_3 (1.2 equiv.) in DMF (0.25 M) to a 50 mL round-bottomed flask equipped with a magnetic stir bar, follow with addition of MeI (1.6 equiv.).Stir the reaction overnight at room temperature.Quench the mixture with water (20 mL) and extract with EtOAc (40 mL).Wash the organic layer with water (2 x 20 mL), a saturated solution of NH_4Cl (20 mL) and brine (20 mL).Dry the organic layer over $MgSO_4$ and filter.

Concentrate the resulting mixture under reduced pressure to obtain 1-methylquinoxalin-2(1H)-one.

IV Electron Spin Resonance (ESR) study of the reaction

Measurement Conditions: Frequency 9.22 GHz; Power 1.0 mW; Center Field 329.0 mT; Sweep Width 10 mT; Modulation Width 0.1 mT; Amplitude 500; Sweep time 1.0 min; Time constant 0.1s.

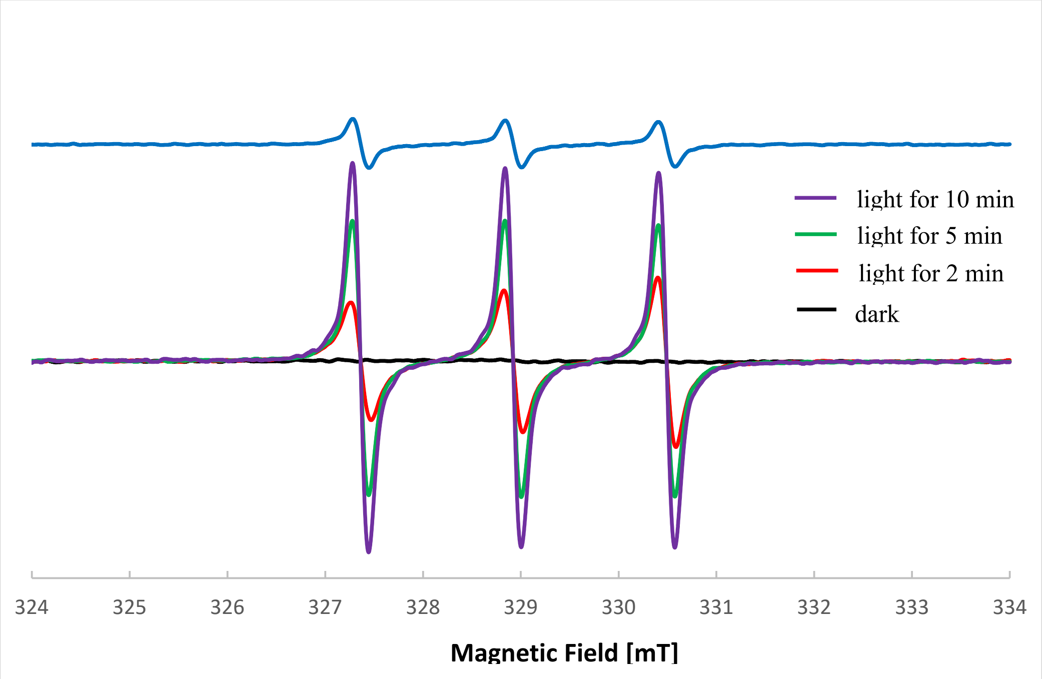

Figure S1. ESR spetras of the singletoxygen produced from 1a under 400 nm irradiation: a mixture (30 uL) of 1a (5 mM) , TEMP (20 mM) in CH_3CN was transferred to a flat cell, then the flat cell was placed into the sample cavity and was irradiated with 400 nm LEDs, the ESR spetras were recorded *in-situ*. A triplet signal was observed as the sample was irradiated for 2 min, 5 min and 10 min, while no signal was recorded under dark (black, red, green and purple lines), with a g = 2.002, AN = 1.56 mT, which was coincident with singletoxygen radical. While the mixture of phenylhydrazine hydrochloride (5 mM), 1a (5 mM) and TEMP (20 mM) was irradiated with 400 nm LEDs for 10 min, a very low strength singnal was observed (blue line), which indicated that the phenylhydrazine hydrochloride would quench singletoxygen.

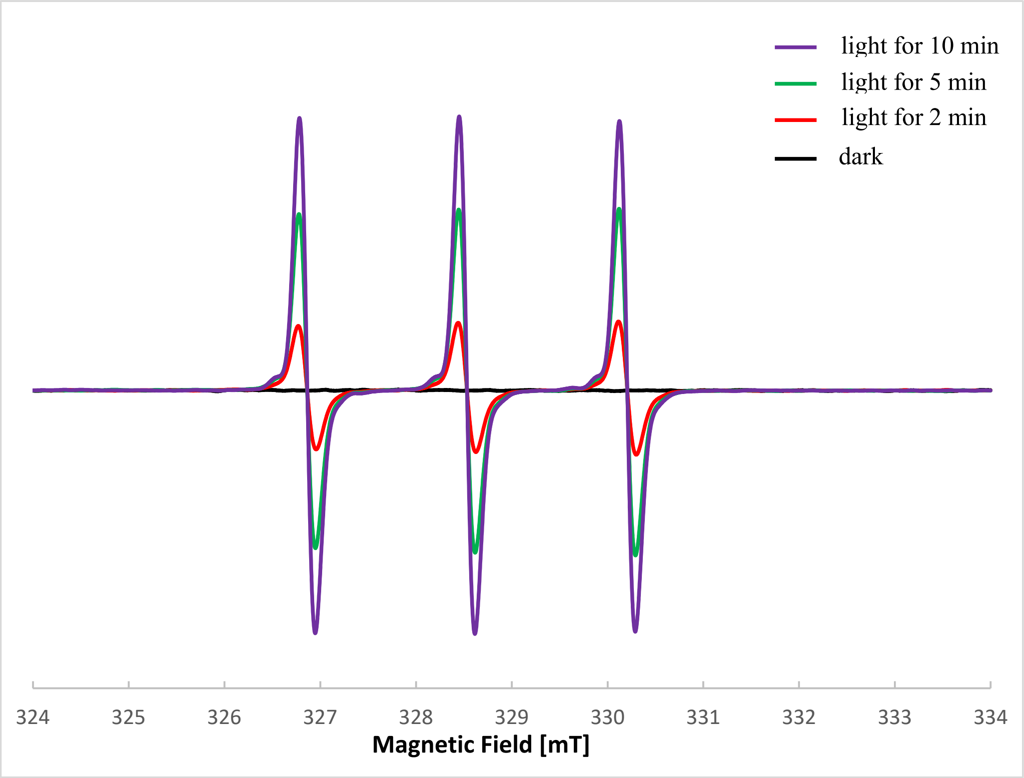

Figure S2. ESR spetras of the singletoxygen produced from 3a under 400 nm irradiation: a mixture (30 uL) of 3a (5 mM) , TEMP (20 mM) in CH_3CN was transferred to a flat cell, then the flat cell was placed into the sample cavity and was irradiated with 400 nm LEDs, the ESR spetras were recorded *in-situ*. A triplet signal was observed as the sample was irradiated for 2 min, 5 min and 10 min, while no signal was recorded under dark (black, red, green and purple lines), with a g = 2.002, AN = 1.56 mT, which was coincident with singletoxygen radical.

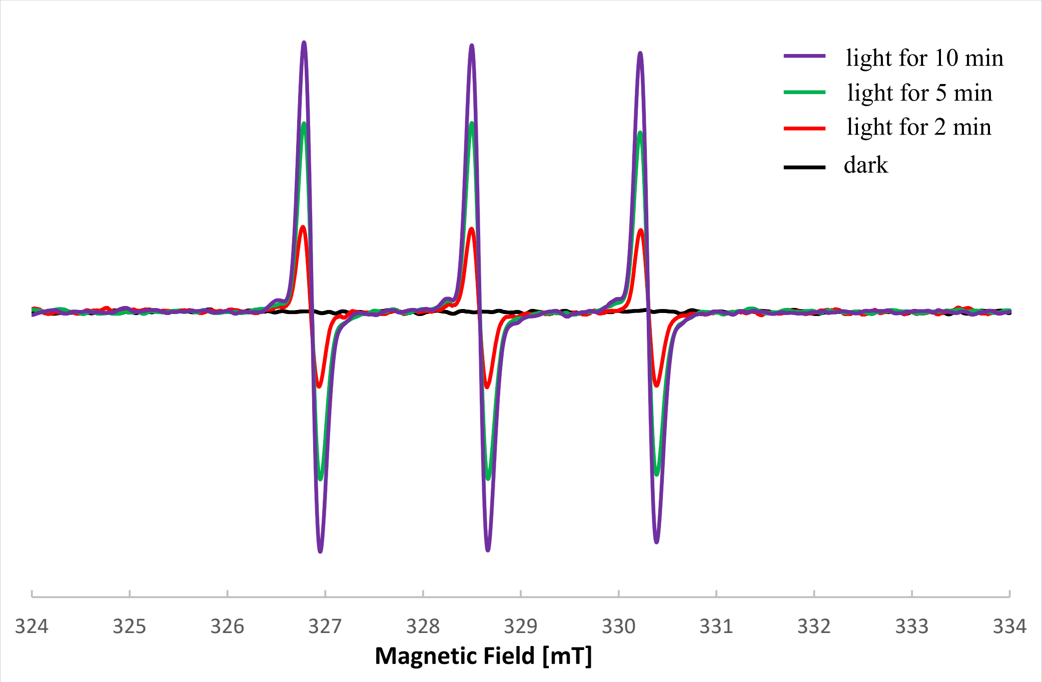

Figure S3. ESR spetras of the singletoxygen produced from Eosin Y under 455 nm irradiation: a mixture (30 uL) of Eosin Y (1 mM) , TEMP (20 mM) in CH_3CN was transferred to a flat cell, then the flat cell was placed into the sample cavity and was irradiated with 455 nm LEDs, the ESR spetras were recorded *in-situ*. A triplet signal was observed as the sample was irradiated for 2 min, 5 min and 10 min, while no signal was recorded under dark (black, red, green and purple lines), with a g = 2.002, AN = 1.56 mT, which was coincident with singletoxygen radical.

V Characterization of products:

1-methyl-3-phenylquinoxalin-2(1H)-one (3a) [1]

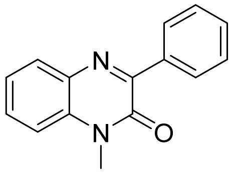

3a

The title compound was prepared according to the typical procedure, as described above, in 93% yield, as yellow solid, 137-138 °C.

^{1}H NMR (500 MHz, $CDCl_3$) δ 8.32 – 8.29 (m, 2H), 7.95 (dd, J = 8.0, 1.5 Hz, 1H), 7.59 – 7.55 (m, 1H), 7.50 – 7.47 (m, 3H), 7.39 – 7.35 (m, 1H), 7.33 (dd, J = 8.4, 1.2 Hz, 1H), 3.77 (s, 3H).

^{13}C NMR (125 MHz, $CDCl_3$) δ 154.74, 154.21, 136.10, 133.39, 133.13, 130.49, 130.33, 129.56, 128.09, 123.74, 113.59, 29.31.

HRMS (ESI+) calcd for $C_{15}H_{13}N_2O^+$ $(M+H)^+$, m/z 237.1023, found 237.1025.

6,7-difluoro-1-methyl-3-phenylquinoxalin-2(1H)-one (3b) [1]

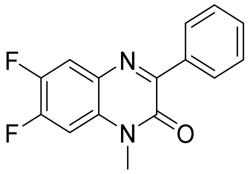

3b

The title compound was prepared according to the typical procedure, as described above, in 87% yield, as yellow solid, 111-113 °C.

^{1}H NMR (500 MHz, $CDCl_3$) δ 8.29 (t, J = 1.8 Hz, 1H), 8.28 (d, J = 2.5 Hz, 1H), 7.74 (dd, J = 10.2, 8.2 Hz, 1H), 7.50 – 7.46 (m, 3H), 7.13 (dd, J = 11.3, 7.0 Hz, 1H), 3.72 (s, 3H).

^{13}C NMR (125 MHz, $CDCl_3$) δ 154.40, 154.29, 152.59 (d, J = 13.7 Hz), 150.57 (d, J = 13.7 Hz), 147.81 (d, J = 13.7 Hz), 145.85 (d, J = 15.0 Hz), 135.51, 130.72, 129.54, 128.16, 117.86 (d, J = 17.5 Hz), 102.20 (d, J = 22.5 Hz), 29.85.

^{19}F NMR (471 MHz, $CDCl_3$) δ -130.45 (d, J = 23.5 Hz, 1F), -141.91 (d, J = 23.5 Hz, 1F).

HRMS (ESI+) calcd for $C_{15}H_{11}F_2N_2O^+$ $(M+H)^+$, m/z 273.0832, found 273.0834.

6,7-dichloro-1-methyl-3-phenylquinoxalin-2(1H)-one (3c) [2]

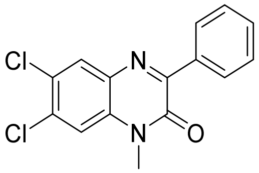

3c

The title compound was prepared according to the typical procedure, as described above, in 76% yield, as yellow solid, 120-121 °C.

^{1}H NMR (500 MHz, $CDCl_3$) δ 8.31 (t, J = 1.6 Hz, 1H), 8.29 (t, J = 2.0 Hz, 1H), 8.01 (s, 1H), 7.51 – 7.49 (m, 2H), 7.48 – 7.46 (m, 1H), 7.41 (s, 1H), 3.71 (s, 3H).

^{13}C NMR (125 MHz, $CDCl_3$) δ 155.14, 154.13, 135.38, 134.31, 132.72, 132.16, 131.10, 130.94, 129.66, 128.18, 127.53, 115.07, 29.58.

HRMS (ESI+) calcd for $C_{15}H_{11}Cl_2N_2O^+$ $(M+H)^+$, m/z 305.0243, found 305.0245.

6,7-dibromo-1-methyl-3-phenylquinoxalin-2(1H)-one (3d) [1]

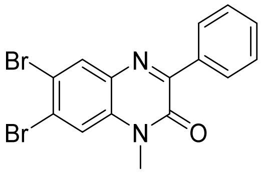

3d

The title compound was prepared according to the typical procedure, as described above, in 61% yield, as yellow solid, 131-133 °C.

^{1}H NMR (500 MHz, $CDCl_3$) δ 8.30 (d, J = 1.9 Hz, 1H), 8.29 (d, J = 1.9 Hz, 1H), 8.01 (s, 1H), 7.51 – 7.46 (m, 3H), 7.41 (s, 1H), 3.71 (s, 3H).

^{13}C NMR (125 MHz, $CDCl_3$) δ 155.14, 154.13, 135.38, 134.31, 132.72, 132.16, 131.10, 130.94, 129.66, 128.18, 127.53, 115.07, 29.58.

HRMS (ESI+) calcd for $C_{15}H_{11}Br_2N_2O^+$ $(M+H)^+$, m/z 394.9213, found 394.9215.

1,6,7-trimethyl-3-phenylquinoxalin-2(1H)-one (3e) [1]

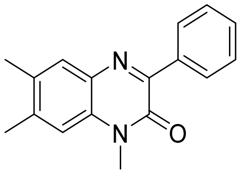

3e

The title compound was prepared according to the typical procedure, as described above, in 90% yield,as yellow solid, 108-109 °C.

^{1}H NMR (500 MHz, $CDCl_3$) δ 8.31 – 8.27 (m, 2H), 7.70 (s, 1H), 7.49 – 7.44 (m, 3H), 7.10 (s, 1H), 3.74 (s, 3H), 2.44 (s, 3H), 2.37 (s, 3H).

^{13}C NMR (125 MHz, $CDCl_3$) δ 154.82, 152.98, 140.30, 136.40, 132.67, 131.61, 131.45, 130.51, 129.98, 129.44, 128.02, 114.15, 29.21, 20.66, 19.22.

HRMS (ESI+) calcd for $C_{17}H_{17}N_2O^+$ $(M+H)^+$, m/z 265.1336, found 265.1338.

1-methyl-3-phenyl-7-(trifluoromethyl)quinoxalin-2(1H)-one (3f) [1]

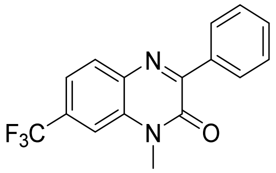

3f

The title compound was prepared according to the typical procedure, as described above, in 76% yield, as yellow solid, 117-118 °C.

^{1}H NMR (500 MHz, $CDCl_3$) δ 8.34 (d, J = 1.6 Hz, 1H), 8.33 (d, J = 2.2 Hz, 1H), 8.23 (d, J = 2.0 Hz, 1H), 7.78 (dd, J = 8.9, 2.1 Hz, 1H), 7.52 – 7.48 (m, 3H), 7.43 (d, J = 8.7 Hz, 1H), 3.79 (s, 3H).

^{13}C NMR (125 MHz, $CDCl_3$) δ 155.48, 154.53, 135.60, 135.41, 132.43, 130.93, 129.66, 128.20, 127.84 (q, J = 3.7 Hz), 126.49 (q, J = 3.7 Hz), 126.21, 123.77 (q, J = 271.2 Hz), 114.23, 29.58.

^{19}F NMR (471 MHz, $CDCl_3$) δ -61.99 (s, 3F).

HRMS (ESI+) calcd for $C_{16}H_{12}F_3N_2O^+$ $(M+H)^+$, m/z 305.0897, found 305.0899.

6-chloro-1-methyl-3-phenylquinoxalin-2(1H)-one (3g) [1]

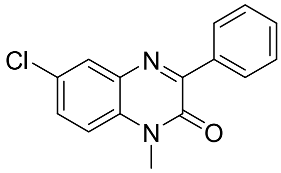

3g

The title compound was prepared according to the typical procedure, as described above, in 70% yield, as yellow solid, 115-117 °C.

^{1}H NMR (500 MHz, $CDCl_3$) δ 8.32 – 8.30 (m, 2H), 7.94 (d, J = 2.4 Hz, 1H), 7.53 – 7.51 (m, 1H), 7.51 – 7.48 (m, 3H), 7.48 – 7.28 (m, 1H), 3.76 (s, 3H).

^{13}C NMR (125 MHz, $CDCl_3$) δ 155.26, 154.40, 135.67, 133.63, 132.08, 130.74, 130.25, 129.69, 129.66, 129.05, 128.14, 114.72, 29.51.

HRMS (ESI+) calcd for $C_{15}H_{12}ClN_2O^+$ $(M+H)^+$, m/z 271.0633, found 271.0635.

7-chloro-1-methyl-3-phenylquinoxalin-2(1H)-one (3h) [1]

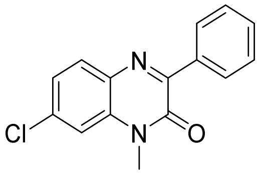

3h

The title compound was prepared according to the typical procedure, as described above, in 75%

yield, as yellow solid, 131-132 °C.

^{1}H NMR (500 MHz, $CDCl_3$) δ 8.31 – 8.27 (m, 2H), 7.86 (d, J = 9.1 Hz, 1H), 7.50 – 7.46 (m, 2H), 7.34 – 7.31 (m, 2H), 3.74 (s, 3H).

^{13}C NMR (125 MHz, $CDCl_3$) δ 154.44, 154.09, 136.26, 135.74, 134.20, 131.63, 131.49, 130.58, 129.55, 128.14, 124.17, 113.64, 29.44.

HRMS (ESI+) calcd for $C_{15}H_{12}ClN_2O^+$ $(M+H)^+$, m/z 271.0633, found 271.0635.

6-bromo-1-methyl-3-phenylquinoxalin-2(1H)-one (3i) [1]

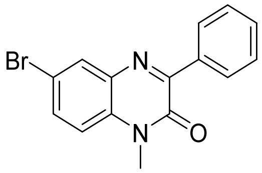

3i

The title compound was prepared according to the typical procedure, as described above, in 80% yield, as yellow solid, 135-137 °C.

^{1}H NMR (500 MHz, $CDCl_3$) δ 8.32 (d, J = 1.7 Hz, 1H), 8.30 (d, J = 2.9 Hz, 1H), 8.09 (d, J = 2.3 Hz, 1H), 7.64 (dd, J = 8.9, 2.3 Hz, 1H), 7.50 (d, J = 6.6 Hz, 2H), 7.47 (d, J = 8.9 Hz, 1H), 7.20 (d, J = 8.9 Hz, 1H), 3.74 (s, 3H).

^{13}C NMR (125 MHz, $CDCl_3$) δ 155.15, 154.39, 135.64, 133.93, 132.96, 132.74, 132.50, 130.75, 129.66, 128.14, 116.27, 115.01, 29.48.

HRMS (ESI+) calcd for $C_{15}H_{12}Br_2N_2O^+$ $(M+H)^+$, m/z 315.0128, found 315.0130.

7-bromo-1-methyl-3-phenylquinoxalin-2(1H)-one (3j) [2]

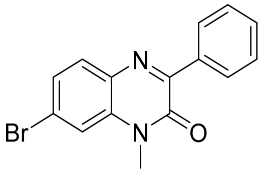

3j

The title compound was prepared according to the typical procedure, as described above, in 75% yield, as yellow solid, 139-140 °C.

^{1}H NMR (500 MHz, $CDCl_3$) δ 8.30 (d, J = 1.8 Hz, 1H), 8.29 (d, J = 2.9 Hz, 1H), 7.78 (d, J = 8.5 Hz, 1H), 7.50 – 7.46 (m, 5H), 3.73 (s, 3H).

^{13}C NMR (125 MHz, $CDCl_3$) δ 154.38, 154.31, 135.74, 134.35, 131.94, 131.62, 130.61, 129.55, 128.15, 127.04, 124.41, 116.65, 29.44.

HRMS (ESI+) calcd for $C_{15}H_{12}BrN_2O^+$ $(M+H)^+$, m/z 315.0128, found 315.0130.

1,5-dimethyl-3-phenylquinoxalin-2(1H)-one (3k) [2]

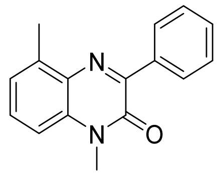

3k

The title compound was prepared according to the typical procedure, as described above, in 94% yield, as yellow solid, 107-109 °C.

^{1}H NMR (500 MHz, CDCl$_3$) δ 8.44 – 8.40 (m, 2H), 7.50 – 7.47 (m, 3H), 7.46 – 7.43 (m, 1H), 7.22 (d, *J* = 7.4 Hz, 1H), 7.17 (d, *J* = 8.4 Hz, 1H), 3.76 (s, 3H), 2.76 (s, 3H).

^{13}C NMR (125 MHz, CDCl$_3$) δ 154.66, 151.72, 139.31, 136.54, 133.49, 131.64, 130.16, 130.10, 129.69, 128.02, 124.95, 111.49, 29.44, 17.61.

HRMS (ESI+) calcd for $C_{16}H_{15}N_2O^+$ $(M+H)^+$, m/z 251.1179, found 251.1181.

6-methoxy-1-methyl-3-phenylquinoxalin-2(1H)-one (3l) [3]

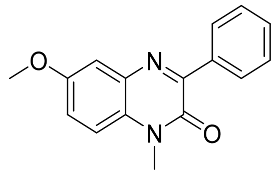

3l

The title compound was prepared according to the typical procedure, as described above, in 80% yield, as yellow solid, 116-117 °C.

^{1}H NMR (500 MHz, CDCl3) δ 8.32 – 8.28 (m, 2H), 7.48 (q, *J* = 3.0 Hz, 3H), 7.42 (t, *J* = 3.0 Hz, 1H), 7.26 (t, *J* = 2.9 Hz, 1H), 7.20 (dt, *J* = 9.1, 3.0 Hz, 1H), 3.91 (s, 3H), 3.77 (s, 3H).

^{13}C NMR (125 MHz, CDCl$_3$) δ 156.07, 154.67, 154.41, 136.21, 133.87, 130.31, 129.55, 128.09, 127.73, 119.75, 114.51, 111.62, 55.81, 29.46.

HRMS (ESI+) calcd for $C_{16}H_{15}N_2O_2^+$ $(M+H)^+$, m/z 267.1129, found 267.1131.

1-methyl-2-oxo-3-phenyl-1,2-dihydroquinoxaline-6-carbonitrile (3m) [3]

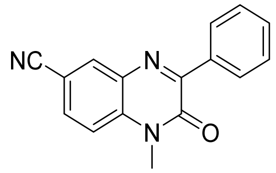

3m

The title compound was prepared according to the typical procedure, as described above, in 90% yield, as yellow solid, 198-199 °C.

^{1}H NMR (500 MHz, $CDCl_3$) δ 8.34 (d, *J* = 1.5 Hz, 1H), 8.32 (d, *J* = 1.9 Hz, 1H), 8.25 (d, *J* = 1.9 Hz, 1H), 7.79 (dd, *J* = 8.7, 1.9 Hz, 1H), 7.55 – 7.51 (m, 2H), 7.50 (d, *J* = 1.6 Hz, 1H), 7.41 (d, *J* = 8.7 Hz, 1H), 3.79 (s, 3H).

^{13}C NMR (125 MHz, $CDCl_3$) δ 155.93, 136.53, 135.10, 134.67, 132.64, 131.22, 129.73, 128.26, 118.03, 116.82, 114.71, 107.30, 29.62.

HRMS (ESI+) calcd for $C_{16}H_{12}N_3O^+$ $(M+H)^+$, m/z 262.0975, found 262.0977.

3-phenylquinoxalin-2(1H)-one (3n) [1]

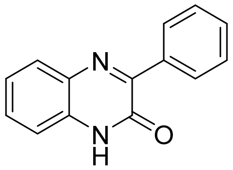

3n

The title compound was prepared according to the typical procedure, as described above, in 62% yield, as yellow solid, 239-240 °C.

^{1}H NMR (500 MHz, DMSO-d_6) δ 12.58 (s, 1H), 8.30 (dd, *J* = 6.5, 3.2 Hz, 2H), 7.84 (d, *J* = 7.4 Hz, 1H), 7.57 – 7.53 (m, 1H), 7.53 – 7.48 (m, 3H), 7.36 – 7.32 (m, 2H).

^{13}C NMR (125 MHz, DMSO-d_6) δ 155.06, 154.63, 136.10, 132.54, 132.49, 130.80, 130.67, 129.68, 129.24, 128.34, 123.87, 115.58.

HRMS (ESI+) calcd for $C_{14}H_{11}N_2O^+$ $(M+H)^+$, m/z 223.0866, found 223.0868.

1-butyl-3-phenylquinoxalin-2(1H)-one (3o) [4]

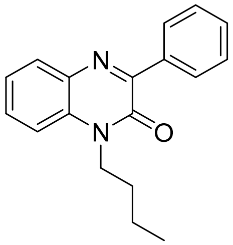

3o

The title compound was prepared according to the typical procedure, as described above, in 86% yield, as yellow solid, 146-147 °C.

^{1}H NMR (500 MHz, $CDCl_3$) 8.32 – 8.30 (m, 2H), 7.96 (dd, *J* = 8.0, 1.6 Hz, 1H), 7.56 (ddd, *J* = 8.6, 7.3, 1.6 Hz, 1H), 7.49 – 7.47 (m, 3H), 7.37 – 7.33 (m, 2H), 4.32 (t, *J* = 7.4 Hz, 2H), 1.83 – 1.77 (m, 2H), 1.53 (q, *J* = 7.5 Hz, 2H), 1.02 (t, *J* = 7.4 Hz, 3H).

^{13}C NMR (125 MHz, $CDCl_3$) δ 154.44, 154.17, 136.11, 133.43, 132.61, 130.73, 130.28, 130.23, 129.60, 128.07, 123.52, 113.59, 42.43, 29.35, 20.37, 13.83.

HRMS (ESI+) calcd for $C_{18}H_{19}N_2O^+$ $(M+H)^+$, m/z 279.1492, found 279.1494.

1-(cyclohexylmethyl)-3-phenylquinoxalin-2(1H)-one (3p)[4]

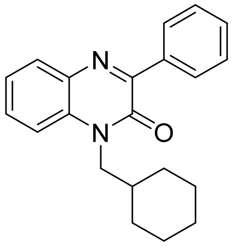

3p

The title compound was prepared according to the typical procedure, as described above, in 77% yield, as yellow solid, 143-145 °C.

^{1}H NMR (500 MHz, $CDCl_3$) δ 8.30 (dd, *J* = 6.6, 3.0 Hz, 2H), 7.95 (d, *J* = 7.5 Hz, 1H), 7.55 (t, *J* = 8.7 Hz, 1H), 7.48 (p, *J* = 3.6 Hz, 3H), 7.35 (t, *J* = 8.2 Hz, 2H), 4.22 (d, *J* = 7.2 Hz, 2H), 1.99 – 1.93 (m, 4H), 1.75 – 1.70 (m, 4H), 1.67 – 1.60 (m, 2H), 1.20 (d, *J* = 8.1 Hz, 4H).

^{13}C NMR (125 MHz, $CDCl_3$) δ 154.90, 154.23, 136.18, 133.37, 133.05, 130.71, 130.28, 130.09, 129.61, 128.06, 123.48, 114.10, 48.29, 36.65, 31.01, 26.20, 25.85.

HRMS (ESI+) calcd for $C_{21}H_{23}N_2O^+$ $(M+H)^+$, m/z 319.1805, found 319.1807.

ethyl 2-(2-oxo-3-phenylquinoxalin-1(2H)-yl)acetate (3q) [4]

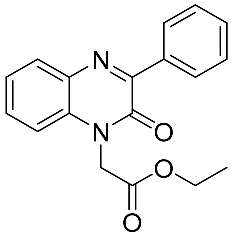

3q

The title compound was prepared according to the typical procedure, as described above, in 65% yield, as yellow solid, 187-188 °C.

^{1}H NMR (500 MHz, $CDCl_3$) δ 8.20 (dd, *J* = 8.0, 1.7 Hz, 2H), 8.09 (dd, *J* = 8.2, 1.6 Hz, 1H), 7.82 (dd, *J* = 8.2, 1.5 Hz, 1H), 7.65 (ddd, *J* = 8.3, 7.0, 1.5 Hz, 1H), 7.60 (ddd, *J* = 8.3, 7.0, 1.5 Hz, 1H), 7.54 – 7.48 (m, 3H), 5.12 (s, 2H), 4.28 (q, *J* = 7.1 Hz, 2H), 1.30 (t, *J* = 7.2 Hz, 3H).

^{13}C NMR (125 MHz, $CDCl_3$) δ 168.57, 154.15, 146.40, 139.50, 139.30, 135.78, 129.82, 129.80, 129.77, 129.04, 128.29, 127.24, 126.77, 62.88, 61.30, 14.20.

HRMS (ESI+) calcd for $C_{18}H_{17}N_2O_3^+$ $(M+H)^+$, m/z 309.1234, found 309.1236.

1-(4-fluorobenzyl)-3-phenylquinoxalin-2(1H)-one (3r) [5]

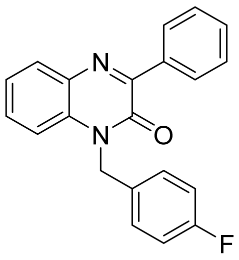

3r

The title compound was prepared according to the typical procedure, as described above, in 80% yield, as yellow solid, 175-176 °C.

^{1}H NMR (500 MHz, $CDCl_3$) δ 8.38 – 8.33 (m, 2H), 7.97 (dd, J = 8.0, 1.5 Hz, 1H), 7.51 – 7.49 (m, 3H), 7.49 – 7.46 (m, 1H), 7.37 – 7.33 (m, 1H), 7.31 – 7.27 (m, 3H), 7.01 (t, J = 8.6 Hz, 2H), 5.54 (s, 2H).
^{13}C NMR (125 MHz, $CDCl_3$) δ 163.23, 161.27, 154.76, 154.25, 135.93, 133.42, 132.59, 131.13 (d, J = 2.5 Hz), 130.74, 130.52, 130.37, 129.63, 128.88 (d, J = 7.5 Hz), 128.15, 123.94, 115.90 (d, J = 21.2 Hz), 114.12, 45.50.

^{19}F NMR (471 MHz, $CDCl_3$) δ -114.38.

HRMS (ESI+) calcd for $C_{21}H_{16}FN_2O^+$ $(M+H)^+$, m/z 331.1242, found 331.1244.

1-(3-fluorobenzyl)-3-phenylquinoxalin-2(1H)-one (3s) [4]

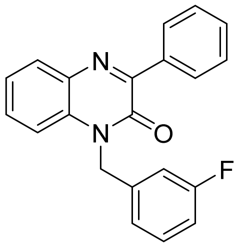

3s

The title compound was prepared according to the typical procedure, as described above, in 83% yield, as yellow solid, 161-162 °C.

^{1}H NMR (500 MHz, $CDCl_3$) δ 8.38 – 8.34 (m, 2H), 7.98 (dd, J = 8.0, 1.5 Hz, 1H), 7.52 – 7.45 (m, 4H), 7.35 (t, J = 7.4 Hz, 1H), 7.30 (td, J = 8.0, 5.9 Hz, 1H), 7.24 (d, J = 8.4 Hz, 1H), 7.08 (d, J = 7.7 Hz, 1H), 7.00 – 6.95 (m, 2H), 5.56 (s, 2H).
^{13}C NMR (125 MHz, $CDCl_3$) δ 164.14, 162.18, 154.72, 154.20, 137.89 (d, J = 7.5 Hz), 135.89, 133.39, 132.57, 130.74, 130.57 (d, J = 8.7 Hz), 130.54, 130.43, 129.65, 128.16, 124.01, 122.58 (d, J = 2.5 Hz), 114.78 (d, J = 20.0 Hz), 114.11, 114.06 (d, J = 22.5 Hz), 45.70.

^{19}F NMR (471 MHz, $CDCl_3$) δ -112.05.

HRMS (ESI+) calcd for $C_{21}H_{16}FN_2O^+$ $(M+H)^+$, m/z 331.1242, found 331.1244.

1-methyl-3-(p-tolyl)quinoxalin-2(1H)-one (3t) [1]

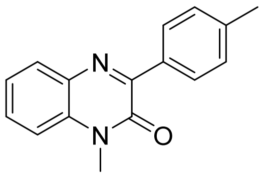

3t

The title compound was prepared according to the typical procedure, as described above, in 74% yield, as yellow solid, 123-124 °C.

^{1}H NMR (500 MHz, $CDCl_3$) δ 8.24 (d, J = 8.3 Hz, 2H), 7.93 (dd, J = 7.9, 1.5 Hz, 1H), 7.55 (ddd, J = 8.5, 7.2, 1.5 Hz, 1H), 7.36 (t, J = 7.6 Hz, 1H), 7.33 (d, J = 8.3 Hz, 1H), 7.29 (d, J = 8.0 Hz, 2H), 3.77 (s, 3H), 2.42 (s, 3H).

^{13}C NMR (125 MHz, $CDCl_3$) δ 154.80, 154.05, 140.63, 133.35, 133.31, 133.17, 130.36, 130.05, 129.53, 128.83, 123.66, 113.53, 29.28, 21.53.

HRMS (ESI+) calcd for $C_{16}H_{15}N_2O^+$ $(M+H)^+$, m/z 251.1179, found 251.1181.

1-methyl-3-(o-tolyl)quinoxalin-2(1H)-one (3u) [1]

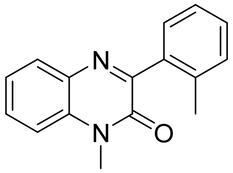

3u

The title compound was prepared according to the typical procedure, as described above, in 71% yield, as yellow solid, 106-107 °C.

^{1}H NMR (500 MHz, $CDCl_3$) δ 7.93 (dd, J = 8.4, 1.4 Hz, 1H), 7.63 – 7.60 (m, 1H), 7.46 (dd, J = 8.1, 1.5 Hz, 1H), 7.40 – 7.37 (m, 2H), 7.36 – 7.34 (m, 1H), 7.29 (d, J = 7.3 Hz, 2H), 3.78 (s, 3H), 2.35 (s, 3H).

^{13}C NMR (125 MHz, $CDCl_3$) δ 158.44, 154.57, 136.85, 136.13, 133.60, 132.91, 130.58, 130.54, 130.47, 129.35, 129.18, 125.63, 123.76, 113.70, 29.39, 19.95.

HRMS (ESI+) calcd for $C_{16}H_{15}N_2O^+$ $(M+H)^+$, m/z 251.1179, found 251.1181.

3-(4-isopropylphenyl)-1-methylquinoxalin-2(1H)-one (3v) [4]

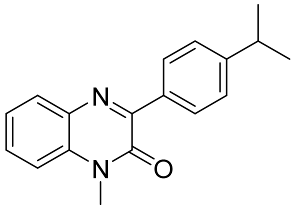

3v

The title compound was prepared according to the typical procedure, as described above, in 75% yield, as yellow solid, 127-128 °C.

^{1}H NMR (500 MHz, $CDCl_3$) δ 8.25 (d, J = 1.9 Hz, 1H), 8.23 (d, J = 1.9 Hz, 1H), 7.93 (dd, J = 7.9, 1.5 Hz, 1H), 7.55 (ddd, J = 8.6, 7.3, 1.5 Hz, 1H), 7.37 (dd, J = 7.6, 1.6 Hz, 1H), 7.35 – 7.32 (m, 3H), 3.77 (s, 3H), 2.98 (p, J = 7.0 Hz, 1H), 1.29 (d, J = 6.9 Hz, 6H).
^{13}C NMR (125 MHz, $CDCl_3$) δ 154.81, 154.25, 151.46, 133.74, 133.32, 133.22, 130.38, 130.05, 129.59, 126.26, 123.67, 113.54, 34.18, 29.29, 23.85.

HRMS (ESI+) calcd for $C_{18}H_{19}N_2O^+$ $(M+H)^+$, m/z 279.1492, found 279.1494.

3-(4-methoxyphenyl)-1-methylquinoxalin-2(1H)-one (3w) [4]

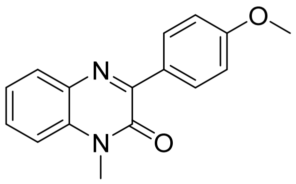

3w

The title compound was prepared according to the typical procedure, as described above, in 80 % yield, as a yellow solid, 139-140 °C.
^{1}H NMR (500 MHz, $CDCl_3$) δ 8.40 (d, J = 2.1 Hz, 1H), 8.39 (d, J = 2.1 Hz, 1H), 7.91 (dd, J = 8.0, 1.5 Hz, 1H), 7.54 (ddd, J = 8.5, 7.3, 1.5 Hz, 1H), 7.36 (ddd, J = 8.2, 7.3, 1.2 Hz, 1H), 7.32 (dd, J = 8.4, 1.2 Hz, 1H), 7.00 (d, J = 2.1 Hz, 1H), 6.99 (d, J = 2.1 Hz, 1H), 3.88 (s, 3H), 3.77 (s, 3H).
^{13}C NMR (125 MHz, $CDCl_3$) δ 161.50, 154.87, 153.23, 133.20, 133.15, 131.38, 130.16, 129.76, 128.78, 123.66, 113.51, 113.49, 55.39, 29.28.

HRMS (ESI+) calcd for $C_{16}H_{15}N_2O_2^+$ $(M+H)^+$, m/z 267.1129, found 267.1131.

3-(4-chlorophenyl)-1-methylquinoxalin-2(1H)-one (3x) [4]

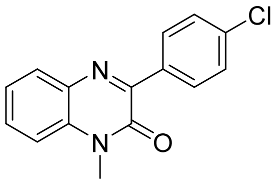

3x

The title compound was prepared according to the typical procedure, as described above, in 95% yield, as yellow solid, 121-122 °C.

^{1}H NMR (500 MHz, $CDCl_3$) δ 8.35 (d, J = 2.0 Hz, 1H), 8.33 (d, J = 2.0 Hz, 1H), 7.93 (dd, J = 8.1, 1.5 Hz, 1H), 7.58 (ddd, J = 8.5, 7.2, 1.5 Hz, 1H), 7.45 (d, J = 2.0 Hz, 1H), 7.44 (d, J = 2.0 Hz, 1H), 7.38 (ddd, J = 8.3, 7.2, 1.2 Hz, 1H), 7.34 (dd, J = 8.4, 1.2 Hz, 1H), 3.77 (s, 3H).
^{13}C NMR (125 MHz, $CDCl_3$) δ 154.62, 152.67, 136.56, 134.46, 133.38, 133.01, 131.00, 130.59, 130.52, 128.30, 123.89, 113.64, 29.34.

HRMS (ESI+) calcd for $C_{15}H_{12}ClN_2O^+$ $(M+H)^+$, m/z 271.0633, found 271.0635.

3-(3-chlorophenyl)-1-methylquinoxalin-2(1H)-one (3y) [5]

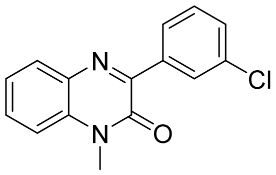

3y

The title compound was prepared according to the typical procedure, as described above, in 90% yield, as yellow solid, 114-115 °C.

^{1}H NMR (500 MHz, $CDCl_3$) δ 8.37 (t, *J* = 1.9 Hz, 1H), 8.28 (d, *J* = 7.6 Hz, 1H), 7.95 (d, *J* = 8.3 Hz, 1H), 7.60 (t, *J* = 8.2 Hz, 1H), 7.45 (d, *J* = 8.0 Hz, 1H), 7.42 (d, *J* = 8.0 Hz, 1H), 7.38 (d, *J* = 8.4 Hz, 1H), 7.35 (d, *J* = 8.9 Hz, 1H), 3.78 (s, 3H).

^{13}C NMR (125 MHz, $CDCl_3$) δ 154.53, 152.45, 137.68, 134.13, 133.45, 132.96, 130.81, 130.65, 130.32, 129.56, 129.31, 127.79, 123.92, 113.66, 29.36.

HRMS (ESI+) calcd for $C_{15}H_{12}ClN_2O^+$ $(M+H)^+$, m/z 271.0633, found 271.0635.

3-(2-chlorophenyl)-1-methylquinoxalin-2(1H)-one (3z) [5]

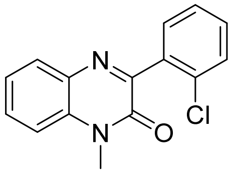

3z

The title compound was prepared according to the typical procedure, as described above, in 82% yield, as yellow solid, 135-137 °C.

^{1}H NMR (500 MHz, $CDCl_3$) δ 7.95 (dd, *J* = 7.8, 2.0 Hz, 1H), 7.65 – 7.62 (m, 1H), 7.50 (td, *J* = 6.8, 2.9 Hz, 2H), 7.42 – 7.38 (m, 4H), 3.78 (s, 3H).

^{13}C NMR (125 MHz, $CDCl_3$) δ 156.41, 154.08, 135.87, 133.83, 133.29, 132.81, 131.04, 130.67, 130.54, 130.51, 129.80, 126.84, 123.88, 113.83, 29.43.

HRMS (ESI+) calcd for $C_{15}H_{12}ClN_2O^+$ $(M+H)^+$, m/z 271.0633, found 271.0635.

3-(4-bromophenyl)-1-methylquinoxalin-2(1H)-one (3aa) [1]

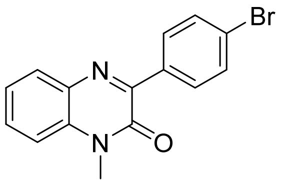

3aa

The title compound was prepared according to the typical procedure, as described above, in 78% yield, as yellow solid, 149-150 °C.

^{1}H NMR (500 MHz, $CDCl_3$) δ 8.28 (d, J = 2.0 Hz, 1H), 8.27 (d, J = 1.9 Hz, 1H), 7.93 (dd, J = 8.0, 1.5 Hz, 1H), 7.61 (d, J = 2.0 Hz, 1H), 7.60 (d, J = 1.7 Hz, 1H), 7.60 – 7.57 (m, 1H), 7.38 (t, J = 7.6 Hz, 1H), 7.34 (d, J = 8.4 Hz, 1H), 3.77 (s, 3H).

^{13}C NMR (125 MHz, $CDCl_3$) δ 154.58, 152.76, 134.91, 133.40, 133.02, 131.27, 131.22, 130.62, 130.54, 125.12, 123.89, 113.65, 29.34.

HRMS (ESI+) calcd for $C_{15}H_{12}BrN_2O^+$ $(M+H)^+$, m/z 315.0128, found 315.0130.

3-(2-bromophenyl)-1-methylquinoxalin-2(1H)-one (3ab) [1]

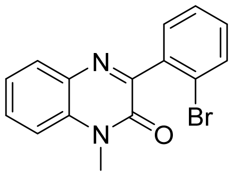

3ab

The title compound was prepared according to the typical procedure, as described above, in 86% yield, as yellow solid, 120-121 °C.

^{1}H NMR (500 MHz, $CDCl_3$) δ 7.95 (d, J = 7.5 Hz, 1H), 7.68 (d, J = 8.0 Hz, 1H), 7.64 (td, J = 7.8, 1.6 Hz, 1H), 7.48 (dd, J = 7.5, 1.9 Hz, 1H), 7.45 – 7.39 (m, 3H), 7.33 (td, J = 7.7, 1.8 Hz, 1H), 3.78 (s, 3H).

^{13}C NMR (125 MHz, $CDCl_3$) δ 157.43, 153.96, 137.79, 133.86, 132.94, 132.75, 131.04, 130.67, 130.64, 130.49, 127.43, 123.90, 122.41, 113.84, 29.45.

HRMS (ESI+) calcd for $C_{15}H_{12}BrN_2O^+$ $(M+H)^+$, m/z 315.0128, found 315.0130.

1-methyl-3-(4-(trifluoromethyl)phenyl)quinoxalin-2(1H)-one (3ac) [4]

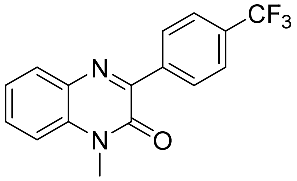

3ac

The title compound was prepared according to the typical procedure, as described above, in 79 % yield, as yellow solid, 126-127 °C.

^{1}H NMR (500 MHz, $CDCl_3$) δ 8.46 (d, J = 8.0 Hz, 2H), 7.96 (dd, J = 8.1, 1.5 Hz, 1H), 7.73 (d, J = 8.1 Hz, 2H), 7.62 (ddd, J = 8.6, 7.3, 1.5 Hz, 1H), 7.41 (ddd, J = 8.2, 7.3, 1.2 Hz, 1H), 7.37 (dd, J = 8.4, 1.2 Hz, 1H), 3.79 (s, 3H).

^{13}C NMR (125 MHz, $CDCl_3$) δ 154.57, 152.61, 139.31, 133.54, 133.00, 131.92, 131.05, 130.76, 129.90, 124.96 (q, J = 3.7 Hz), 124.09 (q, J = 270.0 Hz), 124.00, 113.72, 29.38.

^{19}F NMR (471 MHz, $CDCl_3$) δ -62.82 (s, 3F).

HRMS (ESI+) calcd for $C_{16}H_{12}F_3N_2O^+$ $(M+H)^+$, m/z 305.0897, found 305.0899.

3-(2,3-dichlorophenyl)-1-methylquinoxalin-2(1H)-one (3ad) [5]

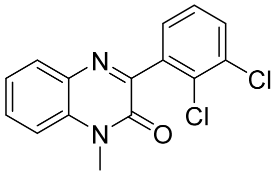

3ad

The title compound was prepared according to the typical procedure, as described above, in 88% yield, as yellow solid, 147-149 °C.

^{1}H NMR (500 MHz, $CDCl_3$) δ 7.94 (dd, J = 8.0, 1.5 Hz, 1H), 7.65 (ddd, J = 8.6, 7.4, 1.6 Hz, 1H), 7.52 (d, J = 2.0 Hz, 1H), 7.46 (d, J = 8.0 Hz, 1H), 7.43 – 7.39 (m, 2H), 7.37 (dd, J = 8.2, 2.0 Hz, 1H), 3.78 (s, 3H).

^{13}C NMR (125 MHz, $CDCl_3$) δ 155.29, 153.94, 135.89, 134.36, 133.81, 132.76, 131.51, 131.29, 130.71, 129.75, 127.18, 124.46, 124.00, 113.87, 29.47.

HRMS (ESI+) calcd for $C_{15}H_{11}Cl_2N_2O^+$ $(M+H)^+$, m/z 305.0243, found 305.0245.

3-(2,4-dichlorophenyl)-1-methylquinoxalin-2(1H)-one (3ae) [5]

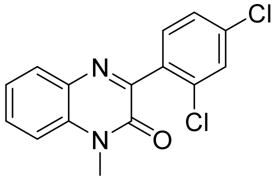

3ae

The title compound was prepared according to the typical procedure, as described above, in 84% yield, as yellow solid, 144-145 °C.

^{1}H NMR (500 MHz, $CDCl_3$) δ 7.94 (d, J = 7.2 Hz, 1H), 7.66 (t, J = 7.5 Hz, 1H), 7.50 (d, J = 2.4 Hz, 1H), 7.43 – 7.37 (m, 4H), 3.79 (s, 3H).

^{13}C NMR (125 MHz, $CDCl_3$) δ 155.07, 153.80, 137.20, 133.85, 132.81, 132.70, 131.76, 131.41, 130.92, 130.76, 130.57, 130.51, 124.05, 113.90, 29.47.

HRMS (ESI+) calcd for $C_{15}H_{11}Cl_2N_2O^+$ $(M+H)^+$, m/z 305.0243, found 305.0245.

1-methyl-3-(perfluorophenyl)quinoxalin-2(1H)-one (3af) [6]

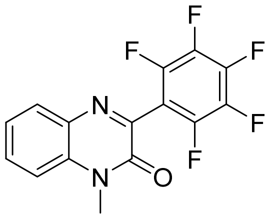

3af

The title compound was prepared according to the typical procedure, as described above, in 62% yield, as yellow solid, 120-122 °C.

^{1}H NMR (500 MHz, $CDCl_3$) δ 8.38 – 8.26 (m, 1H), 8.00 (s, 0.5H), 7.74 – 7.66 (m, 1H), 7.53 – 7.47 (m, 1H), 7.35 (s, 0.5H), 3.76 – 3.74 (m, 3H).

^{13}C NMR (125 MHz, $CDCl_3$) δ 154.90, 151.40 (d, J = 56.2 Hz), 150.34 (d, J = 46.2 Hz), 143.36, 133.84, 133.33 (d, J = 6.2 Hz), 132.48, 132.30, 130.79, 126.14, 115.60, 114.35, 28.91.

^{19}F NMR (471 MHz, $CDCl_3$) δ -22.47, -44.73, -49.80, -115.19, -152.50.

HRMS (ESI+) calcd for $C_{15}H_8F_5N_2O^+$ $(M+H)^+$, m/z 327.0552, found 327.0554.

1-methyl-3-(naphthalen-2-yl)quinoxalin-2(1H)-one (3ag) [6]

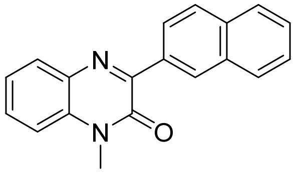

3ag

The title compound was prepared according to the typical procedure, as described above, in 59% yield, as yellow solid, 191-193 °C.

^{1}H NMR (500 MHz, $CDCl_3$) δ 9.08 (s, 1H), 8.41 (dd, J = 8.7, 1.8 Hz, 1H), 8.01 – 7.98 (m, 2H), 7.93 (d, J = 8.7 Hz, 1H), 7.87 (d, J = 7.3 Hz, 1H), 7.59 (ddd, J = 8.5, 7.3, 1.5 Hz, 1H), 7.55 – 7.50 (m, 2H), 7.40 (ddd, J = 8.2, 7.3, 1.2 Hz, 1H), 7.36 (dd, J = 8.4, 1.2 Hz, 1H), 3.82 (s, 3H).

^{13}C NMR (125 MHz, $CDCl_3$) δ 154.93, 153.53, 134.29, 133.39, 133.33, 133.22, 132.98, 130.58, 130.52, 130.36, 129.42, 127.61, 127.56, 127.24, 126.15, 126.12, 123.81, 113.61, 29.35.

HRMS (ESI+) calcd for $C_{19}H_{15}N_2O^+$ $(M+H)^+$, m/z 287.1179, found 287.1181.

3-cyclopentyl-1-methylquinoxalin-2(1H)-one (3ah) [7]

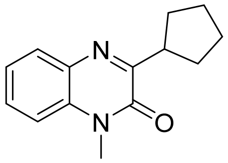

3ah

The title compound was prepared according to the typical procedure, as described above, in 95% yield, as yellow solid, 110-111 °C.

^{1}H NMR (500 MHz, $CDCl_3$) δ 7.82 (d, J = 8.5 Hz, 1H), 7.50 (t, J = 7.1 Hz, 1H), 7.32 (t, J = 7.1 Hz, 1H), 7.28 (d, J = 8.3 Hz, 1H), 3.75 – 3.72 (m, 1H), 3.70 (s, 3H), 2.09 – 2.03 (m, 2H), 1.96 – 1.89 (m, 2H), 1.85 – 1.79 (m, 2H), 1.75 – 1.69 (m, 2H).

^{13}C NMR (126 MHz, $CDCl_3$) δ 163.74, 155.01, 132.97, 132.74, 129.76, 129.31, 123.38, 113.43, 42.73, 30.84, 29.03, 25.94.

HRMS (ESI+) calcd for $C_{14}H_{17}N_2O^+$ $(M+H)^+$, m/z 229.1336, found 229.1338.

3-cyclohexyl-1-methylquinoxalin-2(1H)-one (3ai) [7]

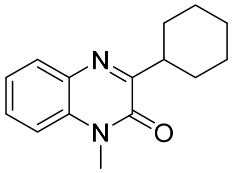

3ai

The title compound was prepared according to the typical procedure, as described above, in 94% yield, as yellow solid, 115-117 °C

^{1}H NMR (500 MHz, $CDCl_3$) δ 7.84 (d, J = 6.9 Hz, 1H), 7.50 (t, J = 7.4 Hz, 1H), 7.32 (t, J = 7.3 Hz, 1H), 7.28 (d, J = 8.6 Hz, 1H), 3.70 (s, 3H), 3.37 – 3.31 (m, 1H), 1.97 – 1.94 (m, 2H), 1.89 – 1.85 (m, 2H), 1.79 – 1.74 (m, 1H), 1.61 – 1.53 (m, 2H), 1.51 – 1.43 (m, 2H), 1.34 – 1.29 (m, 1H).

^{13}C NMR (125 MHz, $CDCl_3$) δ 164.30, 154.57, 132.92, 132.88, 129.79, 129.38, 123.39, 113.46, 40.79, 30.54, 29.06, 26.33, 26.17.

HRMS (ESI+) calcd for $C_{15}H_{19}N_2O^+$ $(M+H)^+$, m/z 243.1492, found 243.1494.

3-(2-hydroxyethyl)-1-methylquinoxalin-2(1H)-one (3aj) [8]

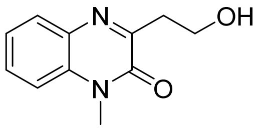

3aj

The title compound was prepared according to the typical procedure, as described above, in 89% yield, as yellow oil.

^{1}H NMR (500 MHz, $CDCl_3$) δ 7.83 (dd, J = 7.9, 1.5 Hz, 1H), 7.56 (ddd, J = 8.5, 7.3, 1.5 Hz, 1H), 7.36 (ddd, J = 8.2, 7.3, 1.2 Hz, 1H), 7.33 (dd, J = 8.4, 1.2 Hz, 1H), 4.11 (t, J = 5.4 Hz, 2H), 3.72 (s, 3H), 3.20 (t, J = 5.4 Hz, 2H).

^{13}C NMR (125 MHz, $CDCl_3$) δ 159.88, 155.21, 133.02, 132.28, 130.12, 129.67, 123.86, 113.75, 60.04, 35.96, 29.22.

HRMS (ESI+) calcd for $C_{11}H_{13}N_2O_2^+$ $(M+H)^+$, m/z 205.0972, found 205.0974.

2-phenylquinoline (5a) [9]

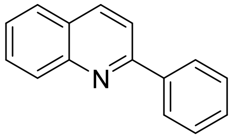

5a

The title compound was prepared according to the typical procedure, as described above, in 78% yield, as yellow solid, 82-84 °C.

^{1}H NMR (500 MHz, $CDCl_3$) δ 8.23 (d, J = 8.6 Hz, 1H), 8.19 – 8.16 (m, 3H), 7.88 (d, J = 8.6 Hz, 1H), 7.83 (dd, J = 8.2, 1.4 Hz, 1H), 7.73 (ddd, J = 8.4, 6.8, 1.5 Hz, 1H), 7.53 (td, J = 8.2, 7.6, 1.5 Hz, 3H), 7.47 (t, J = 7.3 Hz, 1H).

^{13}C NMR (125 MHz, $CDCl_3$) δ 157.39, 148.25, 139.66, 136.85, 129.71, 129.36, 128.87, 127.61, 127.21, 126.32, 124.91, 119.05, 114.31.

HRMS (ESI+) calcd for $C_{15}H_{12}N^{+}$ $(M+H)^{+}$, m/z 206.0965, found 206.0967.

3-methyl-2-phenylquinoline (5b) [9]

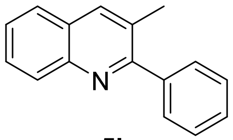

5b

The title compound was prepared according to the typical procedure, as described above, in 80% yield, as yellow solid, 51-52 °C.

^{1}H NMR (500 MHz, $CDCl_3$) δ 8.13 (d, J = 8.5 Hz, 1H), 8.02 (s, 1H), 7.78 (d, J = 8.1 Hz, 1H), 7.66 (t, J = 7.6 Hz, 1H), 7.59 (d, J = 7.3 Hz, 2H), 7.50 (dt, J = 14.4, 7.5 Hz, 3H), 7.43 (t, J = 7.3 Hz, 1H), 2.46 (s, 3H).

^{13}C NMR (125 MHz, $CDCl_3$) δ 160.56, 146.63, 140.88, 136.77, 129.31, 129.24, 128.87, 128.77, 128.32, 128.21, 127.62, 126.72, 126.43, 20.64.

HRMS (ESI+) calcd for $C_{16}H_{14}N^{+}$ $(M+H)^{+}$, m/z 220.1121, found 220.1123.

4-methyl-2-phenylquinoline (5c) [9]

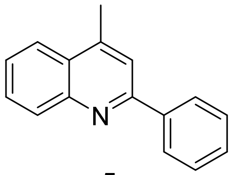

5c

The title compound was prepared according to the typical procedure, as described above, in 82% yield, as yellow solid, 49-50 °C.

^{1}H NMR (500 MHz, $CDCl_3$) δ 8.18 (d, J = 8.5 Hz, 1H), 8.16 – 8.14 (m, 2H), 8.01 (dd, J = 8.4, 1.4 Hz, 1H), 7.74 – 7.70 (m, 2H), 7.57 – 7.51 (m, 3H), 7.48 – 7.44 (m, 1H), 2.78 (s, 3H).

^{13}C NMR (125 MHz, $CDCl_3$)δ 157.10, 130.26, 129.38, 129.23, 128.80, 127.57, 127.27, 126.07, 123.63, 119.82, 19.06.

HRMS (ESI+) calcd for $C_{16}H_{14}N^{+}$ $(M+H)^{+}$, m/z 220.1121, found 220.1123.

8-methyl-2-phenylquinoline (5d) [9]

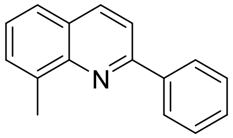

5d

The title compound was prepared according to the typical procedure, as described above, in 80% yield, as yellow solid, 51-53 °C.

^{1}H NMR (500 MHz, $CDCl_3$) δ 8.27 (d, *J* = 1.5 Hz, 1H), 8.26 (t, *J* = 1.3 Hz, 1H), 8.19 (d, *J* = 8.5 Hz, 1H), 7.91 (d, *J* = 8.6 Hz, 1H), 7.66 (d, *J* = 8.0 Hz, 1H), 7.57 (dt, *J* = 7.0, 1.2 Hz, 1H), 7.55 – 7.51 (m, 2H), 7.47 – 7.44 (m, 1H), 7.41 (dd, *J* = 8.1, 7.0 Hz, 1H), 2.91 (s, 3H).

^{13}C NMR (125 MHz, $CDCl_3$) δ 155.55, 147.19, 139.88, 137.70, 136.95, 129.69, 129.24, 128.79, 127.48, 127.11, 126.04, 125.39, 118.22, 17.91.

HRMS (ESI+) calcd for $C_{16}H_{14}N^+$ $(M+H)^+$, m/z 220.1121, found 220.1123.

2-phenylbenzo[h]quinoline (5e) [9]

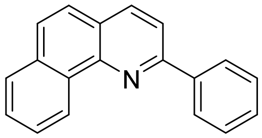

5e

The title compound was prepared according to the typical procedure, as described above, in 50% yield, as yellow solid, 137-138 °C.

^{1}H NMR (500 MHz, $CDCl_3$) δ 9.50 (d, *J* = 8.1 Hz, 1H), 8.36 – 8.34 (m, 2H), 8.24 (d, *J* = 8.3 Hz, 1H), 8.02 (dd, *J* = 8.4, 1.0 Hz, 1H), 7.92 (d, *J* = 7.8 Hz, 1H), 7.80 (d, *J* = 8.8 Hz, 1H), 7.76 (t, *J* = 7.5 Hz, 1H), 7.72 – 7.69 (m, 2H), 7.58 – 7.55 (m, 2H), 7.50 – 7.46 (m, 1H).

^{13}C NMR (125 MHz, $CDCl_3$) δ 155.54, 146.28, 139.78, 136.54, 133.90, 131.85, 129.22, 128.84, 128.16, 127.78, 127.47, 126.90, 125.18, 125.09, 124.76, 118.89.

HRMS (ESI+) calcd for $C_{19}H_{14}N^+$ $(M+H)^+$, m/z 256.1121, found 256.1123.

6-methoxy-2-phenylquinoline (5f) [9]

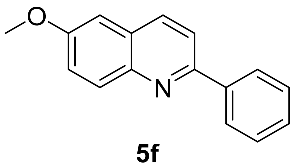

5f

The title compound was prepared according to the typical procedure, as described above, in 80% yield, as yellow solid, 57-58 °C.

^{1}H NMR (500 MHz, $CDCl_3$) δ 8.14 – 8.11 (m, 3H), 8.08 (d, J = 9.2 Hz, 1H), 7.84 (d, J = 8.6 Hz, 1H), 7.52 (dd, J = 8.3, 6.9 Hz, 2H), 7.46 – 7.42 (m, 1H), 7.39 (dd, J = 9.2, 2.8 Hz, 1H), 7.10 (d, J = 2.7 Hz, 1H), 3.95 (s, 3H).

^{13}C NMR (125 MHz, $CDCl_3$) δ 157.71, 155.08, 144.34, 139.76, 135.57, 131.17, 128.97, 128.82, 128.15, 127.31, 122.37, 119.28, 105.02, 55.57.

HRMS (ESI+) calcd for $C_{16}H_{14}NO^+$ $(M+H)^+$, m/z 236.1070, found 236.1072.

8-fluoro-2-phenylquinoline (5g) [10]

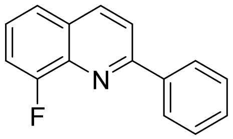

5g

The title compound was prepared according to the typical procedure, as described above, in 76% yield, as yellow solid, 62-64 °C.

^{1}H NMR (500 MHz, $CDCl_3$) δ 8.26 – 8.21 (m, 3H), 7.96 (d, J = 8.6 Hz, 1H), 7.62 (d, J = 7.7 Hz, 1H), 7.55 – 7.52 (m, 2H), 7.49 – 7.40 (m, 3H).

^{13}C NMR (125 MHz, $CDCl_3$) δ 159.33, 157.36 (d, J = 18.7 Hz), 139.18, 138.52, 136.55 (d, J = 1.2 Hz), 129.67, 128.90, 127.69, 126.01 (d, J = 10.0 Hz), 123.10 (d, J = 5.0 Hz), 119.83, 113.90, 113.75.

^{19}F NMR (471 MHz, $CDCl_3$) δ -125.26.

HRMS (ESI+) calcd for $C_{15}H_{11}FN^+$ $(M+H)^+$, m/z 224.0871, found 224.0873.

3-bromo-2-phenylquinoline (5h) [10]

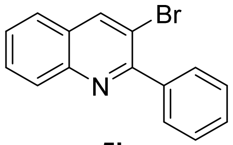

5h

The title compound was prepared according to the typical procedure, as described above, in 75% yield, as yellow solid, 95-97 °C.

^{1}H NMR (500 MHz, $CDCl_3$) δ 8.50 (s, 1H), 8.13 (d, J = 8.4 Hz, 1H), 7.79 (d, J = 8.2 Hz, 1H), 7.76 – 7.73 (m, 3H), 7.58 (ddd, J = 8.1, 6.8, 1.2 Hz, 1H), 7.52 – 7.45 (m, 3H).

^{13}C NMR (125 MHz, $CDCl_3$) δ 158.23, 146.61, 139.96, 139.93, 130.09, 129.59, 129.44, 128.89, 128.26, 128.05, 127.47, 126.48, 116.94.

HRMS (ESI+) calcd for $C_{15}H_{11}BrN^+$ $(M+H)^+$, m/z 284.0070, found 284.0072.

5-nitro-2-phenylquinoline (5i)

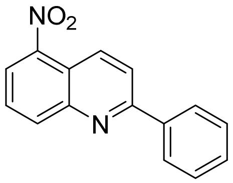

5i

The title compound was prepared according to the typical procedure, as described above, in 78% yield, as yellow solid, 130-131 °C.

^{1}H NMR (500 MHz, $CDCl_3$) δ 9.08 (d, J = 9.1 Hz, 1H), 8.48 (d, J = 8.4 Hz, 1H), 8.36 (dd, J = 7.7, 1.2 Hz, 1H), 8.23 – 8.21 (m, 2H), 8.13 (d, J = 9.1 Hz, 1H), 7.81 (t, J = 8.1 Hz, 1H), 7.59 – 7.52 (m, 3H).
^{13}C NMR (125 MHz, $CDCl_3$) δ 163.67, 150.48, 140.53, 138.27, 136.83, 132.75, 130.25, 129.07, 127.66, 124.21, 121.79, 120.13.

HRMS (ESI+) calcd for $C_{15}H_{11}N_2O_2^+$ $(M+H)^+$, m/z 251.0816, found 251.0818.

2-(p-tolyl)quinoline (5j) [10]

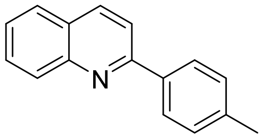

5j

The title compound was prepared according to the typical procedure, as described above, in 70% yield, as yellow solid, 59-61 °C.

^{1}H NMR (500 MHz, $CDCl_3$) δ 8.20 (d, J = 8.6 Hz, 1H), 8.16 (d, J = 8.5 Hz, 1H), 8.08 (s, 1H), 8.06 (s, 1H), 7.86 (d, J = 8.6 Hz, 1H), 7.81 (dd, J = 8.1, 1.4 Hz, 1H), 7.71 (ddd, J = 8.4, 6.8, 1.5 Hz, 1H), 7.51 (ddd, J = 8.0, 6.7, 1.2 Hz, 1H), 7.33 (d, J = 7.9 Hz, 2H), 2.43 (s, 3H).
^{13}C NMR (125 MHz, $CDCl_3$) δ 157.35, 148.28, 139.43, 136.86, 136.69, 129.66, 129.59, 127.46, 127.45, 127.12, 126.11, 118.88, 21.36.

HRMS (ESI+) calcd for $C_{16}H_{14}N^+$ $(M+H)^+$, m/z 220.1121, found 220.1123.

2-(4-isopropylphenyl)quinoline (5k) [10]

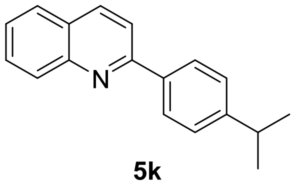

5k

The title compound was prepared according to the typical procedure, as described above, in 65% yield, as yellow solid, 101-102°C.

^{1}H NMR (500 MHz, $CDCl_3$) δ 8.20 (d, J = 8.6 Hz, 1H), 8.17 (d, J = 8.5 Hz, 1H), 8.10 (d, J = 2.0 Hz, 1H), 8.08 (d, J = 1.9 Hz, 1H), 7.86 (d, J = 8.6 Hz, 1H), 7.82 (dd, J = 8.1, 1.4 Hz, 1H), 7.71 (t, J = 7.6

Hz, 1H), 7.51 (ddd, J = 8.0, 6.9, 1.1 Hz, 1H), 7.40 (d, J = 1.9 Hz, 1H), 7.38 (d, J = 1.8 Hz, 1H), 2.99 (p, J = 6.9 Hz, 1H), 1.31 (d, J = 6.9 Hz, 6H).

^{13}C NMR (125 MHz, $CDCl_3$) δ 157.46, 150.36, 148.31, 137.33, 136.68, 129.69, 129.59, 127.59, 127.45, 127.10, 126.98, 126.10, 118.97, 34.02, 23.94.

HRMS (ESI+) calcd for $C_{18}H_{18}N^+$ $(M+H)^+$, m/z 248.1434, found 248.1436.

2-(naphthalen-2-yl)quinoline (5l) [11]

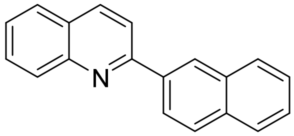

5l

The title compound was prepared according to the typical procedure, as described above, in 48% yield, as yellow solid, 123-124 °C.

^{1}H NMR (500 MHz, $CDCl_3$) δ 8.63 (d, J = 1.8 Hz, 1H), 8.38 (dd, J = 8.6, 1.8 Hz, 1H), 8.27 (d, J = 8.6 Hz, 1H), 8.23 (d, J = 8.5 Hz, 1H), 8.05 (d, J = 8.6 Hz, 1H), 8.02 – 7.99 (m, 2H), 7.91 – 7.89 (m, 1H), 7.86 (d, J = 8.1 Hz, 1H), 7.75 (ddd, J = 8.4, 6.8, 1.5 Hz, 1H), 7.56 – 7.52 (m, 3H).

^{13}C NMR (125 MHz, $CDCl_3$) δ 157.19, 136.87, 133.89, 133.53, 129.77, 129.74, 128.85, 128.60, 127.74, 127.51, 127.25, 127.19, 126.74, 126.38, 126.35, 125.08, 119.19.

HRMS (ESI+) calcd for $C_{19}H_{14}N^+$ $(M+H)^+$, m/z 256.1121, found 256.1123.

2-(4-methoxyphenyl)quinoline (5m) [11]

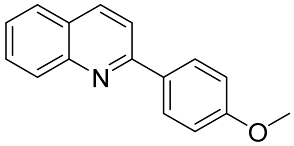

5m

The title compound was prepared according to the typical procedure, as described above, in 70% yield, as yellow solid, 60-61 °C.

^{1}H NMR (500 MHz, $CDCl_3$) δ 8.18 (d, J = 8.6 Hz, 1H), 8.15 (s, 1H), 8.14 (s, 1H), 7.84 (d, J = 8.6 Hz, 1H), 7.80 (dd, J = 8.1, 1.4 Hz, 1H), 7.71 (ddd, J = 8.4, 6.8, 1.5 Hz, 1H), 7.50 (ddd, J = 8.0, 6.8, 1.2 Hz, 1H), 7.06 (d, J = 2.2 Hz, 1H), 7.04 (d, J = 2.1 Hz, 1H), 3.89 (s, 3H).

^{13}C NMR (125 MHz, $CDCl_3$) δ 160.85, 156.92, 136.68, 132.23, 129.61, 129.51, 128.92, 127.44, 126.92, 125.94, 118.57, 114.25, 55.42.

HRMS (ESI+) calcd for $C_{16}H_{14}NO^+$ $(M+H)^+$, m/z 236.1070, found 236.1072.

2-(3-chlorophenyl)quinoline (5n) [11]

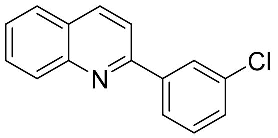

5n

The title compound was prepared according to the typical procedure, as described above, in 50% yield, as yellow solid, 62-64 °C.

^{1}H NMR (500 MHz, $CDCl_3$) δ 8.25 (d, *J* = 8.6 Hz, 1H), 8.21 – 8.17 (m, 2H), 8.04 (d, *J* = 6.6 Hz, 1H), 7.87 – 7.84 (m, 2H), 7.77 – 7.73 (m, 1H), 7.57 – 7.54 (m, 1H), 7.48 – 7.43 (m, 2H).

^{13}C NMR (125 MHz, $CDCl_3$) δ 155.78, 148.21, 141.43, 137.07, 134.98, 130.07, 129.92, 129.79, 129.33, 127.75, 127.50, 127.37, 126.68, 125.62, 118.73.

HRMS (ESI+) calcd for $C_{15}H_{11}ClN^+$ $(M+H)^+$, m/z 240.0575, found 240.0576.

2-(4-bromophenyl)quinoline (5o) [11]

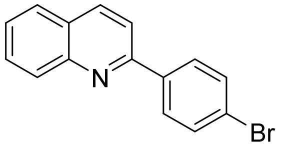

5o

The title compound was prepared according to the typical procedure, as described above, in 78% yield, as yellow solid, m.p. 83-84 °C.

^{1}H NMR (500 MHz, $CDCl_3$) δ 8.23 (d, *J* = 8.6 Hz, 1H), 8.16 (d, *J* = 8.5 Hz, 1H), 8.07 (d, *J* = 2.0 Hz, 1H), 8.06 (d, *J* = 2.0 Hz, 1H), 7.84 (dd, *J* = 8.8, 6.5 Hz, 2H), 7.74 (ddd, *J* = 8.4, 6.8, 1.5 Hz, 1H), 7.66 (d, *J* = 2.0 Hz, 1H), 7.65 (d, *J* = 1.9 Hz, 1H), 7.55 (ddd, *J* = 8.1, 6.8, 1.2 Hz, 1H).

^{13}C NMR (125 MHz, $CDCl_3$) δ 156.07, 137.04, 132.00, 129.90, 129.70, 129.12, 127.50, 127.27, 126.56, 123.97, 118.54.

HRMS (ESI+) calcd for $C_{15}H_{11}BrN^+$ $(M+H)^+$, m/z 284.0070, found 284.0072.

References:

1. M. Tian, S. Liu, X. Bu, J. Yu and X. Yang, *Chemistry – A European Journal*, 2019, **26**, 369-373.
2. J. Xu, H. Zhang, J. Zhao, Z. Ni, P. Zhang, B.-F. Shi and W. Li, *Organic Chemistry Frontiers*, 2020, **7**, 4031-4042.
3. H. Zhang, J. Xu, Y. Ouyang, X. Yue, C. Zhou, Z. Ni and W. Li, *Chinese Chemical Letters*, 2022, **33**, 2036-2040.
4. Y. Lv, P. Bao, H. Yue and W. Wei, *Tetrahedron Letters*, 2020, **61**, 152559.
5. S. Song, X. Shi, Y. Zhu, Q. Ren, P. Zhou, J. Zhou and J. Li, *The Journal of Organic Chemistry*, 2022, **87**, 4764-4776.
6. J. Sun, H. Yang and B. Zhang, *Green Chemistry*, 2022, **24**, 858-863.
7. L.-Y. Xie, S. Peng, L.-H. Yang, C. Peng, Y.-W. Lin, X. Yu, Z. Cao, Y.-Y. Peng and W.-M. He, *Green Chemistry*, 2021, **23**, 374-378.
8. H. Zhang, J. Xu, Y. Ouyang, X. Yue, C. Zhou, Z. Ni and W. Li, *Chinese Chemical Letters*, 2022, **33**, 2036-2040.
9. J. D. Galloway, D. N. Mai and R. D. Baxter, *Organic Letters*, 2017, **19**, 5772-5775.
10. J.-W. Yuan and L.-B. Qu, *Chinese Chemical Letters*, 2017, **28**, 981-985.
11. J.-W. Yuan, L.-R. Yang, P. Mao and L.-B. Qu, *Organic Chemistry Frontiers*, 2017, **4**, 545-554.

VI ^{1}H , ^{13}C and ^{19}F NMR of all the products

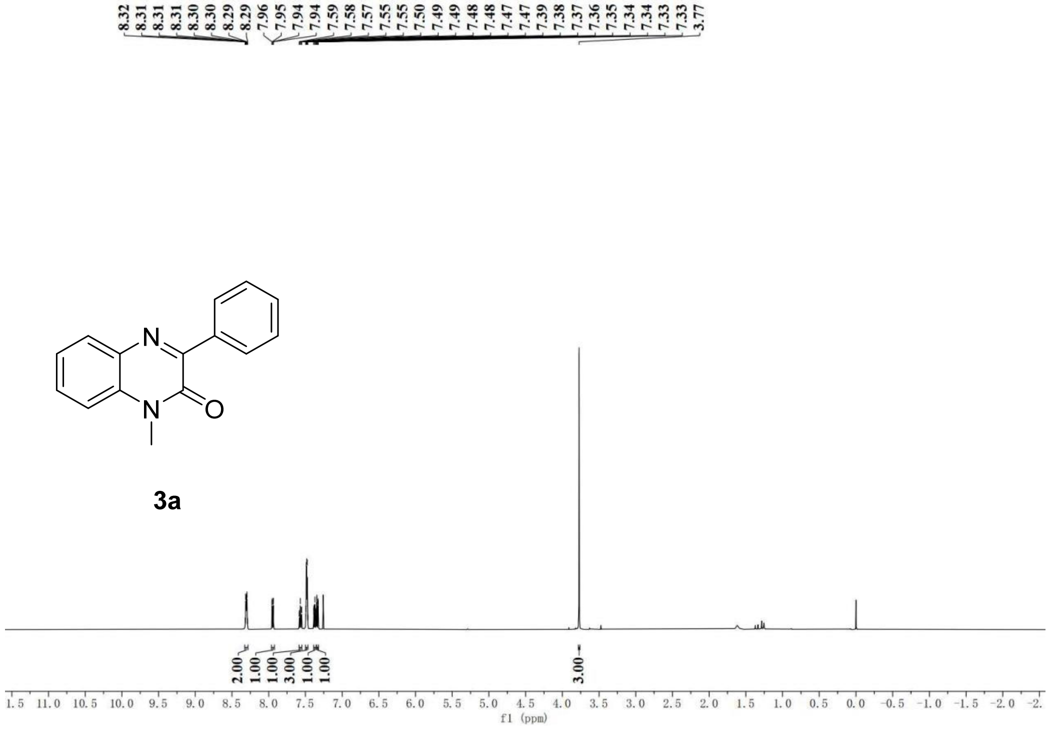

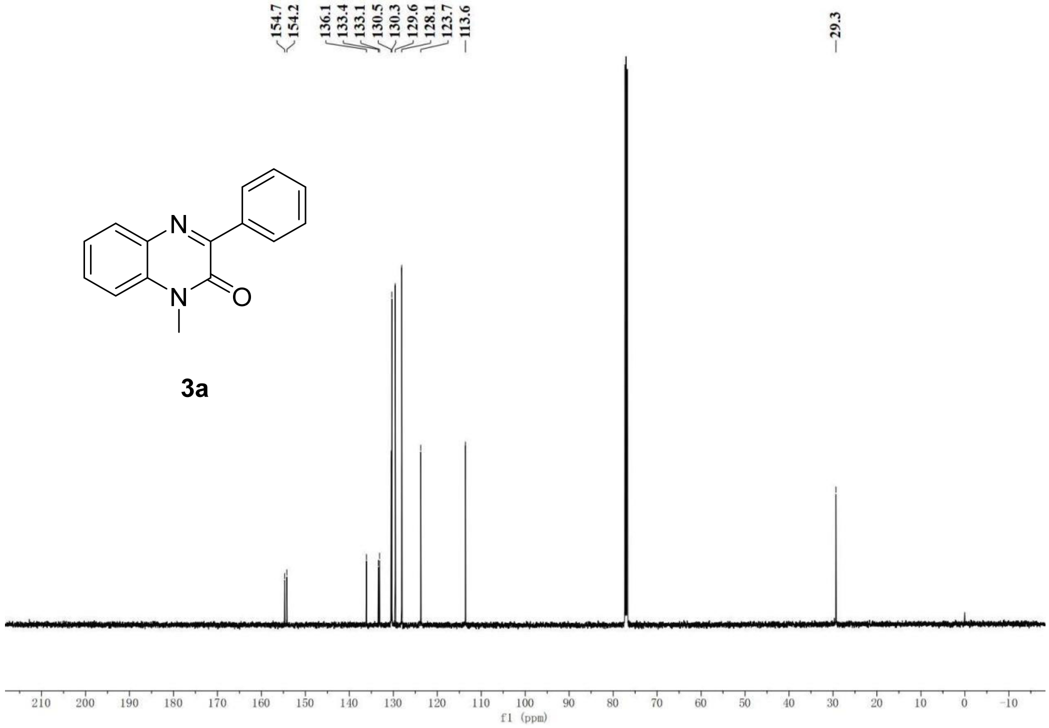

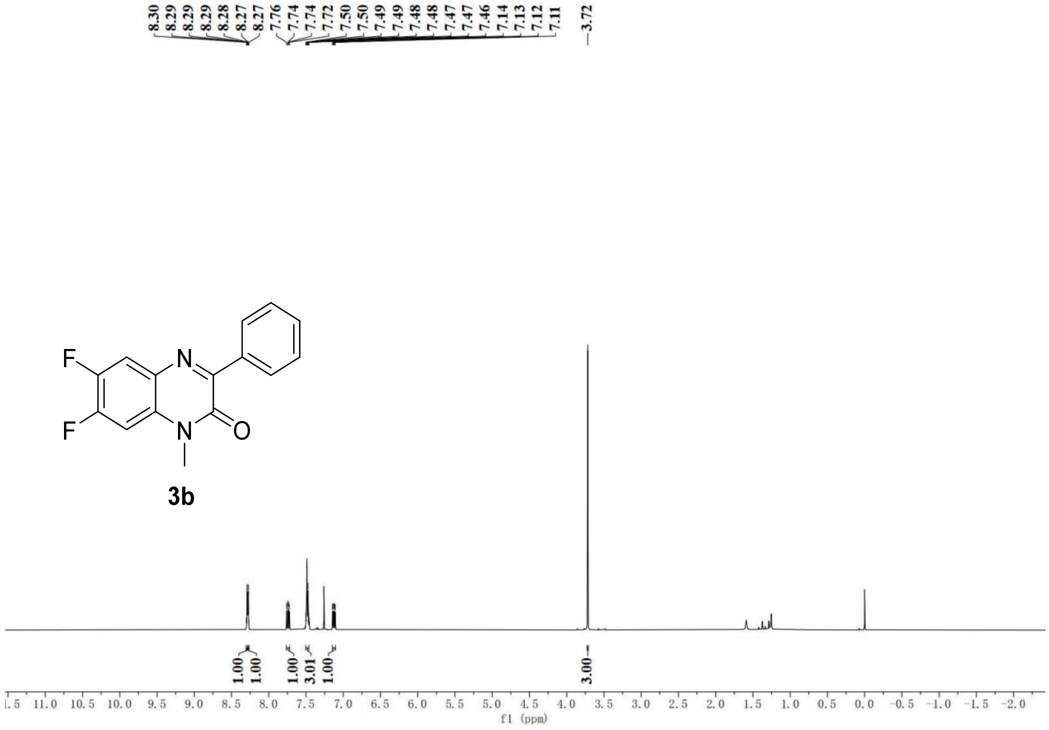

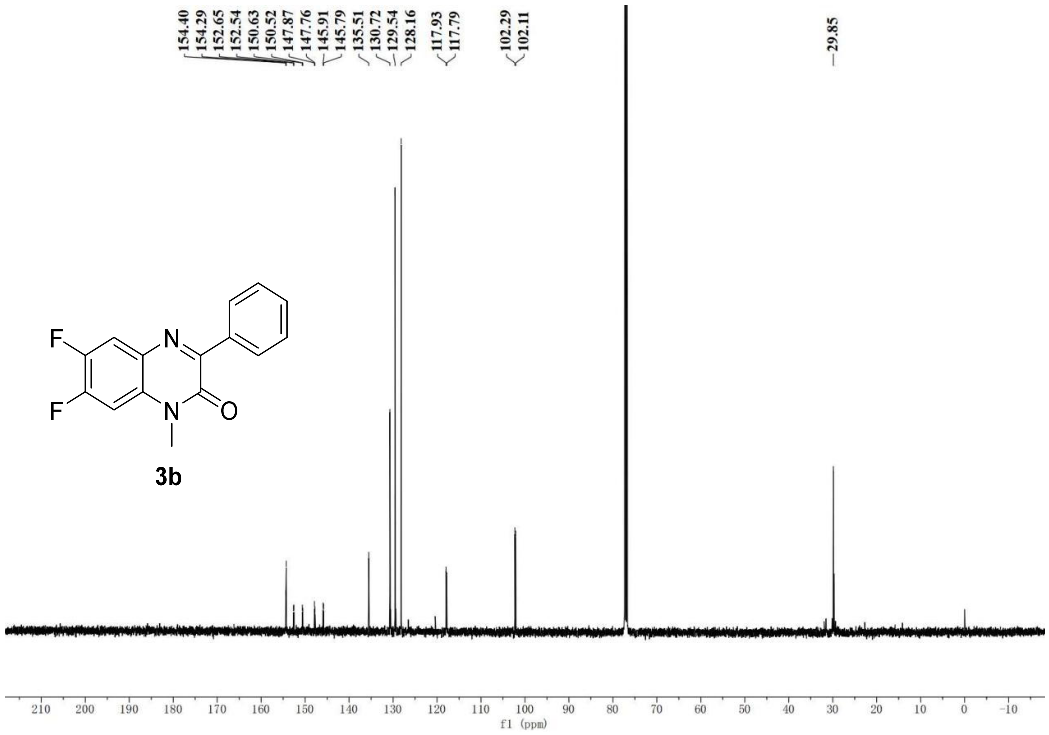

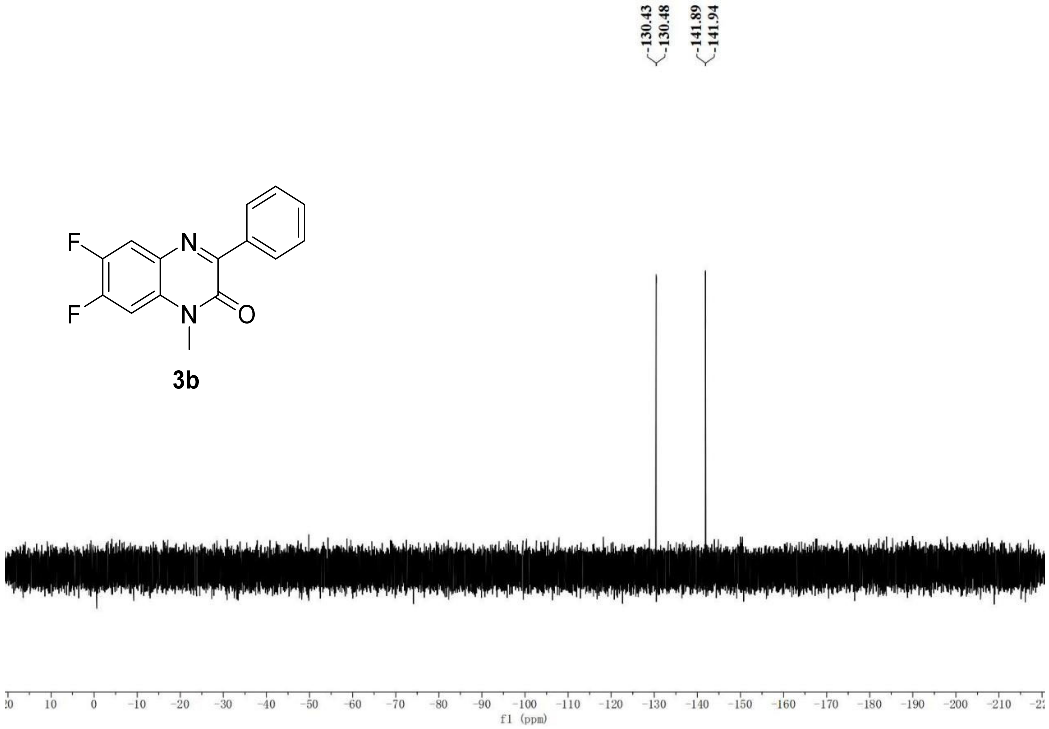

-130.43
-130.48
-141.89
-141.94
f1 (ppm)

3b

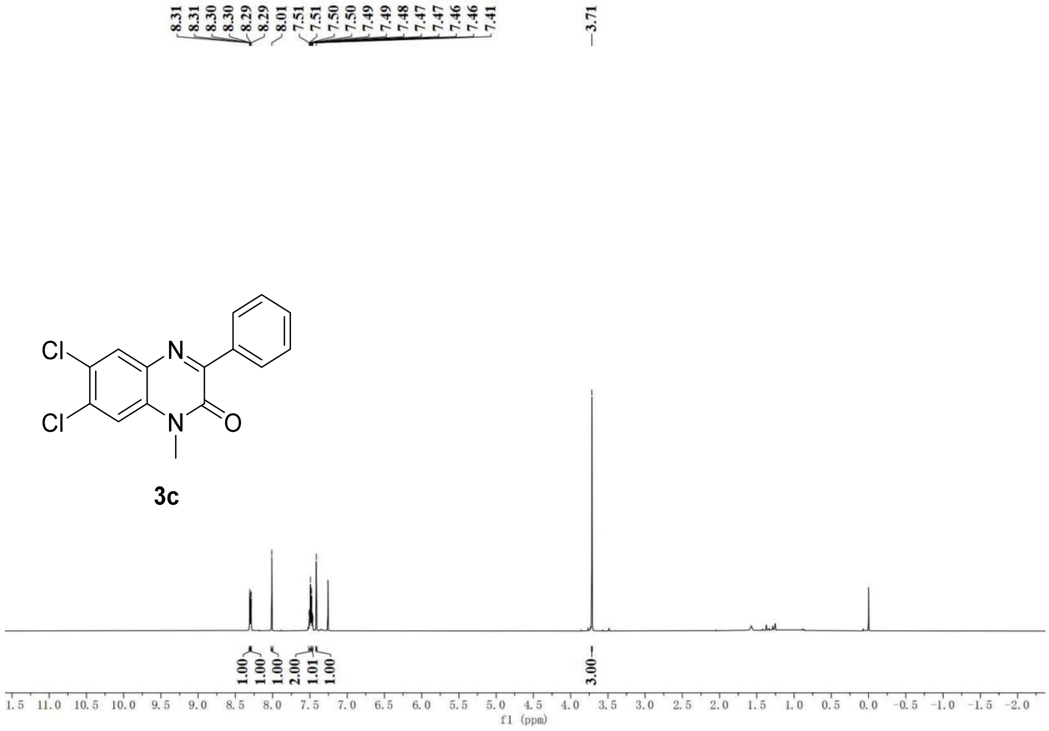

8.31
8.31
8.30
8.30
8.29
8.29
8.01
7.51
7.51
7.50
7.50
7.49
7.49
7.48
7.47
7.47
7.46
7.46
7.41
3.71
1.00
1.00
1.00
2.00
1.01
1.00
3.00
f1 (ppm)

3c

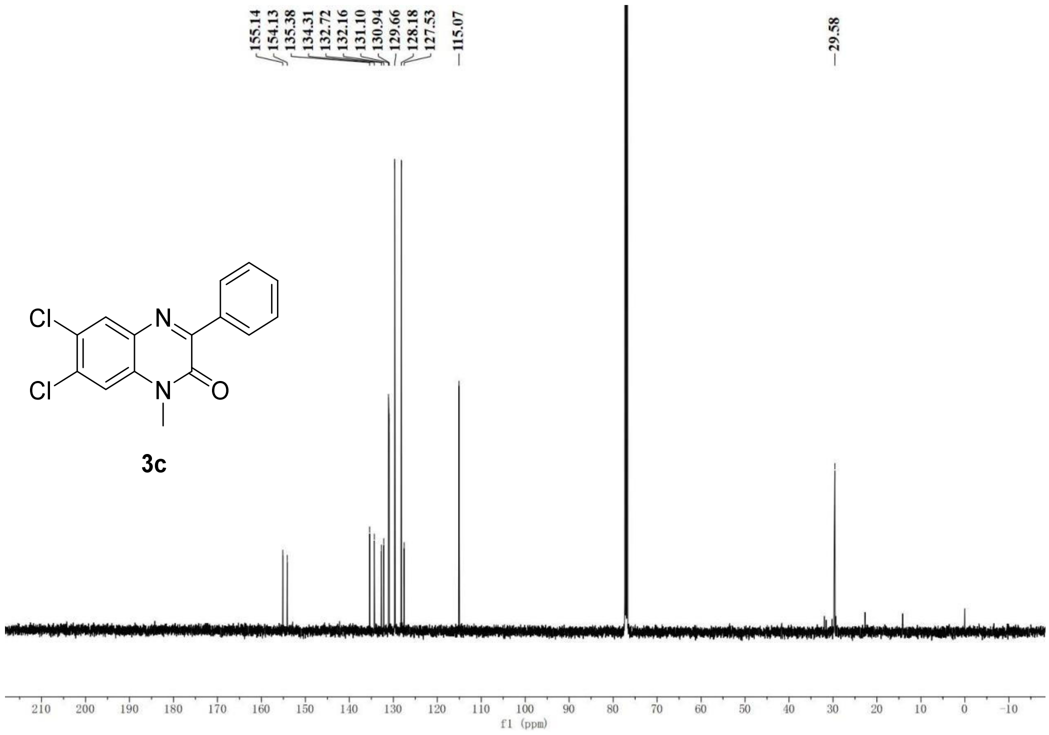
155.14
154.13
135.38
134.31
132.72
132.16
131.10
130.94
129.66
128.18
127.53
115.07
29.58
Cl
Cl
N
N
O
3c
f1 (ppm)

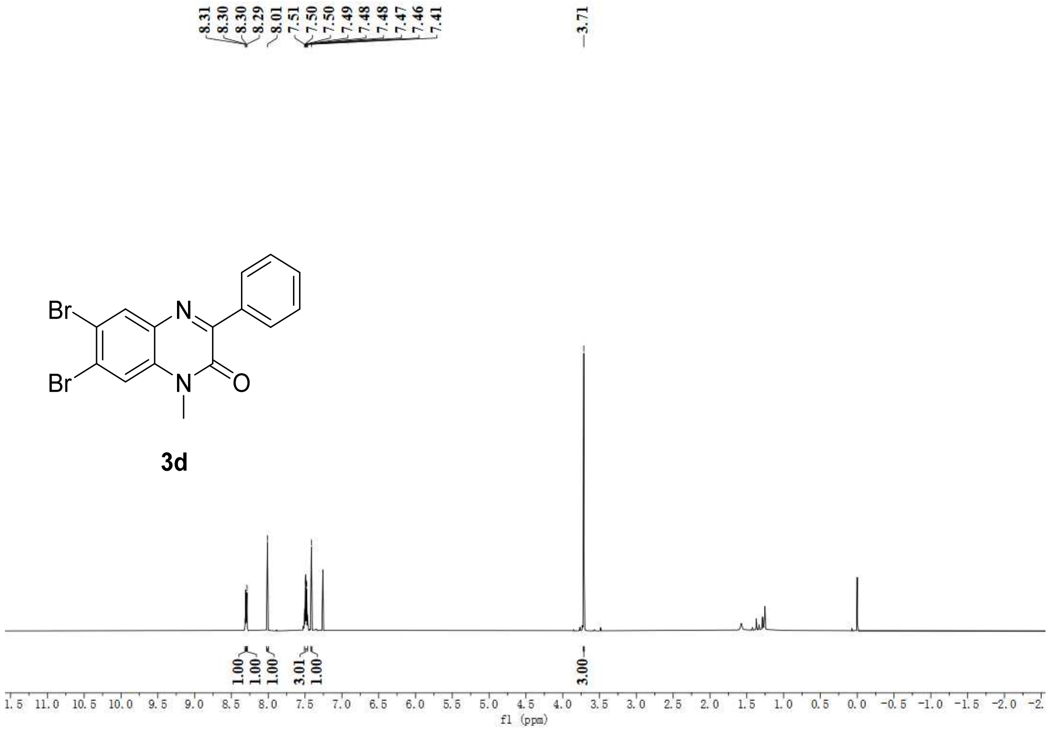
8.31
8.30
8.30
8.29
8.01
7.51
7.50
7.50
7.49
7.48
7.48
7.47
7.46
7.41
3.71
Br
Br
N
N
O
3d
1.00
1.00
1.00
3.01
1.00
3.00
f1 (ppm)

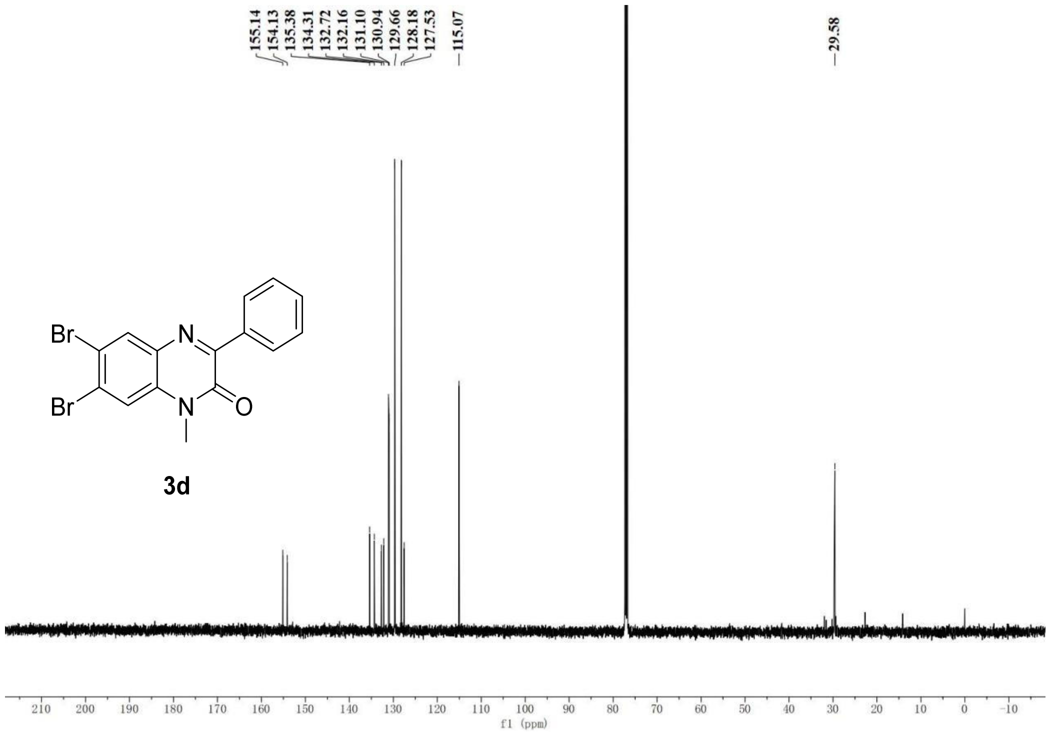
155.14
154.13
135.38
134.31
132.72
132.16
131.10
130.94
129.66
128.18
127.53
115.07
29.58
Br
Br
N
N
O
3d
210 200 190 180 170 160 150 140 130 120 110 100 90 80 70 60 50 40 30 20 10 0 -10
f1 (ppm)

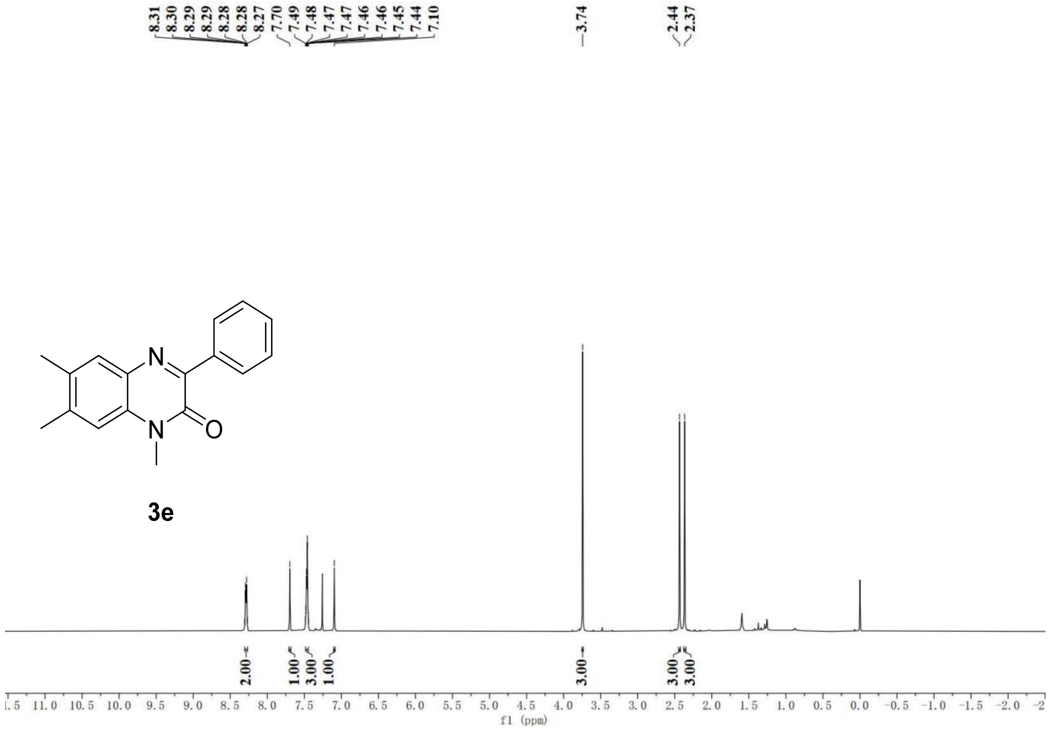
8.31
8.30
8.29
8.29
8.28
8.28
8.27
7.70
7.49
7.48
7.47
7.47
7.46
7.46
7.45
7.44
7.10
3.74
2.44
2.37
N
N
O
3e
2.00
1.00
3.00
1.00
3.00
3.00
3.00
11.0 10.5 10.0 9.5 9.0 8.5 8.0 7.5 7.0 6.5 6.0 5.5 5.0 4.5 4.0 3.5 3.0 2.5 2.0 1.5 1.0 0.5 0.0 -0.5 -1.0 -1.5 -2.0
f1 (ppm)

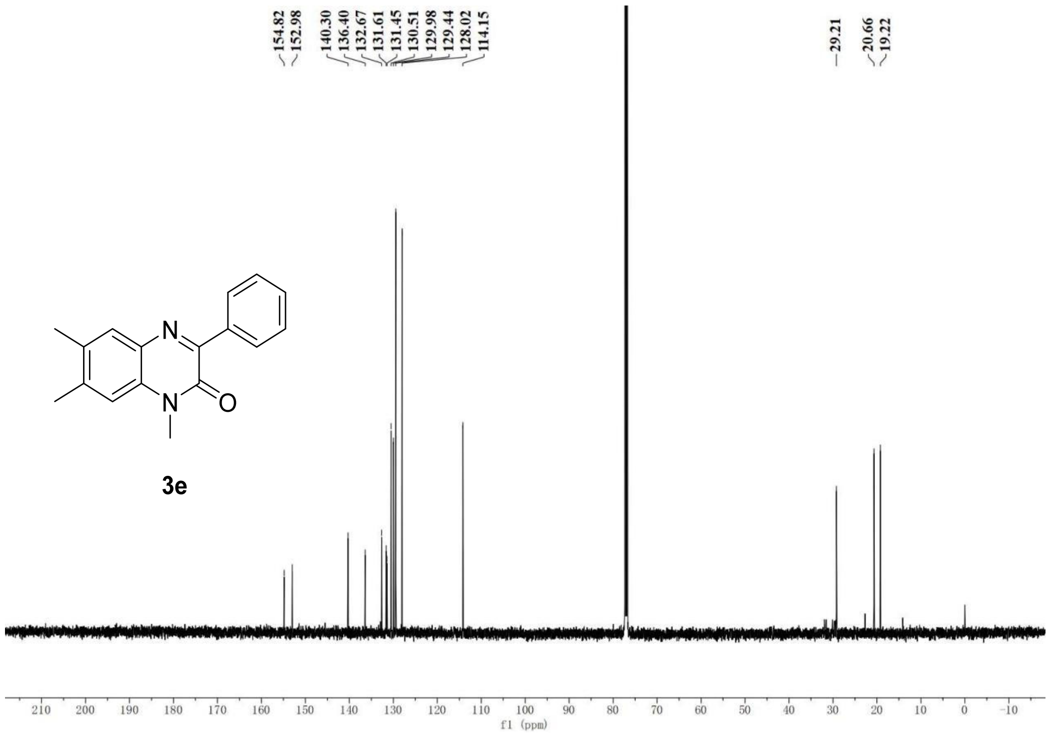
154.82
152.98
140.30
136.40
132.67
131.61
131.45
130.51
129.98
129.44
128.02
114.15
29.21
20.66
19.22
N
N
O
3e
210 200 190 180 170 160 150 140 130 120 110 100 90 80 70 60 50 40 30 20 10 0 -10
f1 (ppm)

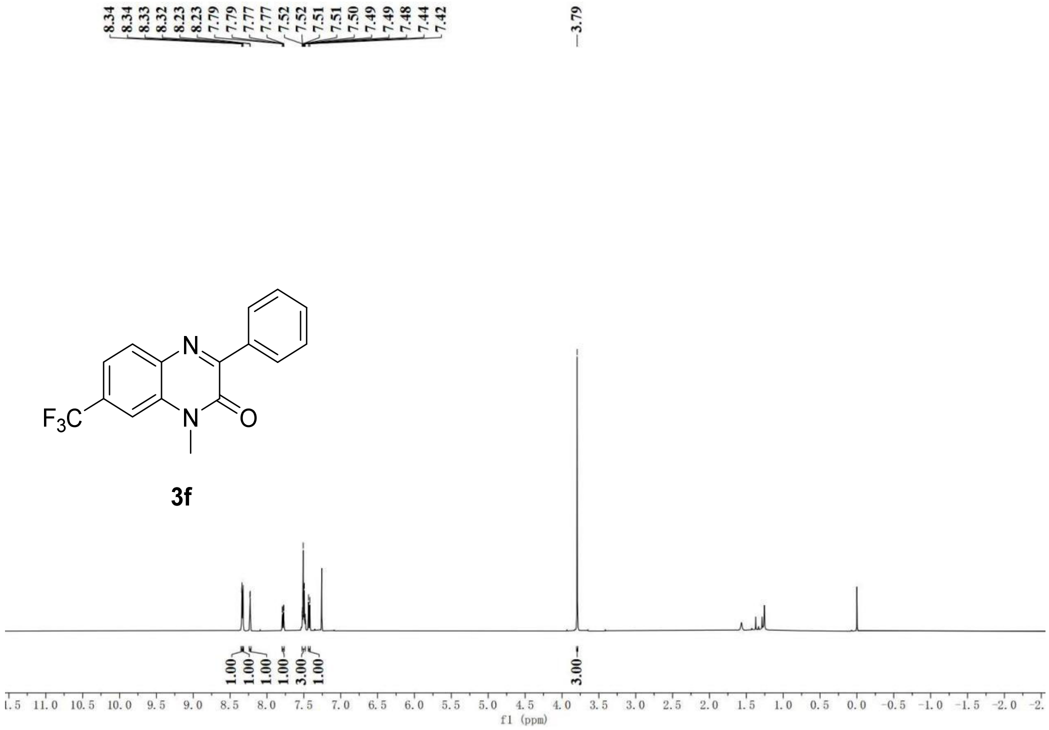
8.34
8.34
8.33
8.32
8.23
8.23
7.79
7.79
7.77
7.77
7.52
7.52
7.51
7.51
7.50
7.49
7.49
7.48
7.44
7.42
3.79
N
N
O
F_3C
3f
1.00
1.00
1.00
1.00
3.00
1.00
3.00
11.0 10.5 10.0 9.5 9.0 8.5 8.0 7.5 7.0 6.5 6.0 5.5 5.0 4.5 4.0 3.5 3.0 2.5 2.0 1.5 1.0 0.5 0.0 -0.5 -1.0 -1.5 -2.0
f1 (ppm)

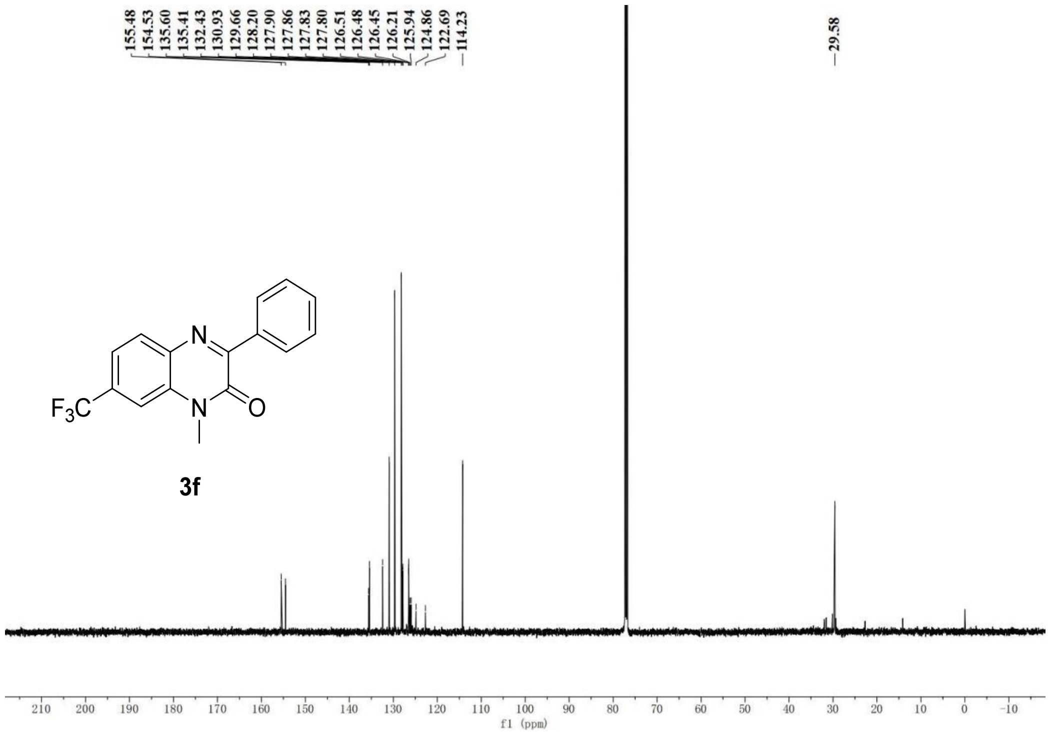
155.48
154.53
135.60
135.41
132.43
130.93
129.66
128.20
127.90
127.86
127.83
127.80
126.51
126.48
126.45
126.21
125.94
124.86
122.69
114.23
29.58
F3C
N
N
O
3f
f1 (ppm)

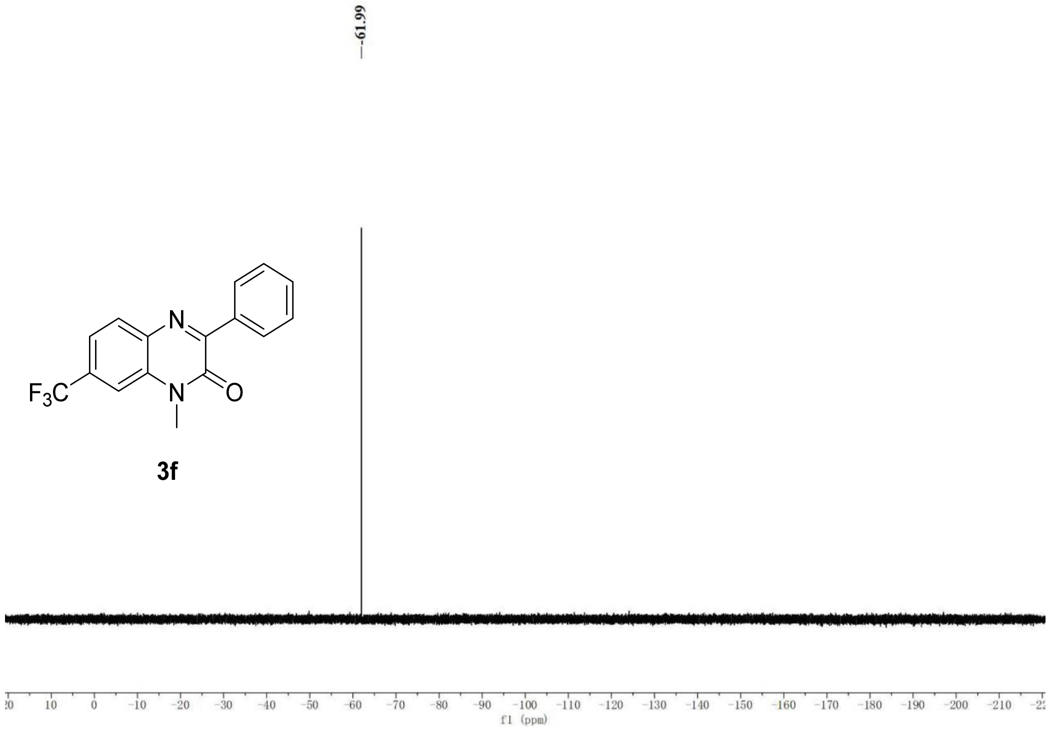
-61.99
F3C
N
N
O
3f
f1 (ppm)

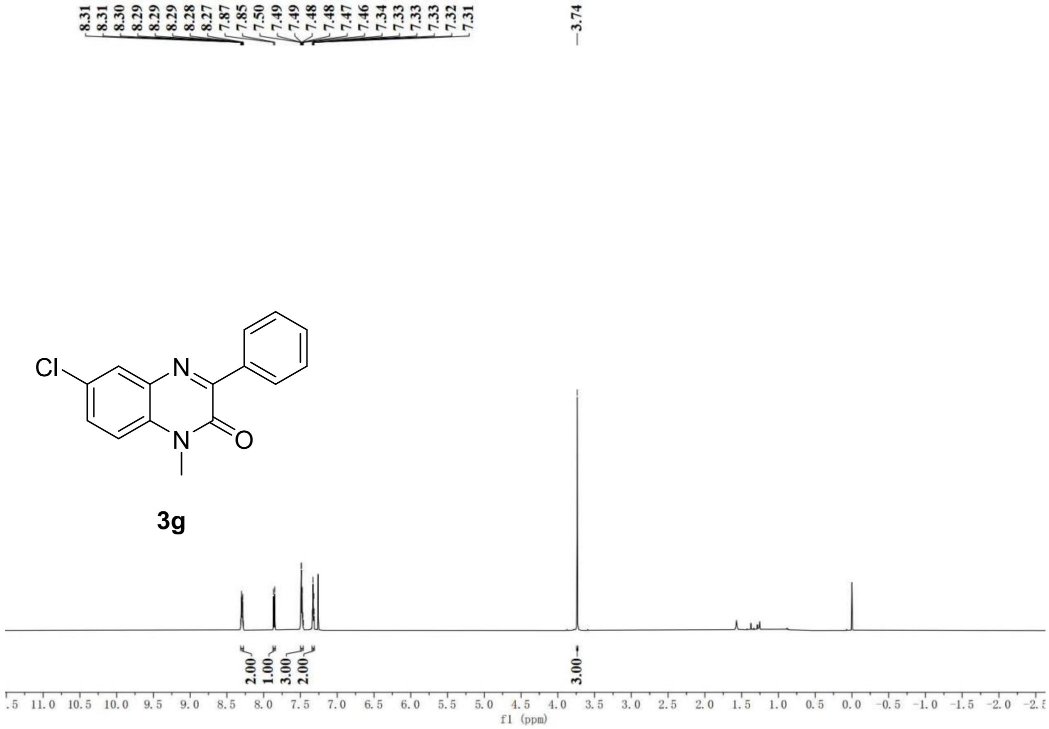
8.31
8.31
8.30
8.29
8.29
8.29
8.28
8.27
7.87
7.85
7.50
7.49
7.49
7.48
7.48
7.47
7.46
7.34
7.33
7.33
7.33
7.32
7.31
3.74
Cl
N
N
O
3g
2.00
1.00
3.00
2.00
3.00
f1 (ppm)

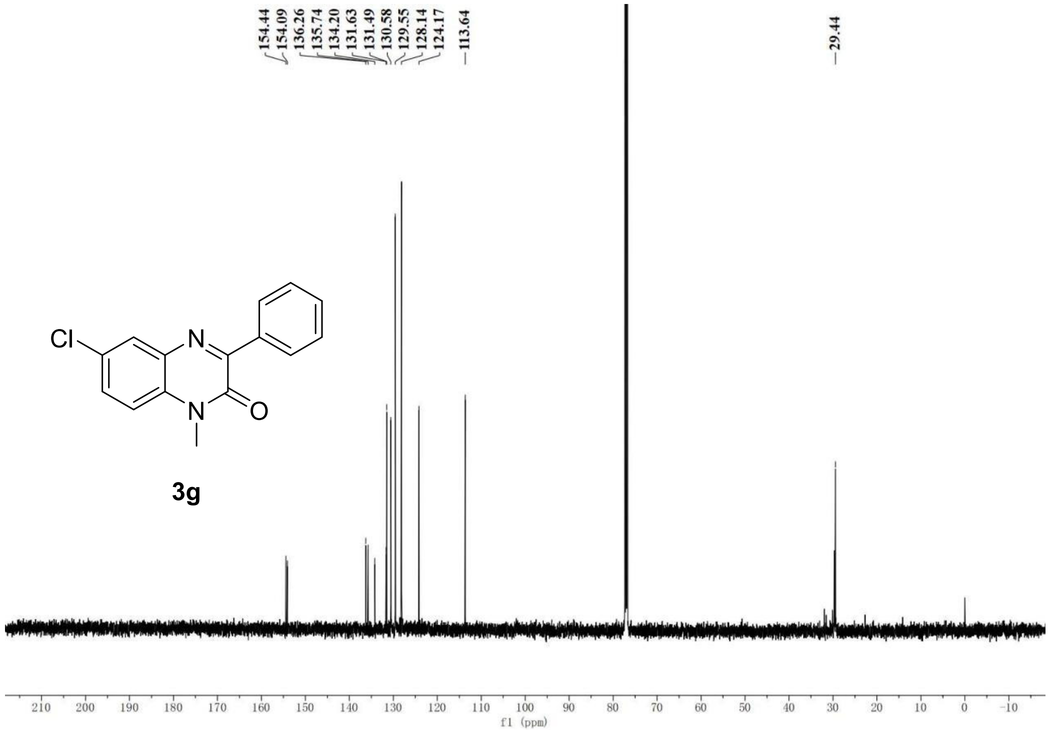
154.44
154.09
136.26
135.74
134.20
131.63
131.49
130.58
129.55
128.14
124.17
113.64
29.44
Cl
N
N
O
3g
f1 (ppm)

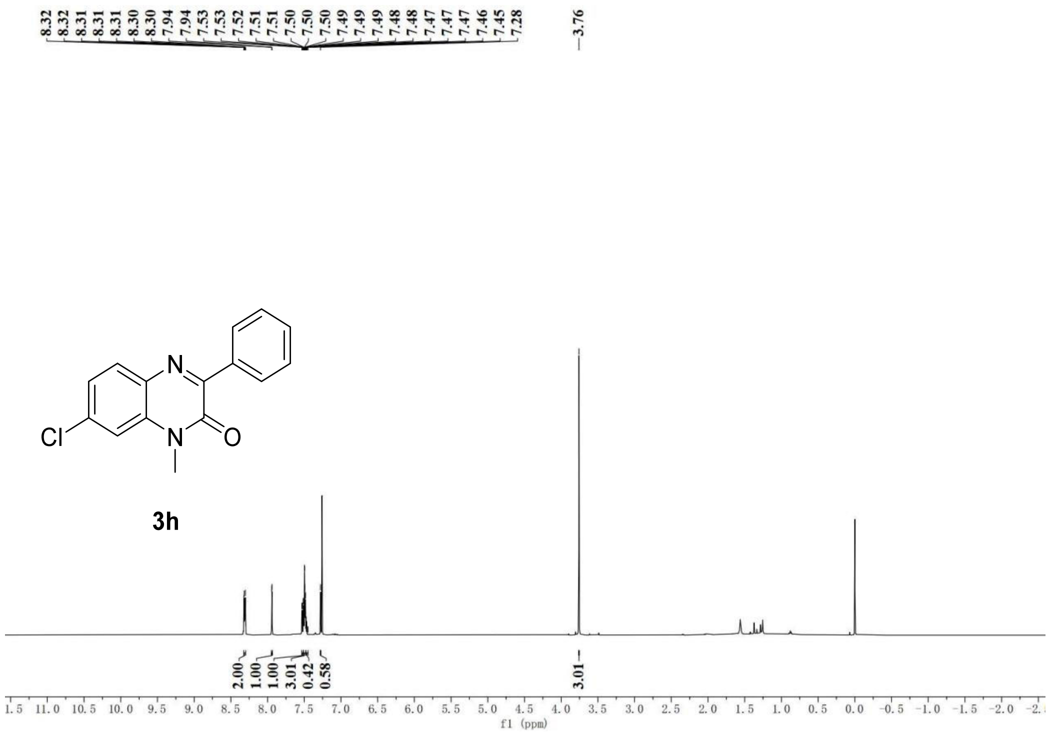

3h

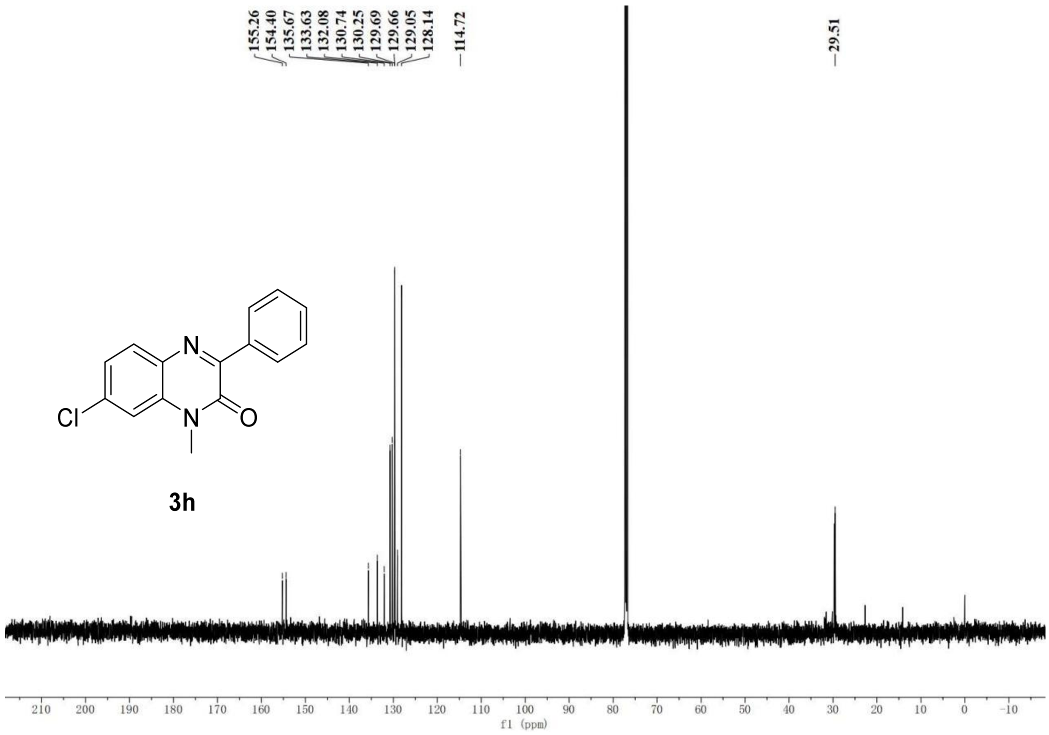

3h

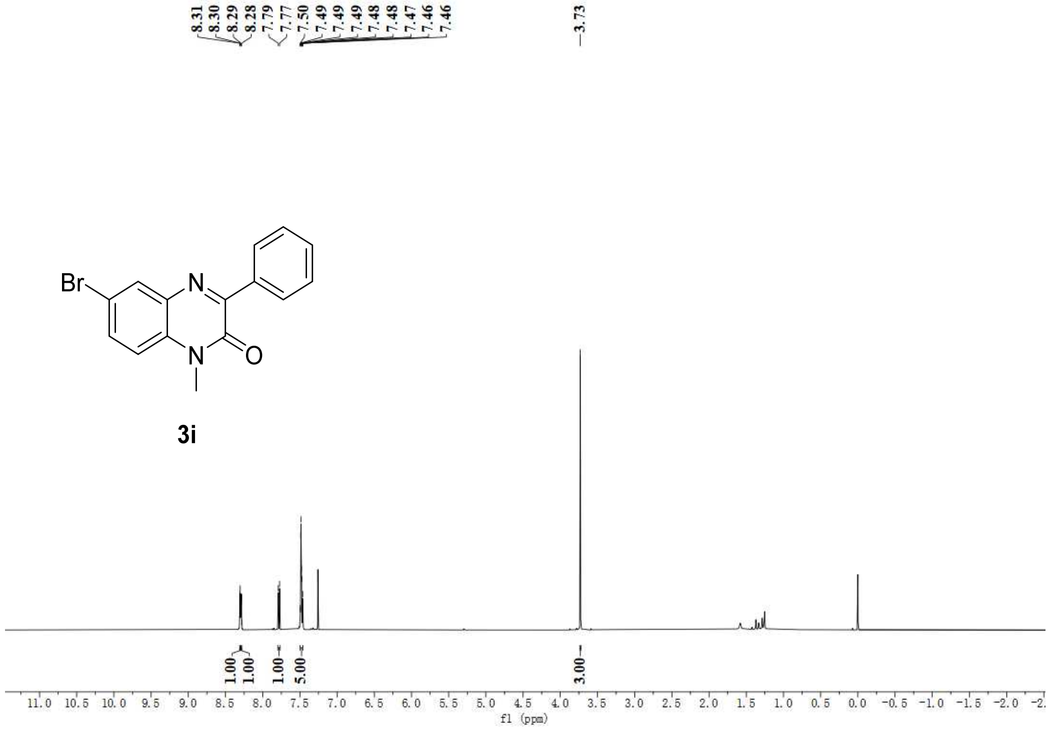
8.31
8.30
8.29
8.28
7.79
7.77
7.50
7.49
7.49
7.49
7.48
7.48
7.47
7.46
7.46
3.73
Br
N
N
O
3i
1.00
1.00
1.00
5.00
3.00
f1 (ppm)

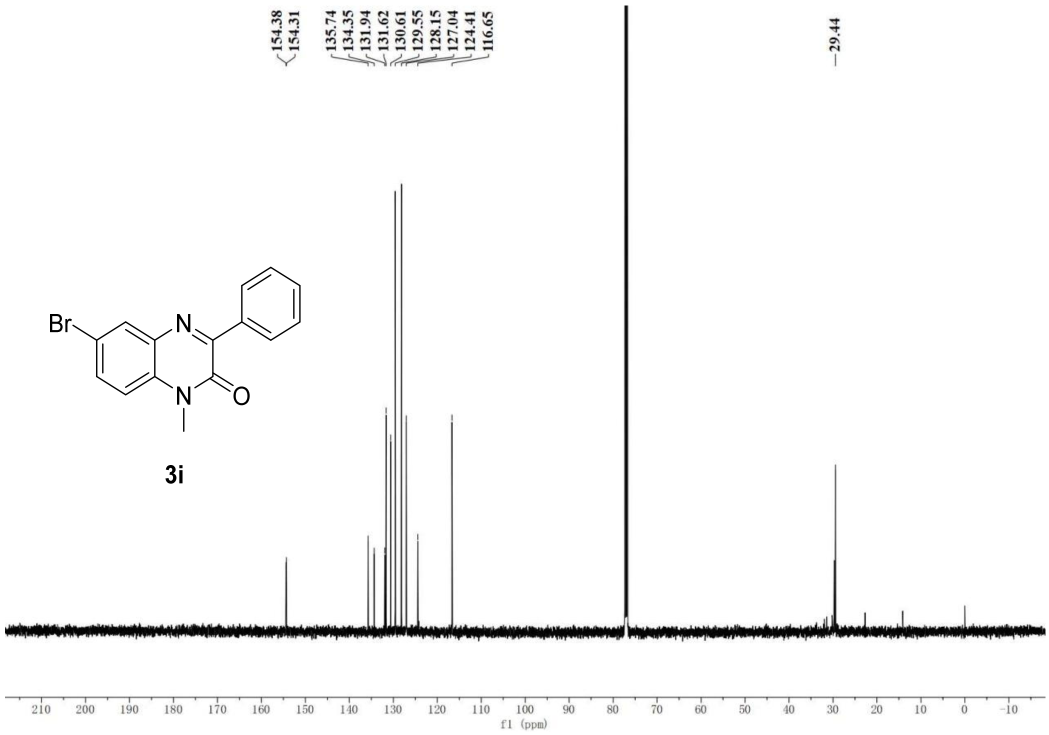
154.38
154.31
135.74
134.35
131.94
131.62
130.61
129.55
128.15
127.04
124.41
116.65
29.44
Br
N
N
O
3i
f1 (ppm)

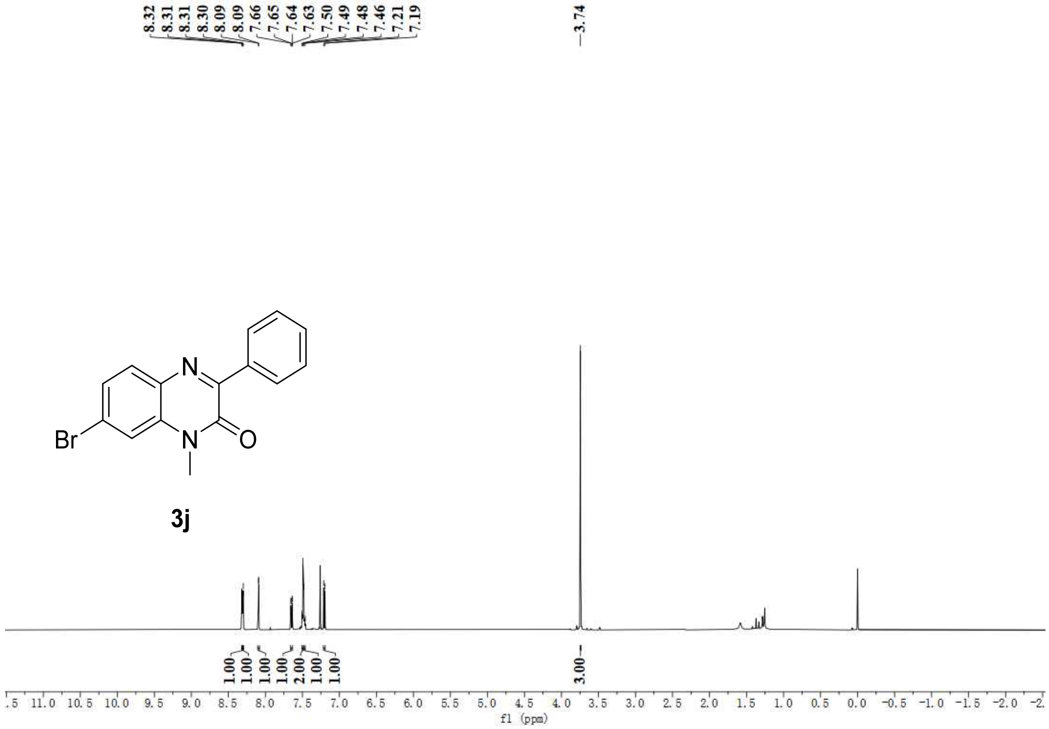
8.32
8.31
8.31
8.30
8.09
8.09
7.66
7.65
7.64
7.63
7.50
7.49
7.48
7.46
7.21
7.19
3.74
Br
N
N
O
3j
1.00
1.00
1.00
1.00
2.00
1.00
1.00
3.00
f1 (ppm)

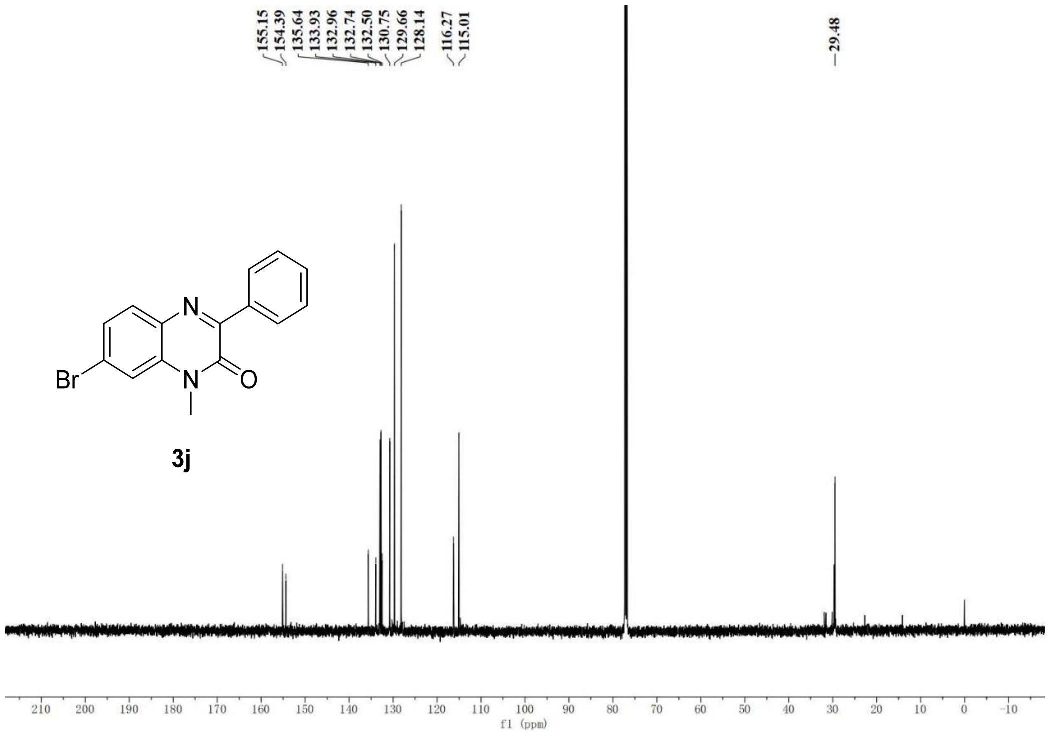
155.15
154.39
135.64
133.93
132.96
132.74
132.50
130.75
129.66
128.14
116.27
115.01
29.48
Br
N
N
O
3j
f1 (ppm)

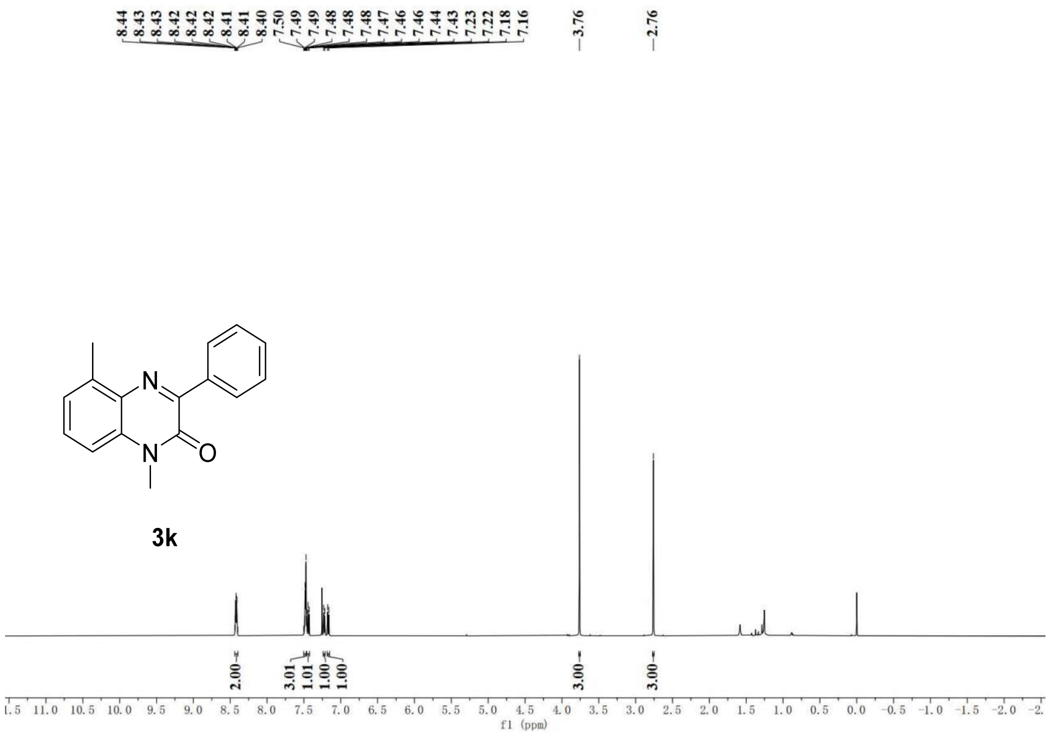

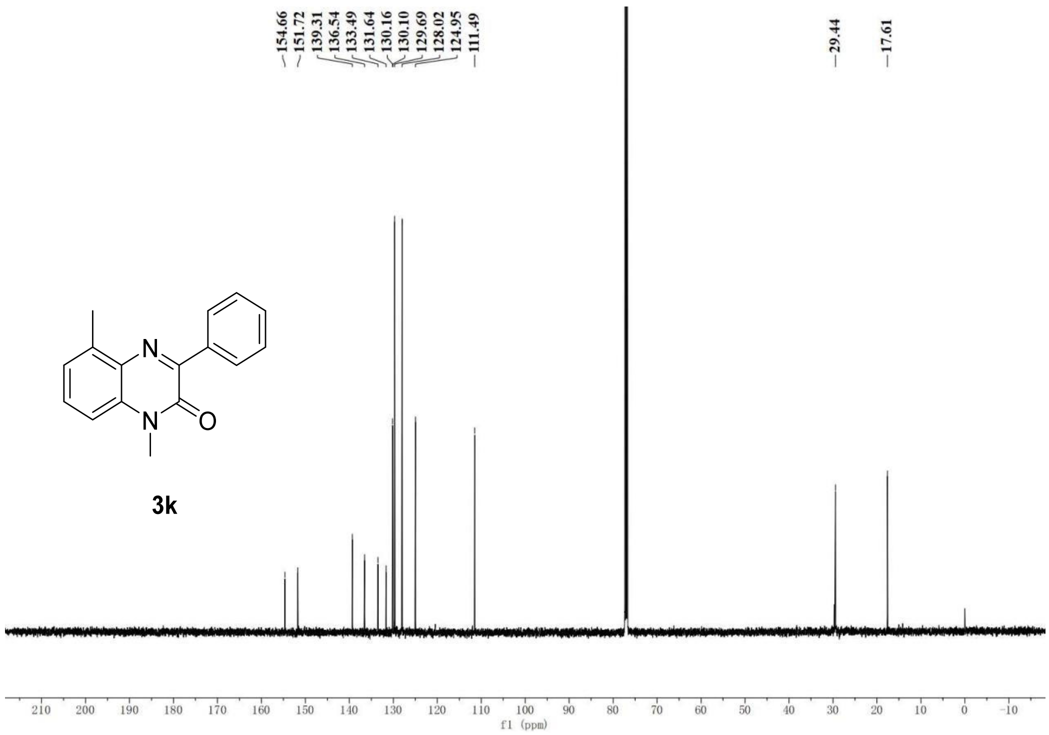

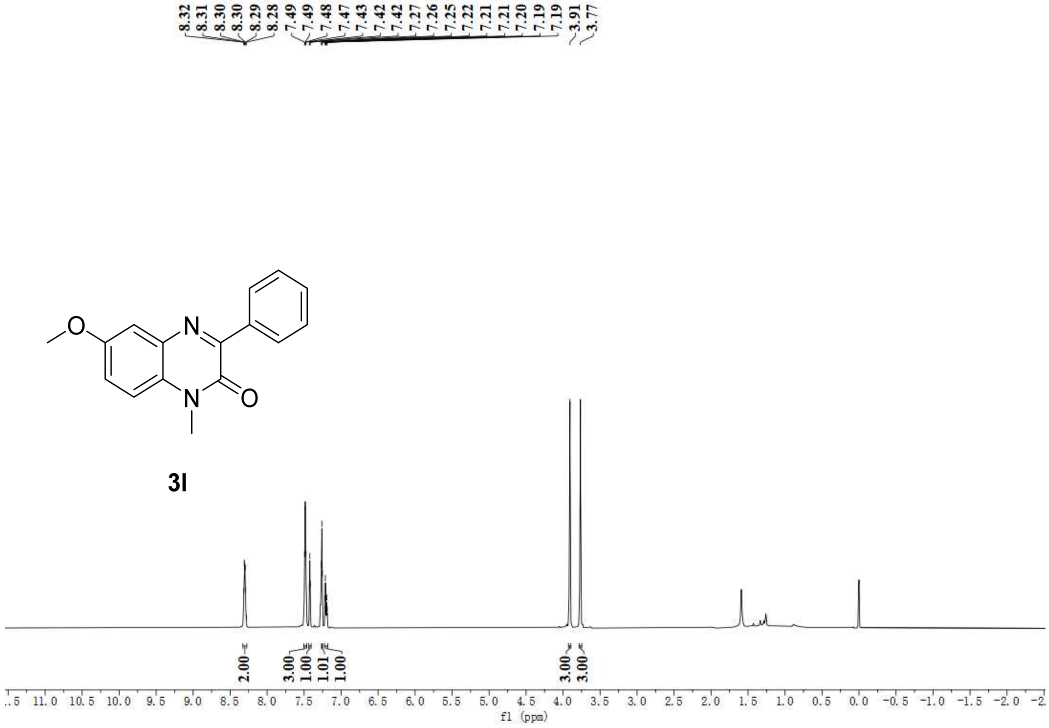
8.32 8.31 8.30 8.30 8.29 8.28 7.49 7.49 7.48 7.47 7.43 7.42 7.42 7.27 7.26 7.25 7.22 7.21 7.21 7.20 7.19 7.19 3.91 3.77
3l
2.00
3.00 1.00 1.01 1.00
3.00 3.00
f1 (ppm)

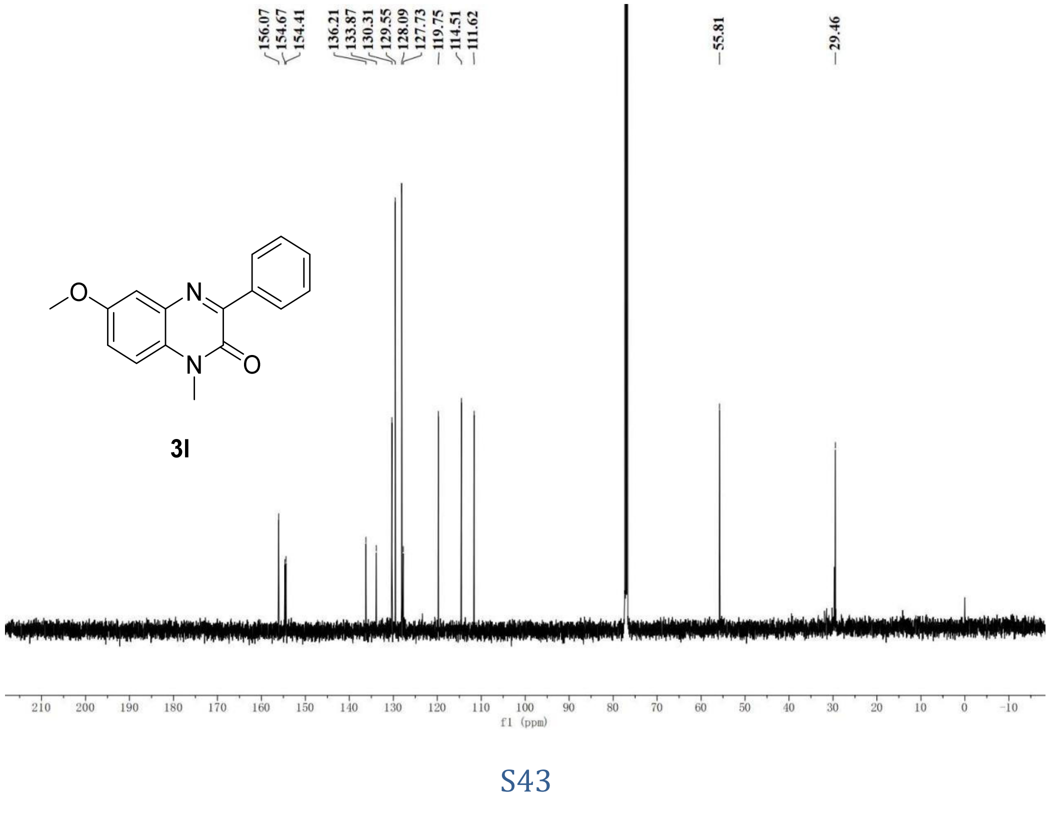
156.07 154.67 154.41
136.21 133.87 130.31 129.55 128.09 127.73 119.75 114.51 111.62
55.81
29.46
3l
f1 (ppm)

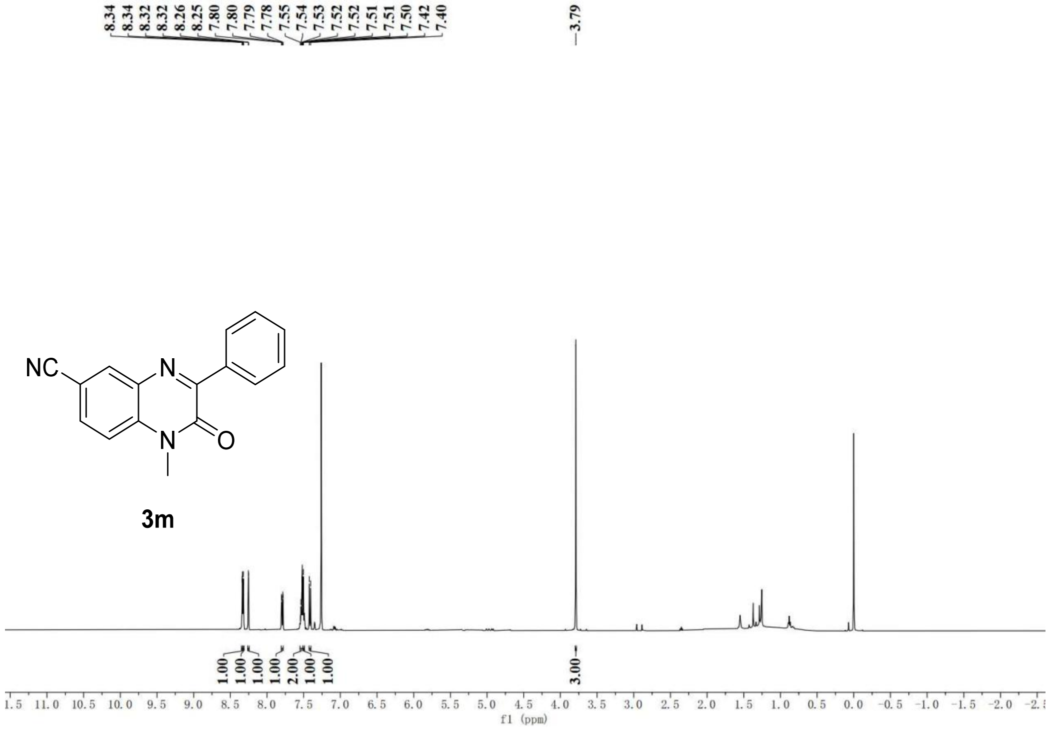
8.34
8.34
8.32
8.32
8.26
8.25
7.80
7.80
7.79
7.78
7.55
7.54
7.53
7.52
7.52
7.51
7.51
7.50
7.42
7.40
3.79
NC
N
N
O
3m
1.00
1.00
1.00
1.00
2.00
1.00
1.00
3.00
f1 (ppm)

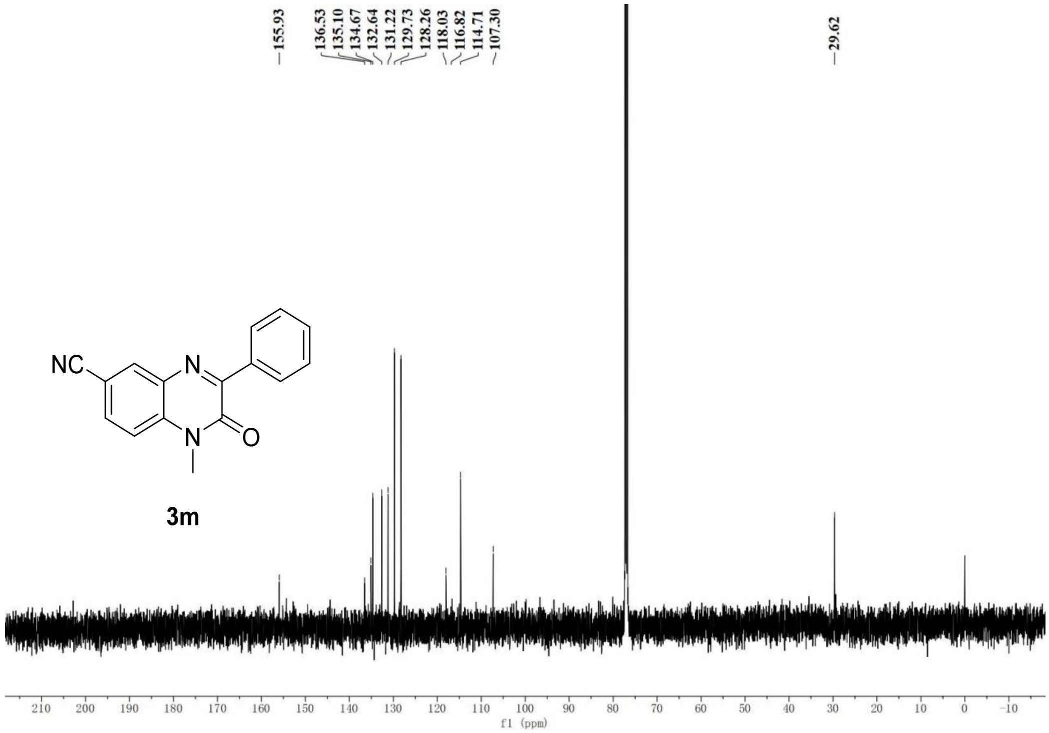
155.93
136.53
135.10
134.67
132.64
131.22
129.73
128.26
118.03
116.82
114.71
107.30
29.62
NC
N
N
O
3m
f1 (ppm)

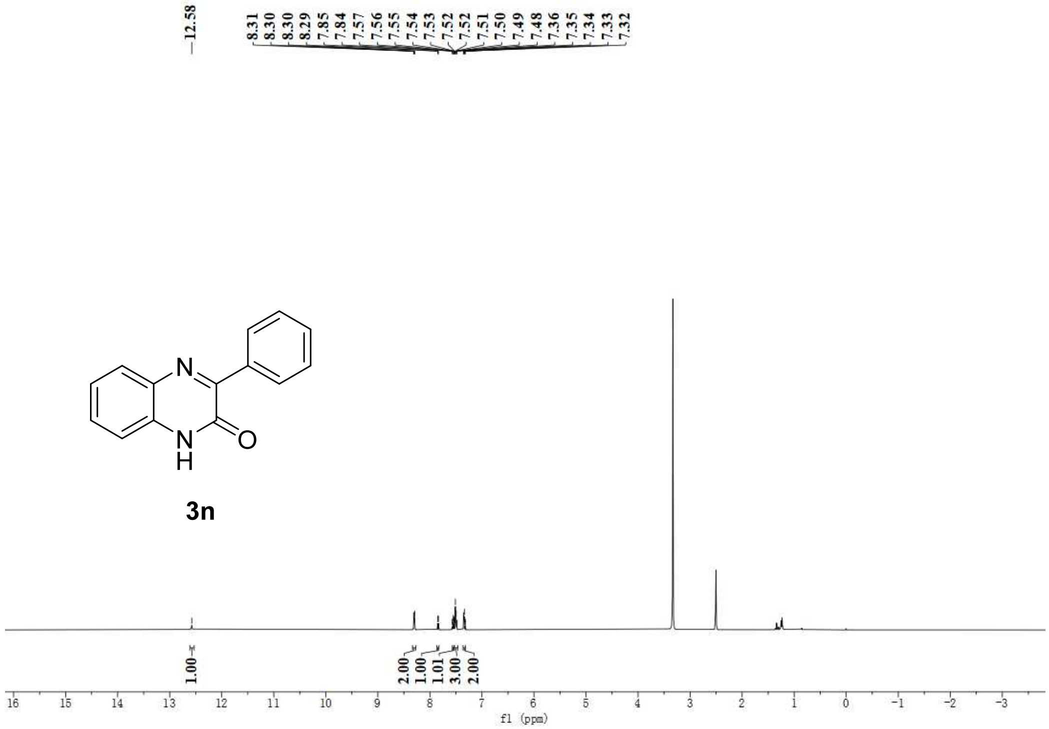
12.58
8.31
8.30
8.30
8.29
7.85
7.84
7.57
7.56
7.55
7.54
7.53
7.52
7.52
7.51
7.50
7.49
7.48
7.36
7.35
7.34
7.33
7.32
3n
1.00
2.00
1.00
1.01
3.00
2.00
f1 (ppm)

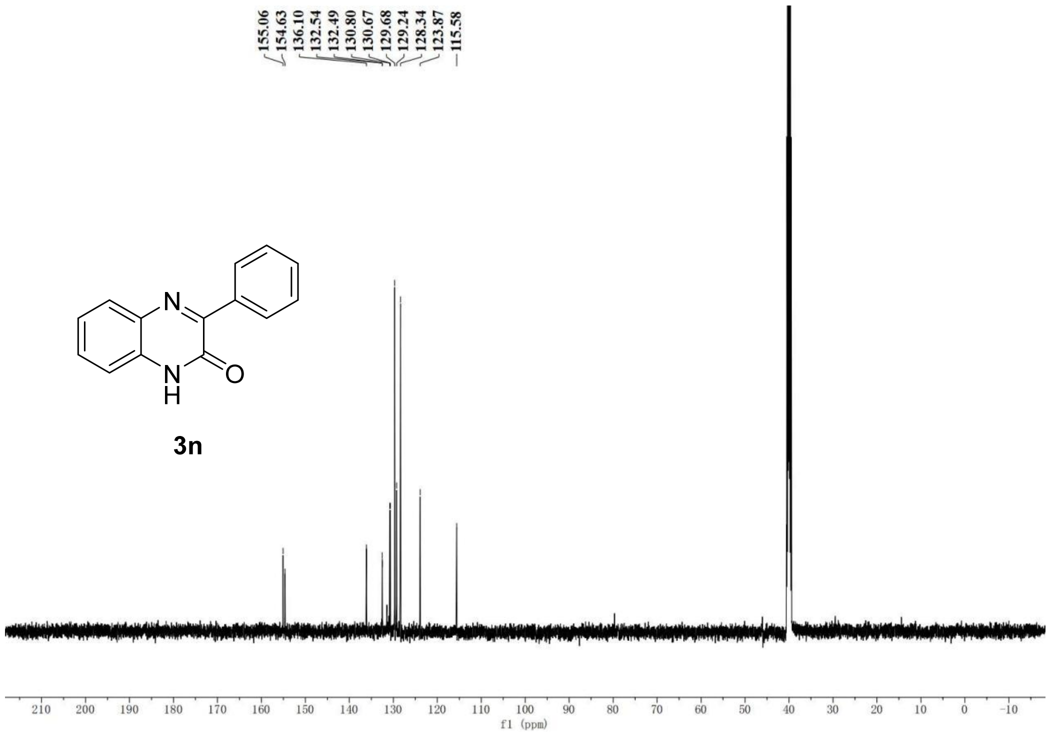
155.06
154.63
136.10
132.54
132.49
130.80
130.67
129.68
129.24
128.34
123.87
115.58
3n
f1 (ppm)

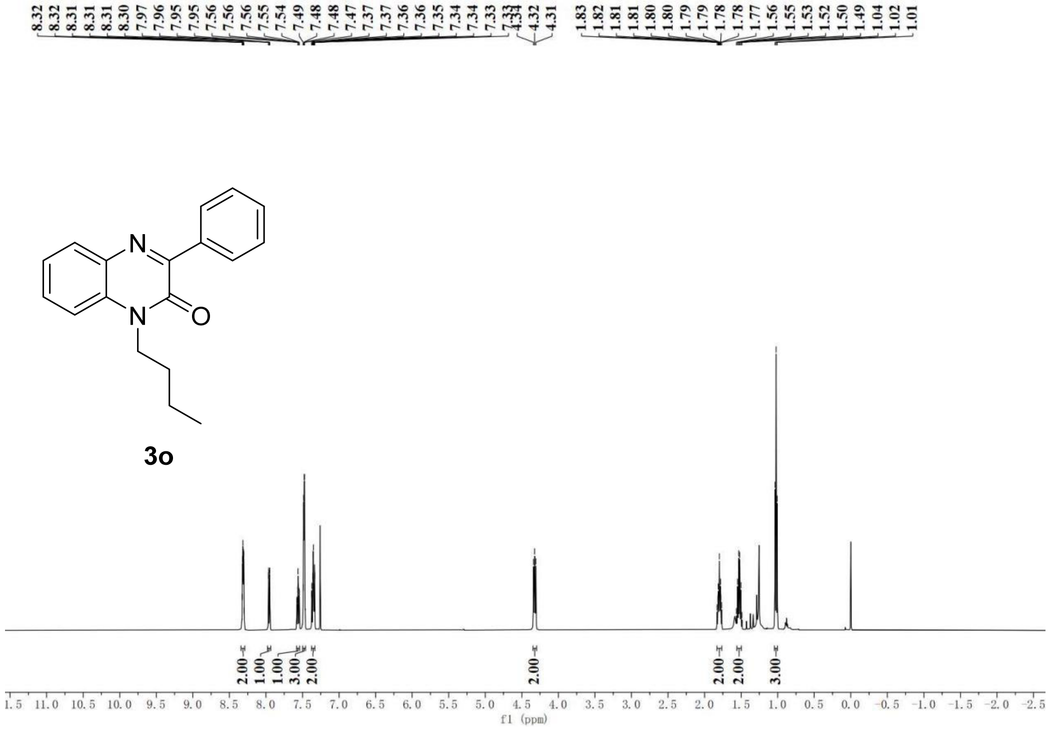
8.32 8.32 8.31 8.31 8.30 7.97 7.96 7.95 7.95 7.56 7.56 7.55 7.54 7.49 7.48 7.48 7.47 7.37 7.37 7.36 7.36 7.35 7.34 7.34 7.33 7.33 4.33 4.32 4.31
1.83 1.82 1.81 1.81 1.80 1.80 1.79 1.79 1.78 1.78 1.77 1.56 1.55 1.53 1.52 1.50 1.49 1.04 1.02 1.01
N
N
O
3o
2.00 1.00 1.00 3.00 2.00 2.00 2.00 2.00 3.00
f1 (ppm)

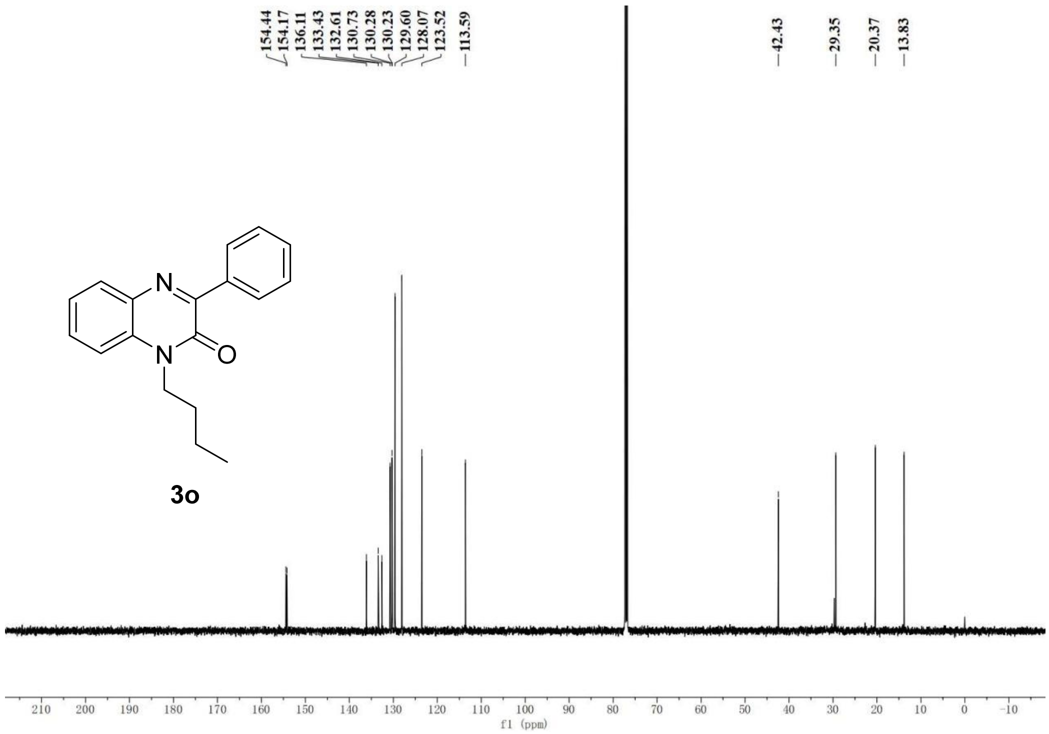
154.44 154.17 136.11 133.43 132.61 130.73 130.28 130.23 129.60 128.07 123.52 113.59
42.43 29.35 20.37 13.83
N
N
O
3o
f1 (ppm)

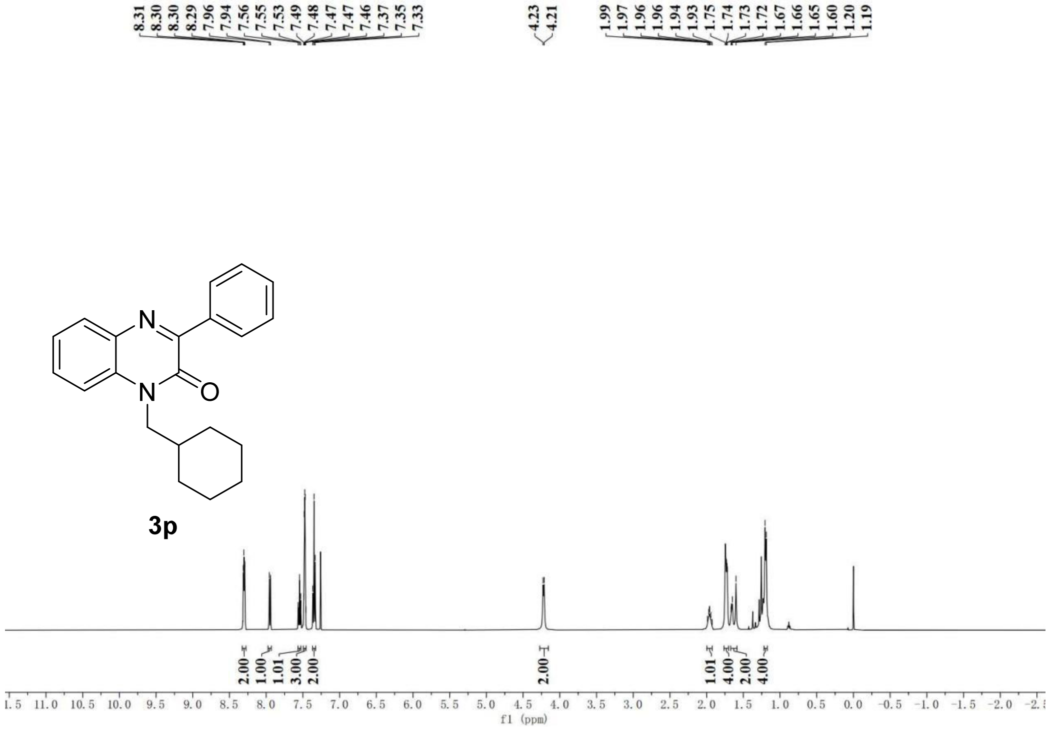
8.31 8.30 8.30 8.29 7.96 7.94 7.56 7.55 7.53 7.49 7.48 7.47 7.47 7.46 7.37 7.35 7.33
4.23 4.21
1.99 1.97 1.96 1.96 1.94 1.93 1.75 1.74 1.73 1.72 1.67 1.66 1.65 1.60 1.20 1.19
N
N
O
3p
2.00 1.00 1.01 3.00 2.00
2.00
1.01 4.00 2.00 4.00
f1 (ppm)

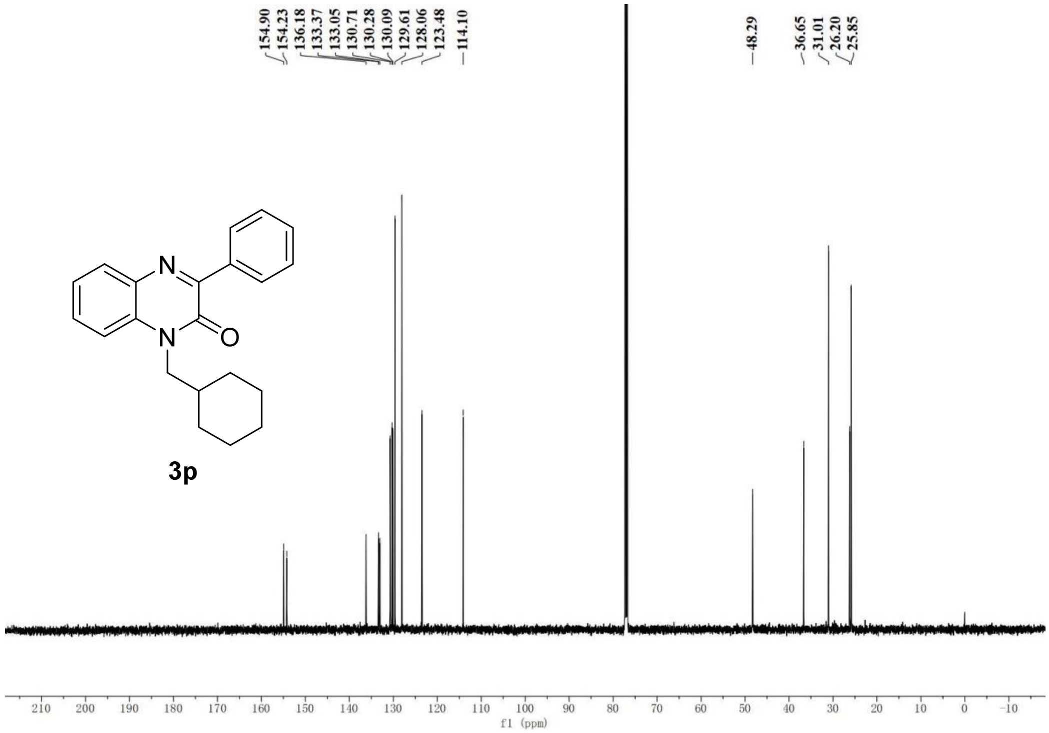
154.90 154.23 136.18 133.37 133.05 130.71 130.28 130.09 129.61 128.06 123.48 114.10
48.29
36.65 31.01 26.20 25.85
N
N
O
3p
f1 (ppm)

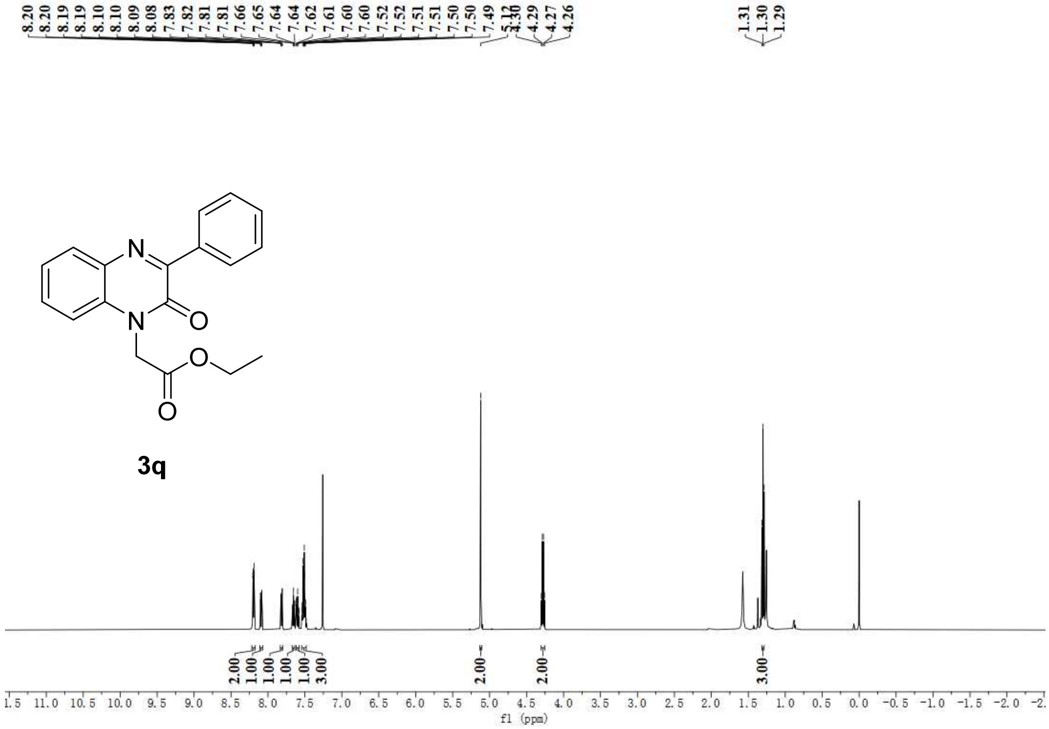
8.20
8.20
8.19
8.19
8.10
8.10
8.09
8.08
7.83
7.82
7.81
7.81
7.66
7.65
7.64
7.64
7.62
7.61
7.60
7.60
7.52
7.52
7.51
7.51
7.50
7.49
5.12
4.30
4.29
4.27
4.26
1.31
1.30
1.29
N
N
O
O
O
2.00
1.00
1.00
1.00
1.00
3.00
2.00
2.00
3.00
f1 (ppm)

3q

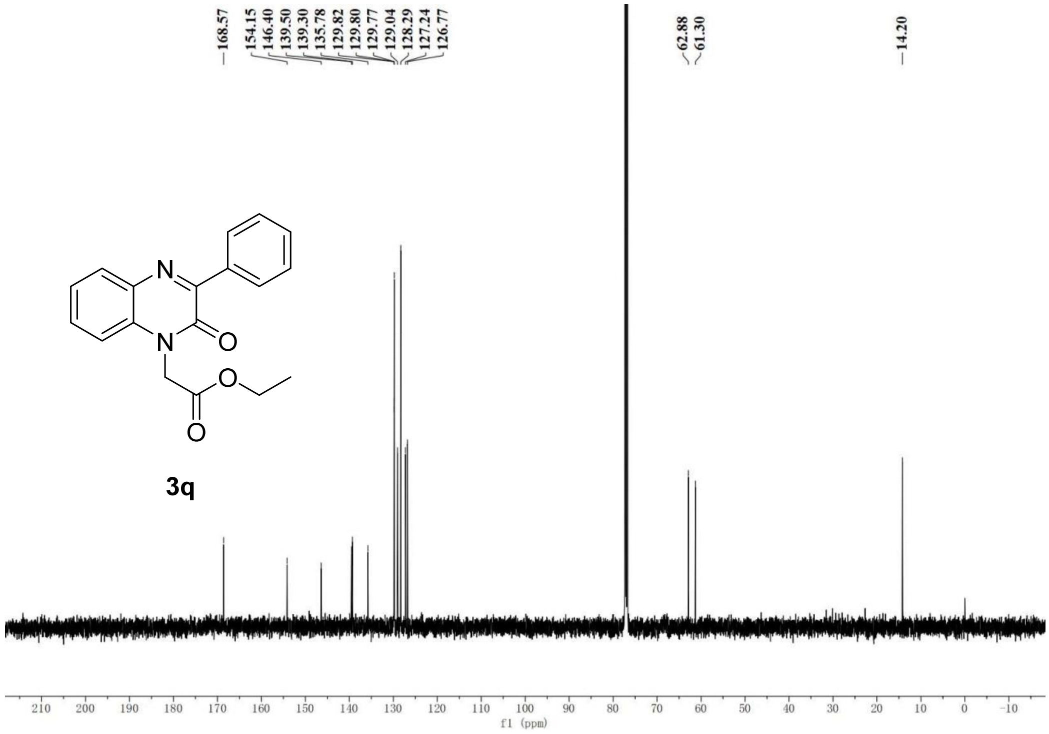
168.57
154.15
146.40
139.50
139.30
135.78
129.82
129.80
129.77
129.04
128.29
127.24
126.77
62.88
61.30
14.20
N
N
O
O
O
f1 (ppm)

3q

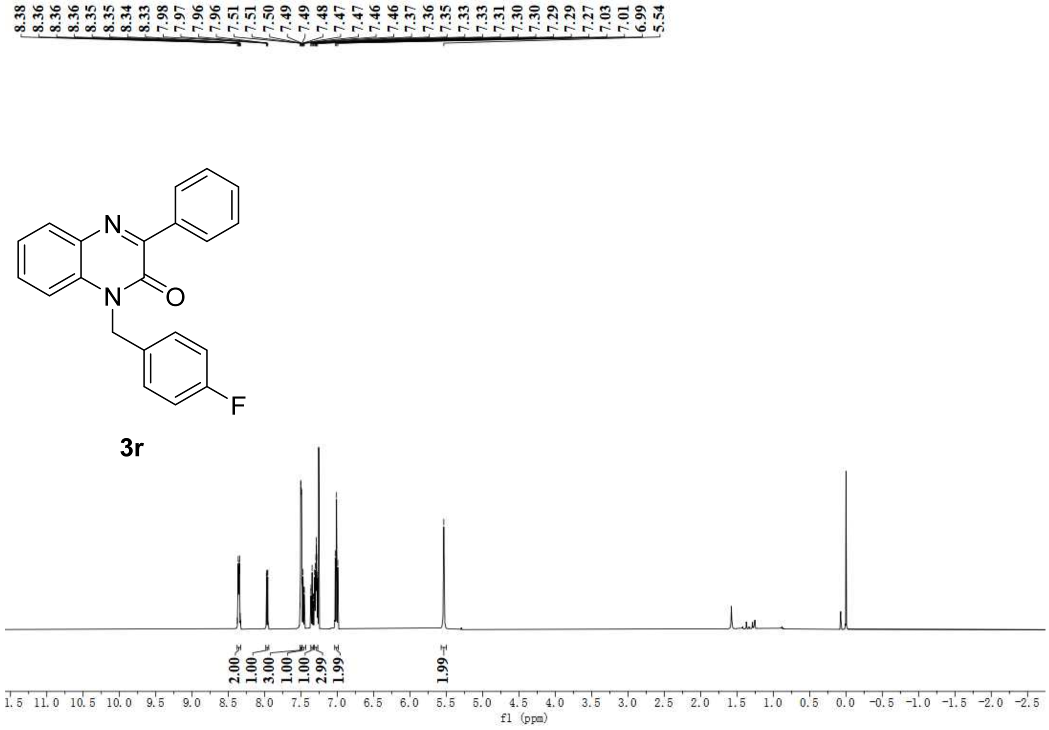

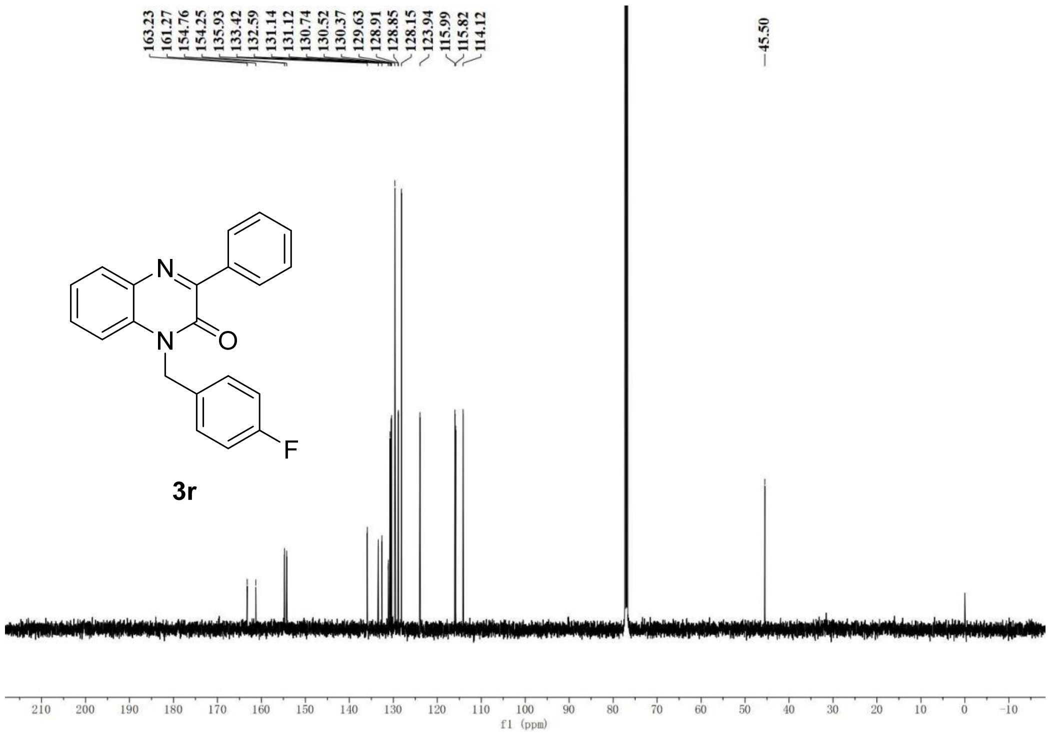

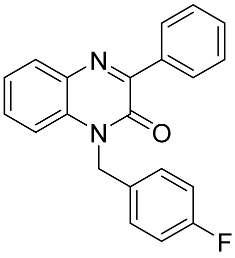
N
N
O
F

3r

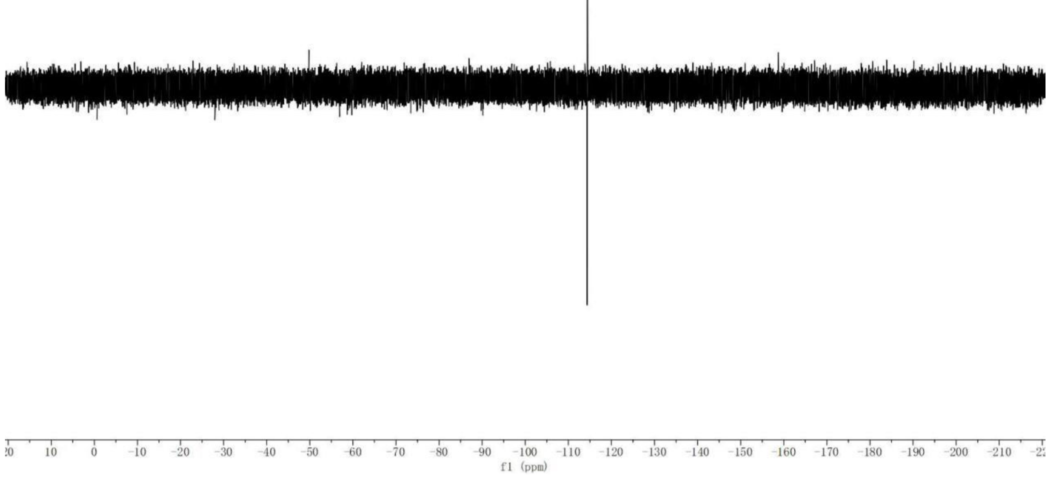
-114.38
20 10 0 -10 -20 -30 -40 -50 -60 -70 -80 -90 -100 -110 -120 -130 -140 -150 -160 -170 -180 -190 -200 -210
f1 (ppm)

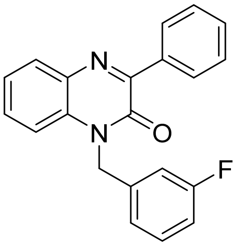
N
N
O
F

3s

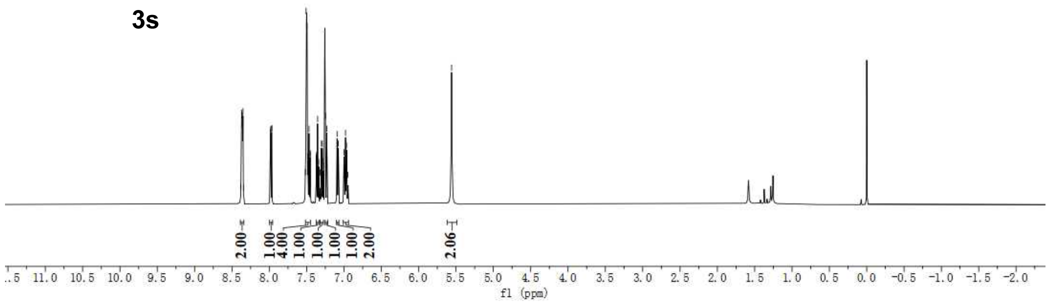
8.38 8.37 8.37 8.36 8.36 8.36 8.35 8.34 7.98 7.98 7.97 7.97 7.52 7.51 7.50 7.49 7.49 7.48 7.47 7.45 7.45 7.37 7.35 7.34 7.32 7.31 7.30 7.29 7.29 7.27 7.25 7.23 7.09 7.08 7.00 6.98 6.97 6.96 6.95 5.56
2.00 1.00 4.00 1.00 1.00 1.00 1.00 2.00 2.06
11.0 10.5 10.0 9.5 9.0 8.5 8.0 7.5 7.0 6.5 6.0 5.5 5.0 4.5 4.0 3.5 3.0 2.5 2.0 1.5 1.0 0.5 0.0 -0.5 -1.0 -1.5 -2.0
f1 (ppm)

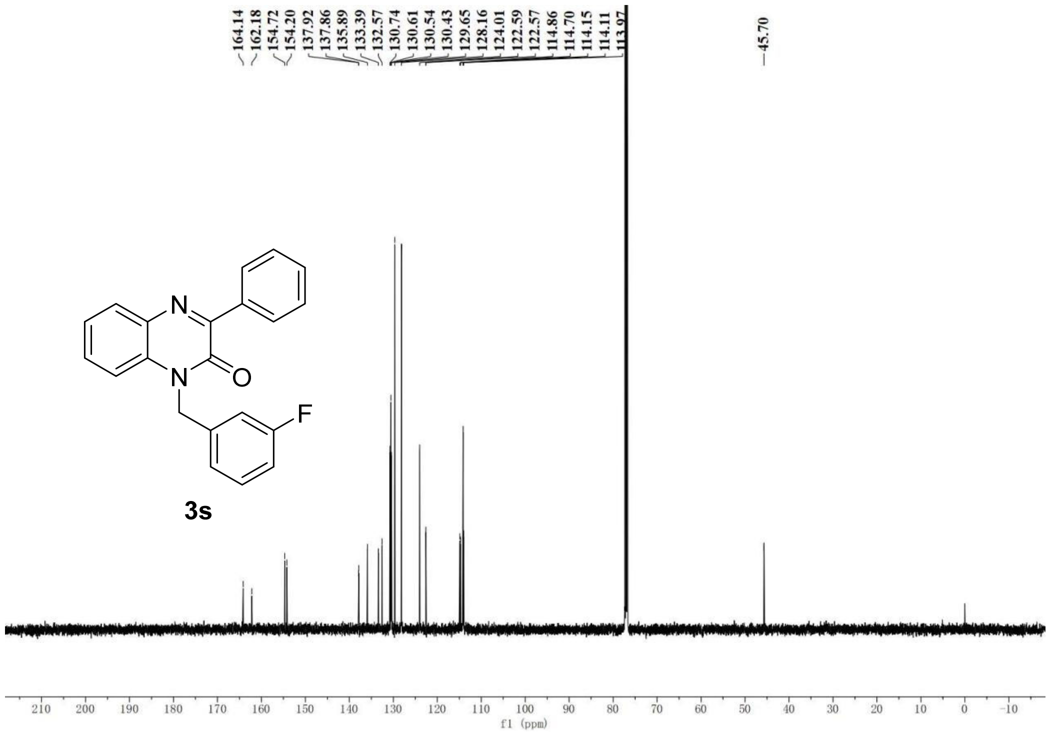
164.14
162.18
154.72
154.20
137.92
137.86
135.89
133.39
132.57
130.74
130.61
130.54
130.43
129.65
128.16
124.01
122.59
122.57
114.86
114.70
114.15
114.11
113.97
45.70
N
N
O
F
3s
f1 (ppm)

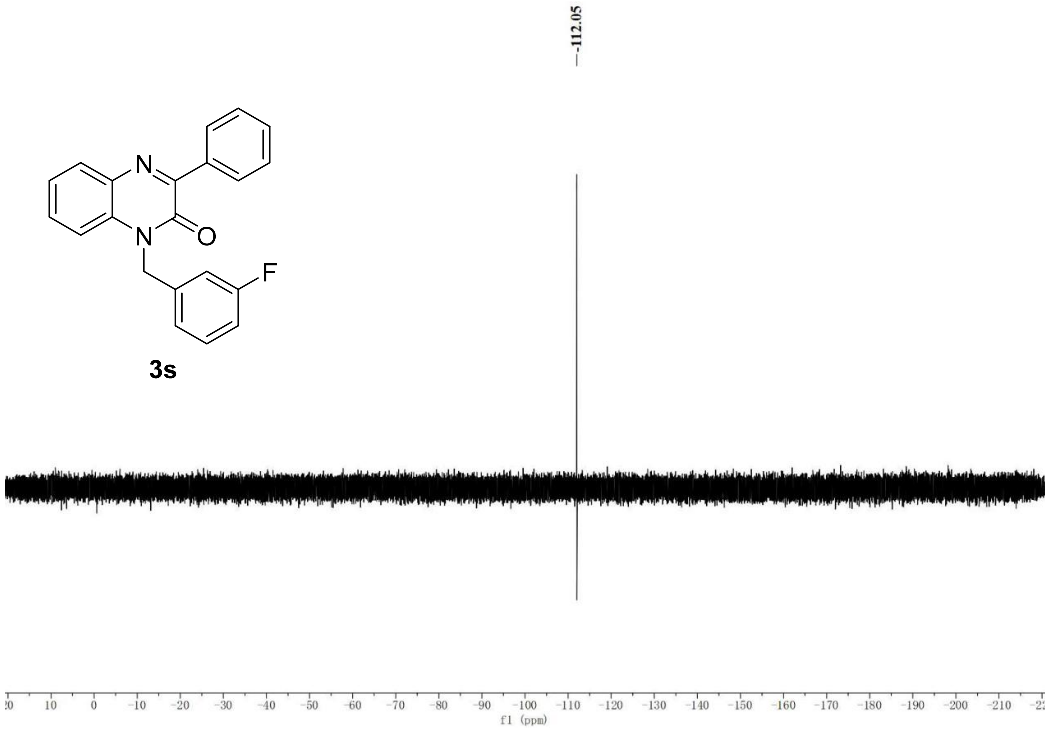
-112.05
N
N
O
F
3s
f1 (ppm)

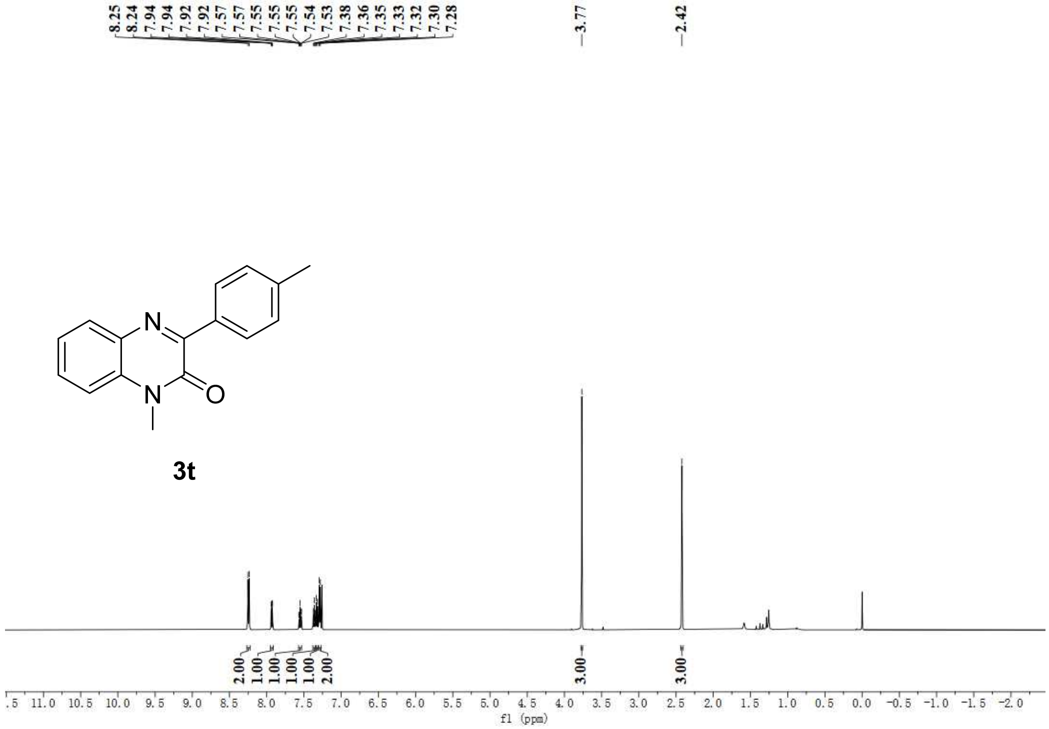
8.25
8.24
7.94
7.94
7.92
7.92
7.57
7.57
7.55
7.55
7.55
7.54
7.53
7.38
7.36
7.35
7.33
7.32
7.30
7.28
3.77
2.42
N
N
O
3t
2.00
1.00
1.00
1.00
1.00
2.00
3.00
3.00
f1 (ppm)

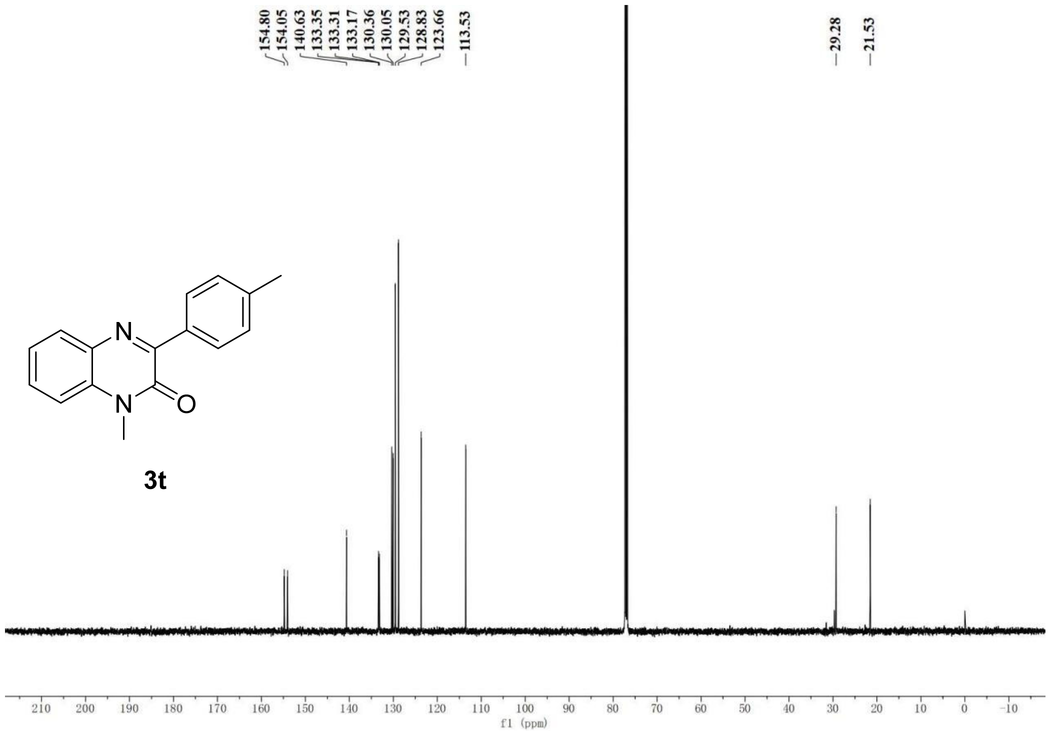
154.80
154.05
140.63
133.35
133.31
133.17
130.36
130.05
129.53
128.83
123.66
113.53
29.28
21.53
N
N
O
3t
f1 (ppm)

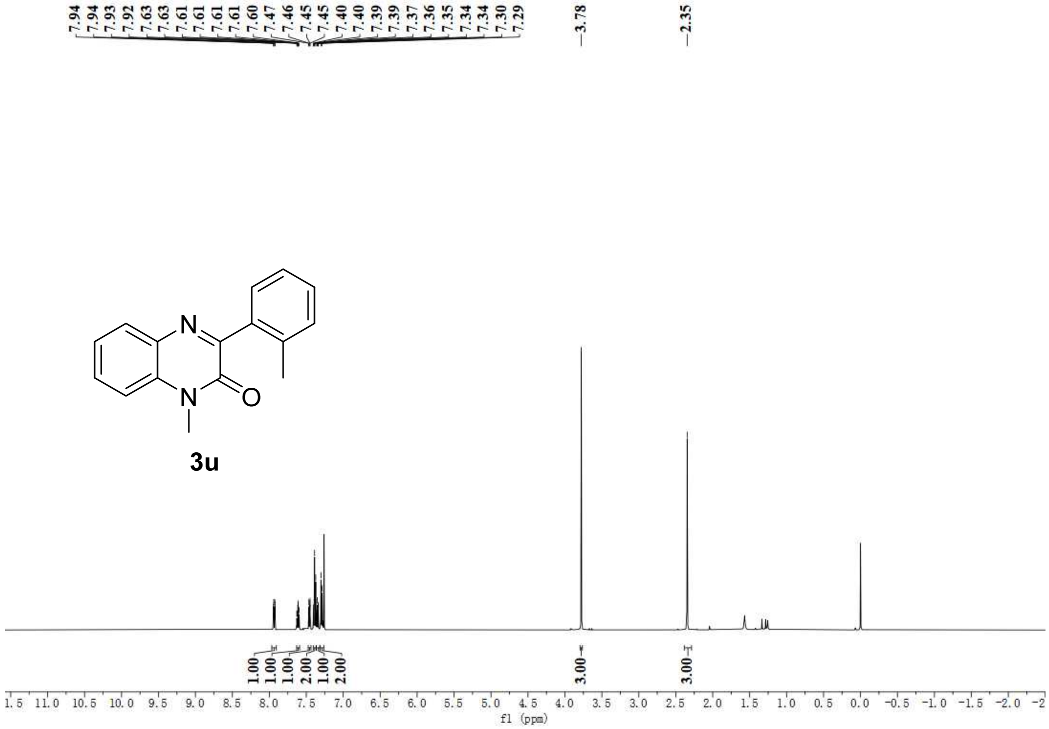
7.94
7.94
7.93
7.92
7.63
7.63
7.61
7.61
7.61
7.61
7.60
7.47
7.46
7.45
7.45
7.40
7.40
7.39
7.39
7.37
7.36
7.35
7.34
7.34
7.30
7.29
3.78
2.35
N
N
O
3u
1.00
1.00
1.00
2.00
1.00
2.00
3.00
3.00
f1 (ppm)

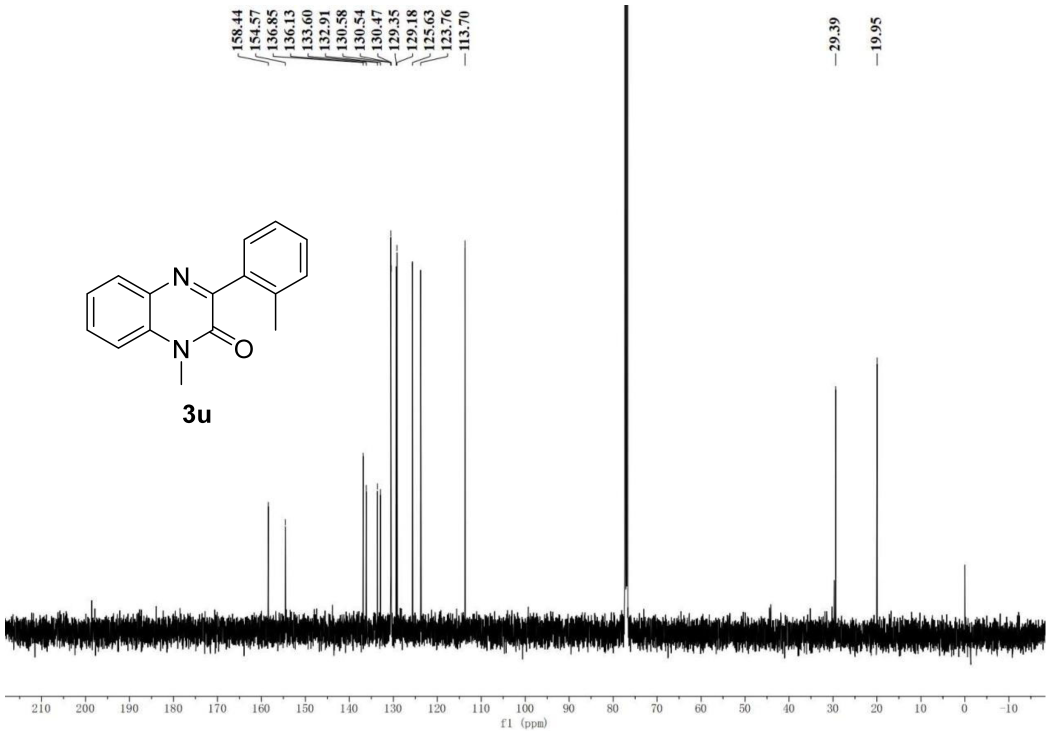
158.44
154.57
136.85
136.13
133.60
132.91
130.58
130.54
130.47
129.35
129.18
125.63
123.76
113.70
29.39
19.95
N
N
O
3u
f1 (ppm)

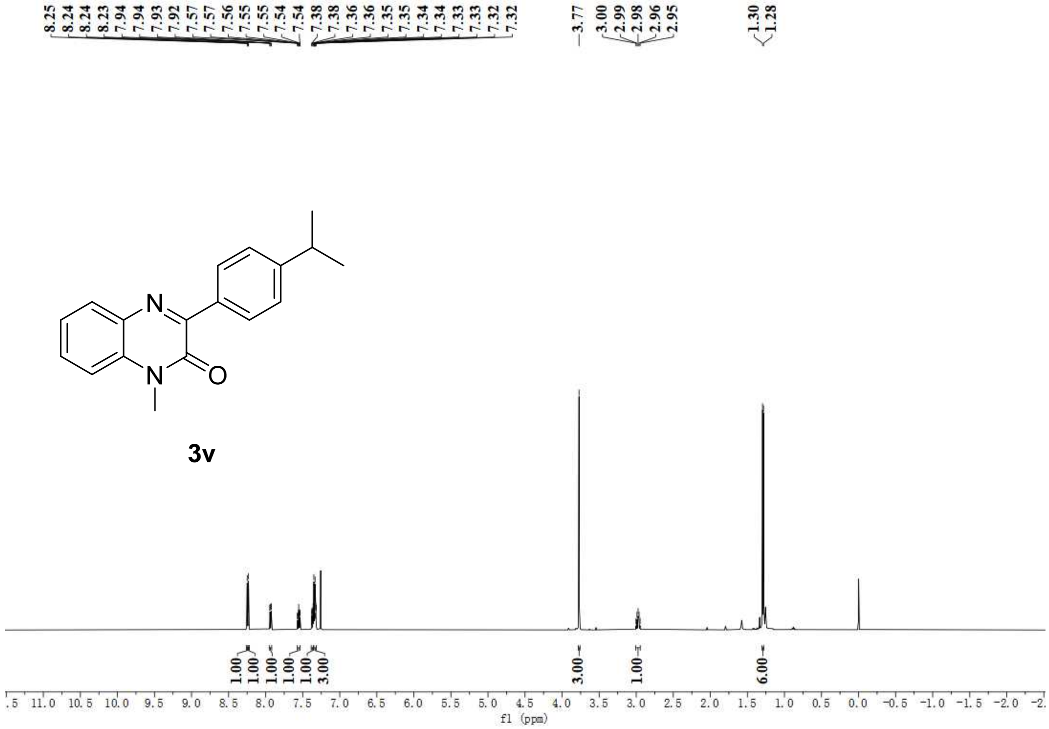
8.25
8.24
8.24
8.23
7.94
7.94
7.93
7.92
7.57
7.57
7.56
7.55
7.55
7.54
7.54
7.38
7.38
7.36
7.36
7.35
7.35
7.34
7.34
7.33
7.33
7.32
7.32
3.77
3.00
2.99
2.98
2.96
2.95
1.30
1.28
N
N
O
1.00
1.00
1.00
1.00
3.00
3.00
1.00
6.00
f1 (ppm)

3v

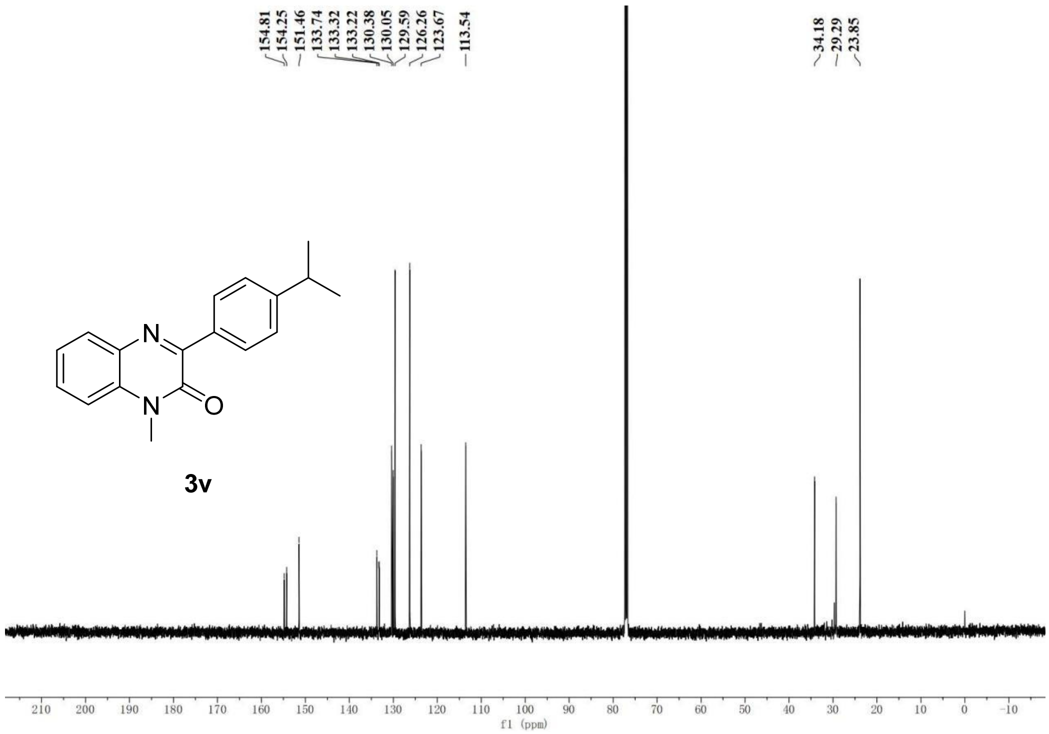
154.81
154.25
151.46
133.74
133.32
133.22
130.38
130.05
129.59
126.26
123.67
113.54
34.18
29.29
23.85
N
N
O
f1 (ppm)

3v

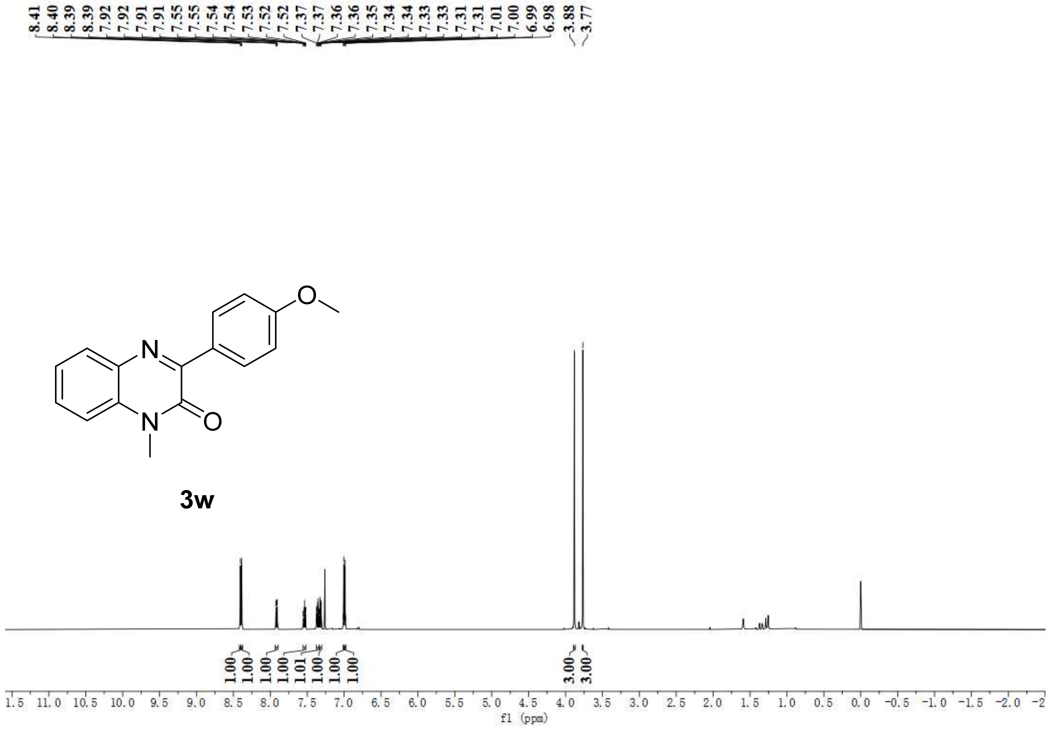

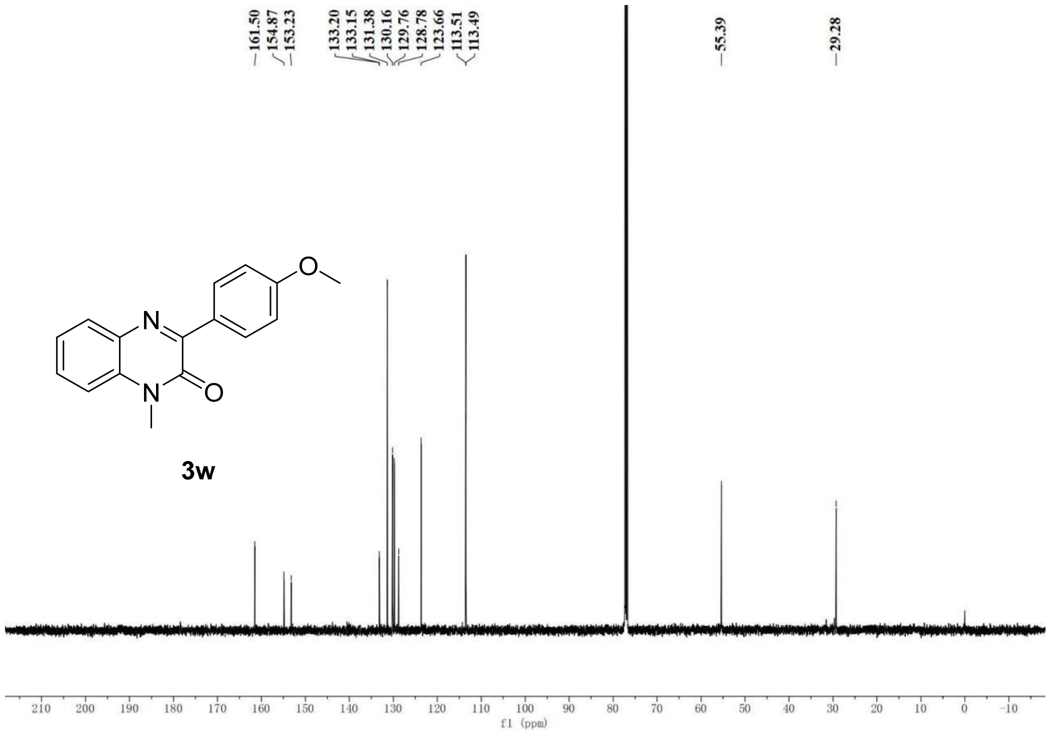

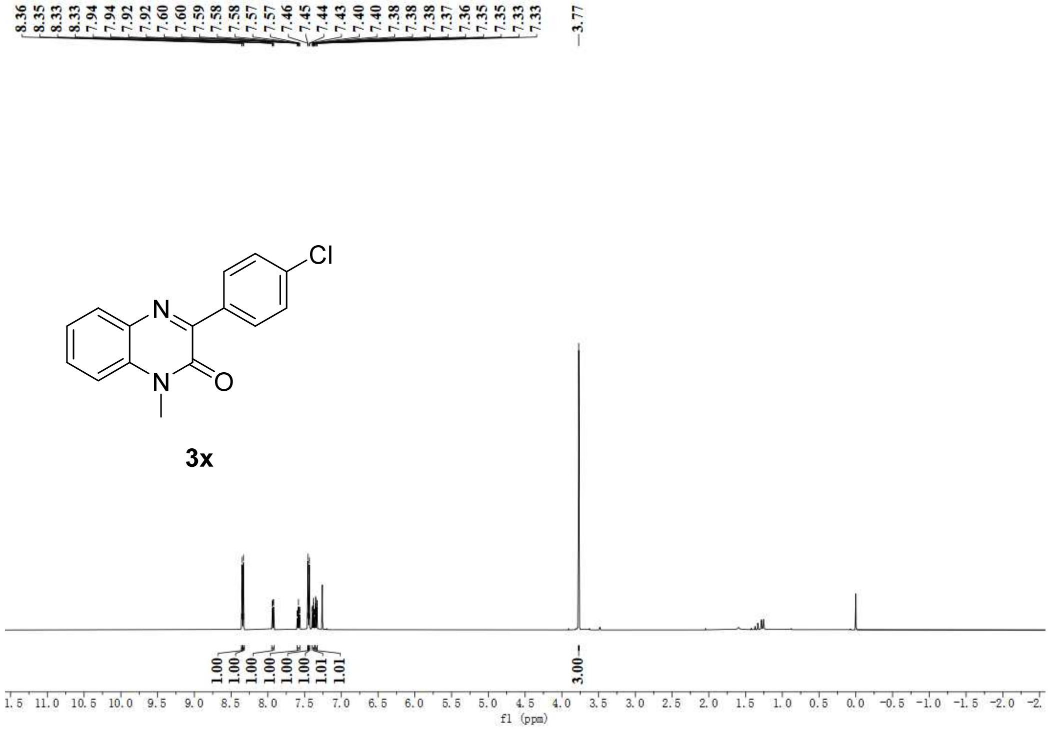
8.36
8.35
8.33
8.33
7.94
7.94
7.92
7.92
7.60
7.59
7.58
7.58
7.57
7.57
7.46
7.45
7.44
7.43
7.40
7.40
7.38
7.38
7.38
7.37
7.36
7.35
7.35
7.33
7.33
3.77
Cl
N
N
O
3x
1.00
1.00
1.00
1.00
1.00
1.01
1.01
3.00
f1 (ppm)

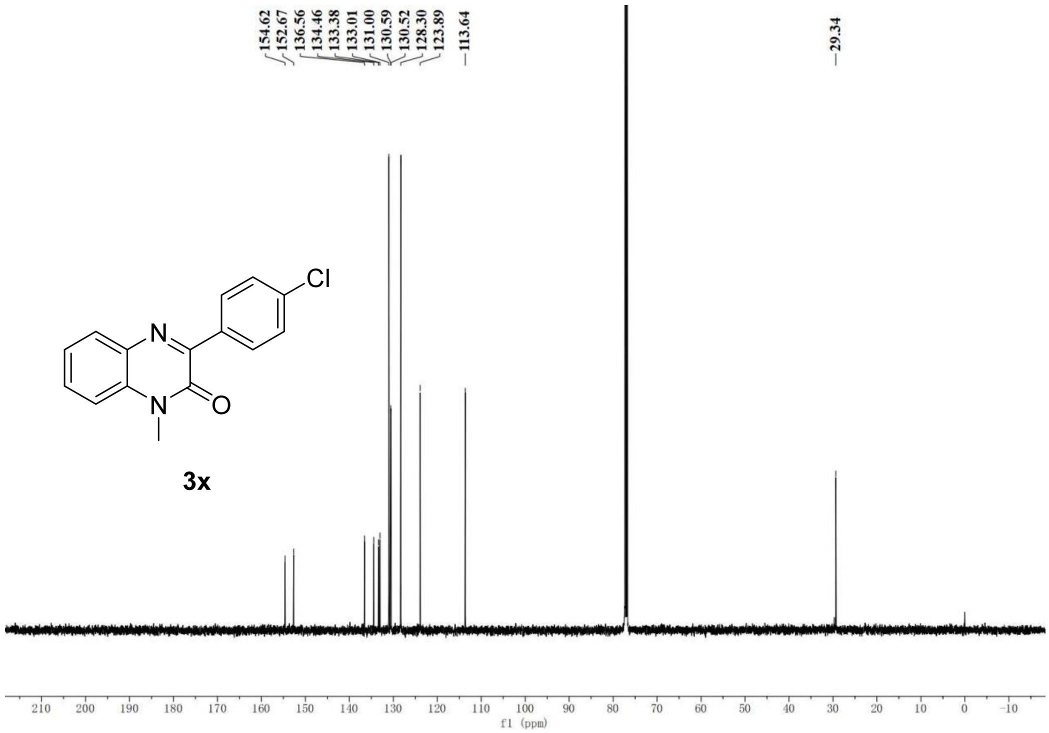
154.62
152.67
136.56
134.46
133.38
133.01
131.00
130.59
130.52
128.30
123.89
113.64
29.34
Cl
N
N
O
3x
f1 (ppm)

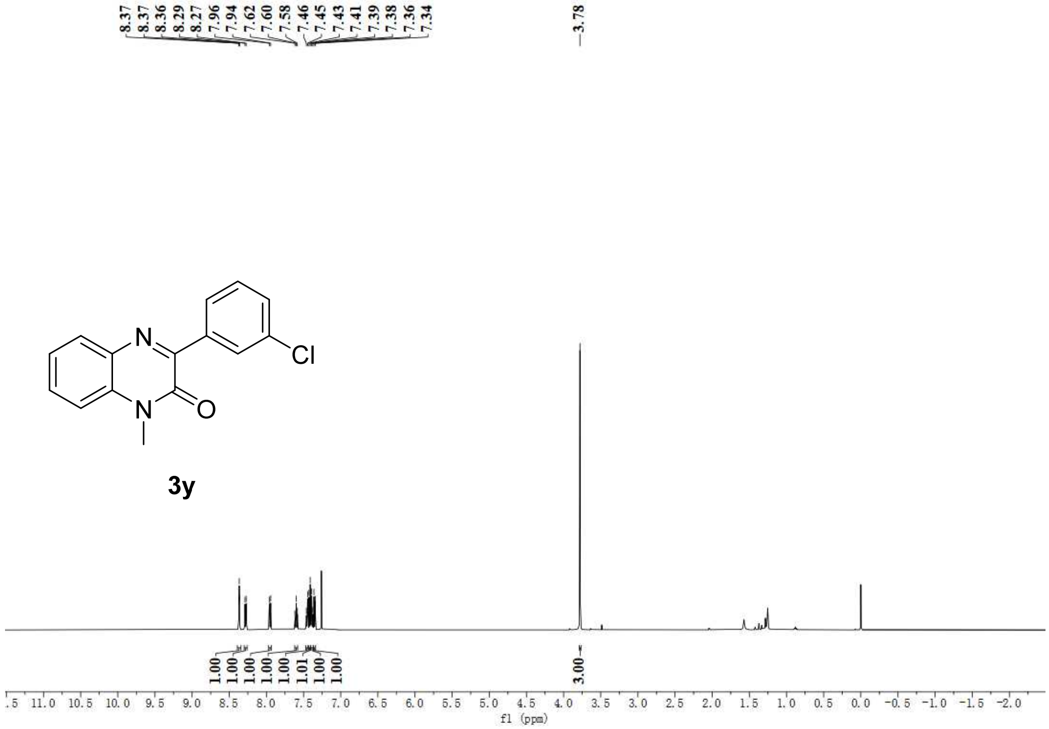
8.37
8.37
8.36
8.29
8.27
7.96
7.94
7.62
7.60
7.58
7.46
7.45
7.43
7.41
7.39
7.38
7.36
7.34
3.78
N
Cl
N
O
3y
1.00
1.00
1.00
1.00
1.00
1.01
1.00
1.00
3.00
f1 (ppm)

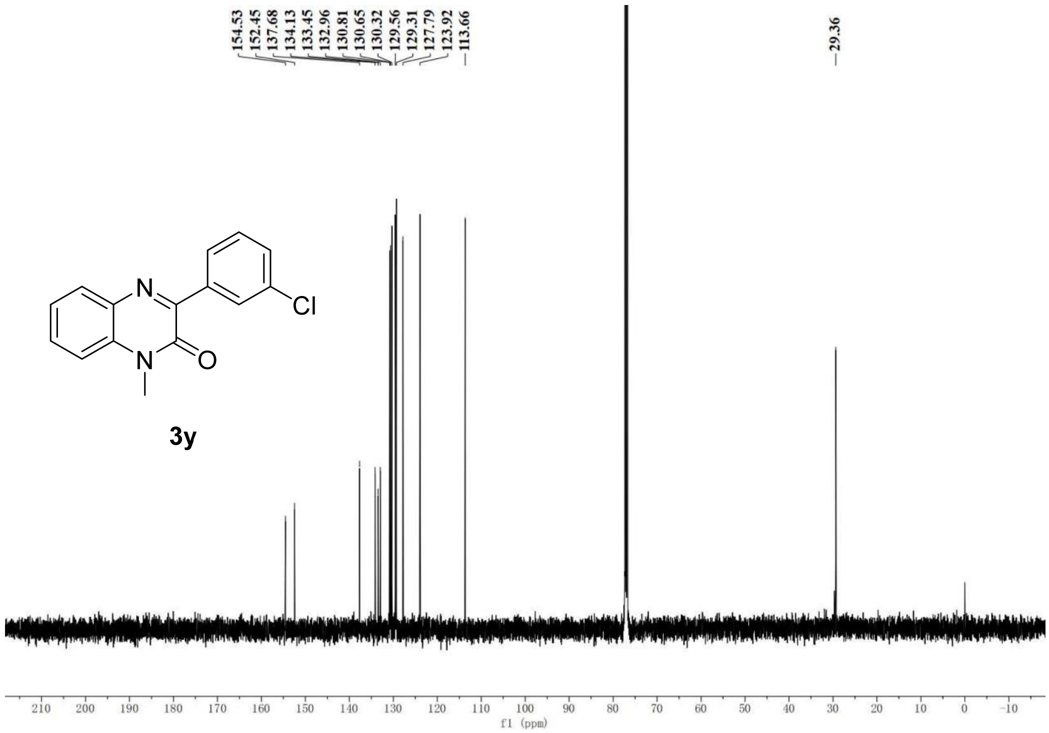
154.53
152.45
137.68
134.13
133.45
132.96
130.81
130.65
130.32
129.56
129.31
127.79
123.92
113.66
29.36
N
Cl
N
O
3y
f1 (ppm)

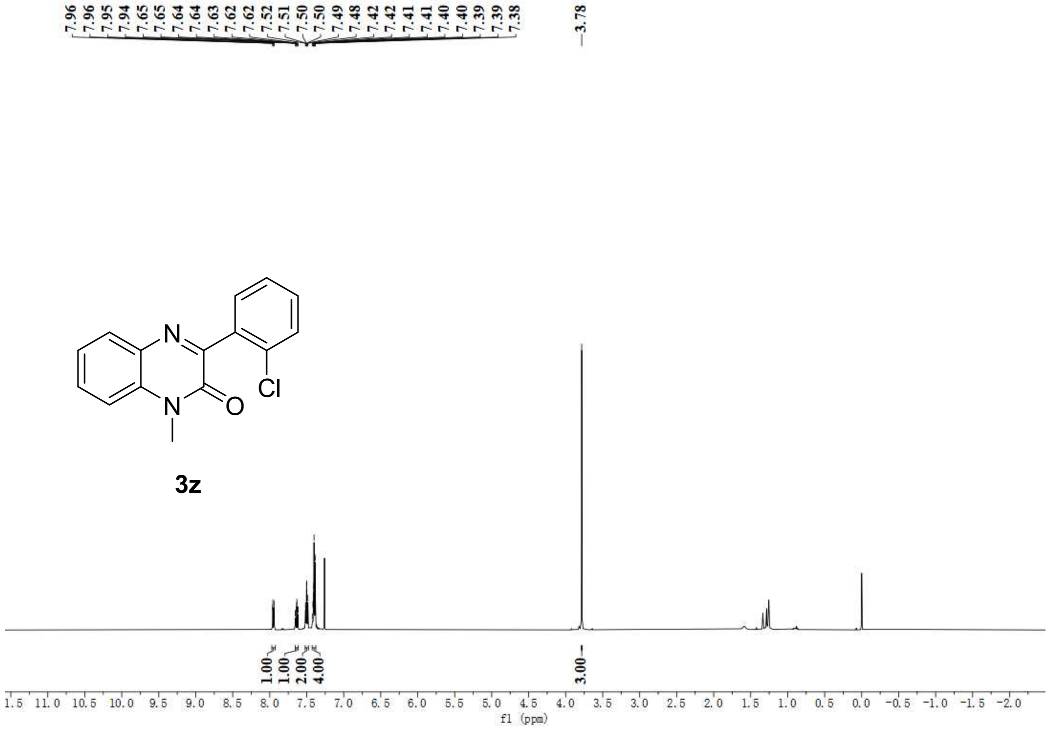
7.96
7.96
7.95
7.94
7.65
7.65
7.64
7.64
7.63
7.62
7.62
7.52
7.51
7.50
7.50
7.49
7.48
7.42
7.42
7.41
7.41
7.40
7.40
7.39
7.39
7.38
3.78
N
N
O
Cl
3z
1.00
1.00
2.00
4.00
3.00
f1 (ppm)

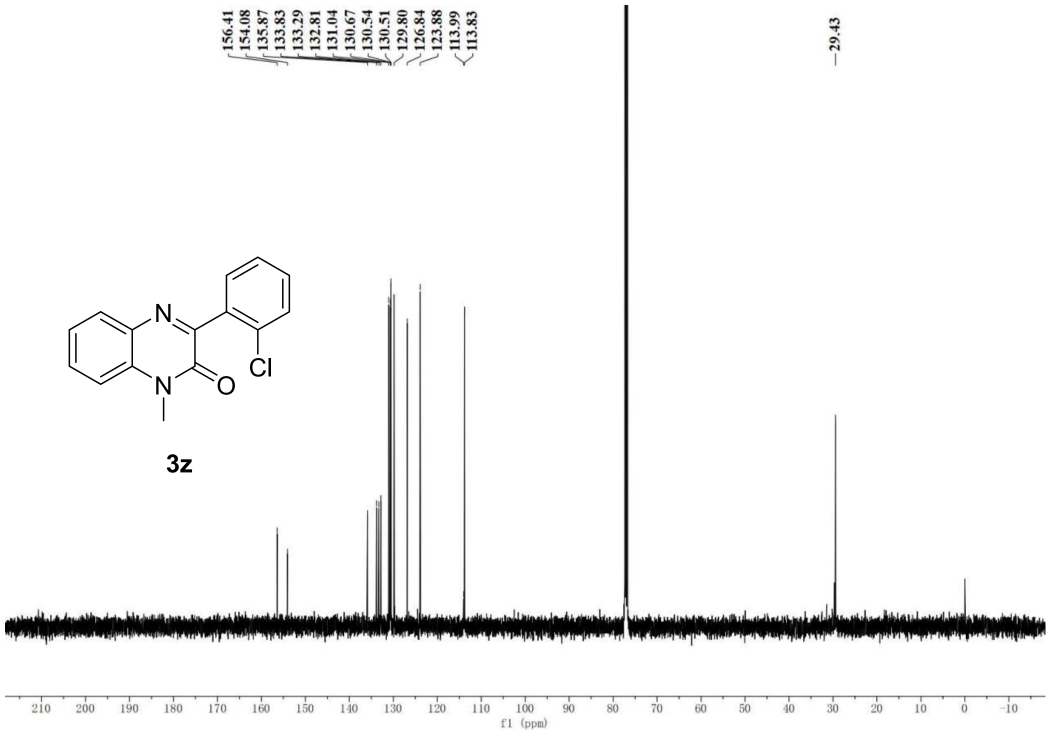
156.41
154.08
135.87
133.83
133.29
132.81
131.04
130.67
130.54
130.51
129.80
126.84
123.88
113.99
113.83
29.43
N
N
O
Cl
3z
f1 (ppm)

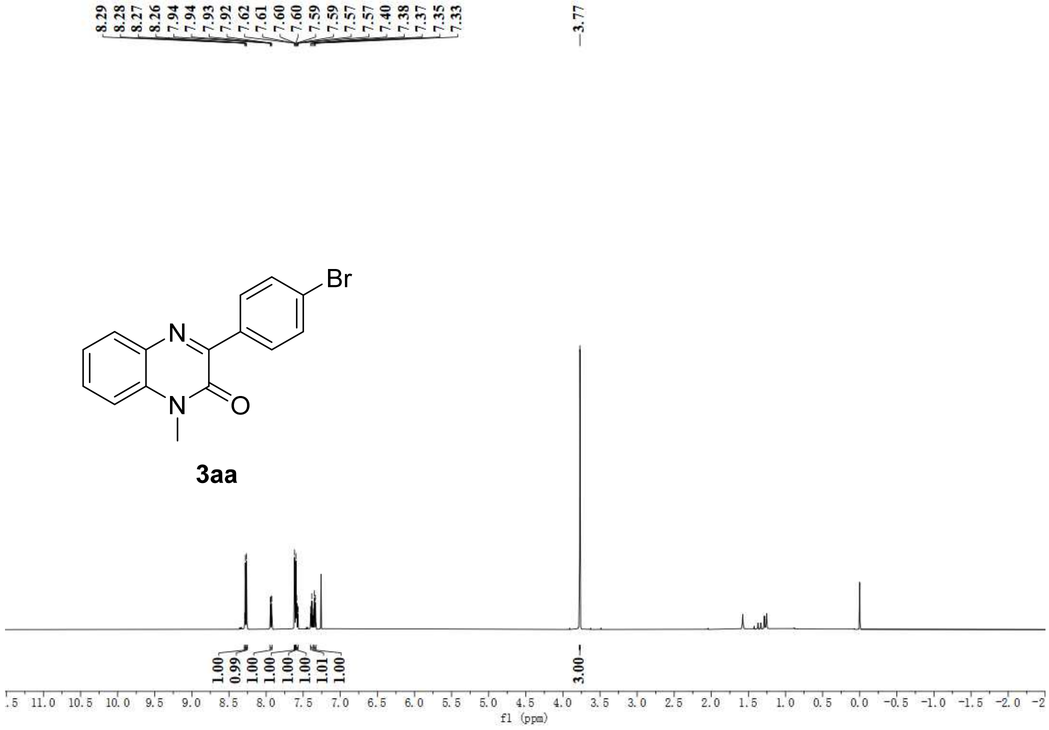
Br
N
N
O
3aa
8.29
8.28
8.27
8.26
7.94
7.94
7.93
7.92
7.62
7.61
7.60
7.60
7.59
7.59
7.57
7.57
7.40
7.38
7.37
7.35
7.33
3.77
1.00
0.99
1.00
1.00
1.00
1.00
1.01
1.00
3.00
f1 (ppm)

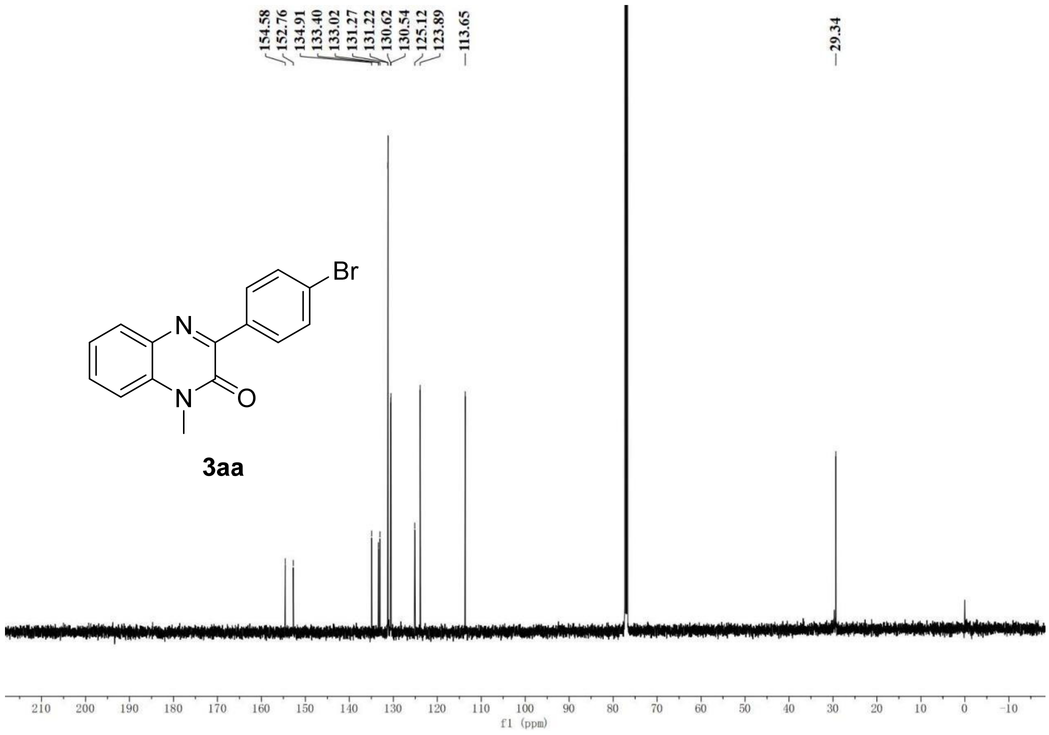
Br
N
N
O
3aa
154.58
152.76
134.91
133.40
133.02
131.27
131.22
130.62
130.54
125.12
123.89
113.65
29.34
f1 (ppm)

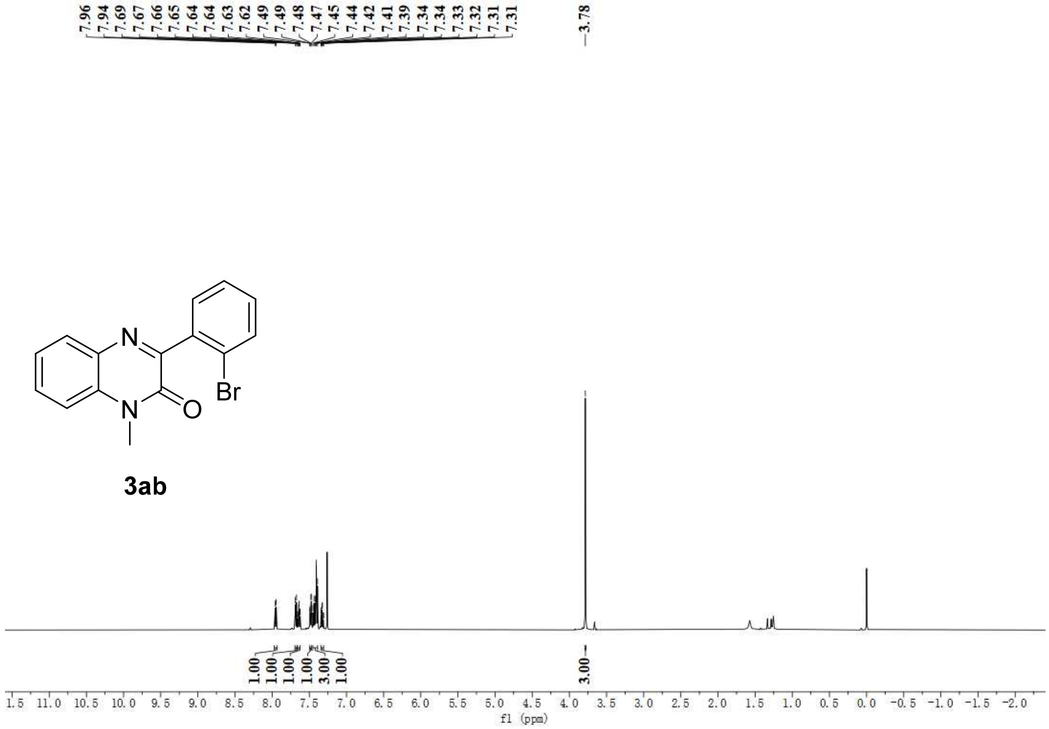
7.96
7.94
7.69
7.67
7.66
7.65
7.64
7.64
7.63
7.62
7.49
7.49
7.48
7.47
7.45
7.44
7.42
7.41
7.39
7.34
7.34
7.33
7.32
7.31
7.31
3.78
N
N
O
Br
3ab
1.00
1.00
1.00
1.00
3.00
1.00
3.00
f1 (ppm)

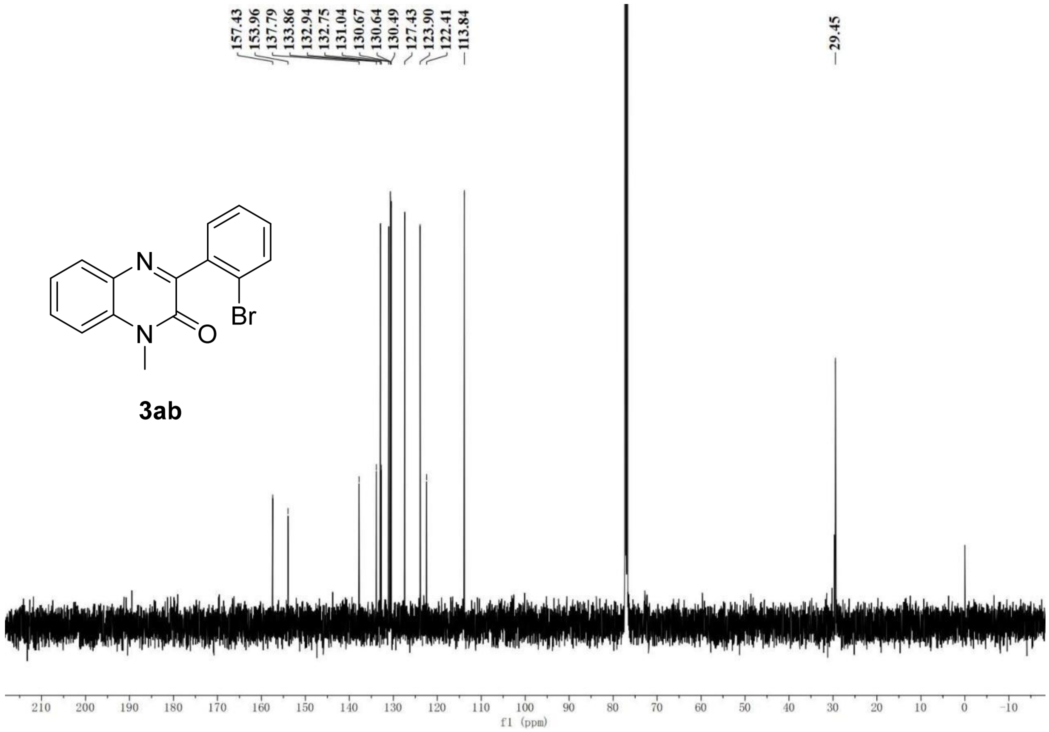
157.43
153.96
137.79
133.86
132.94
132.75
131.04
130.67
130.64
130.49
127.43
123.90
122.41
113.84
29.45
N
N
O
Br
3ab
f1 (ppm)

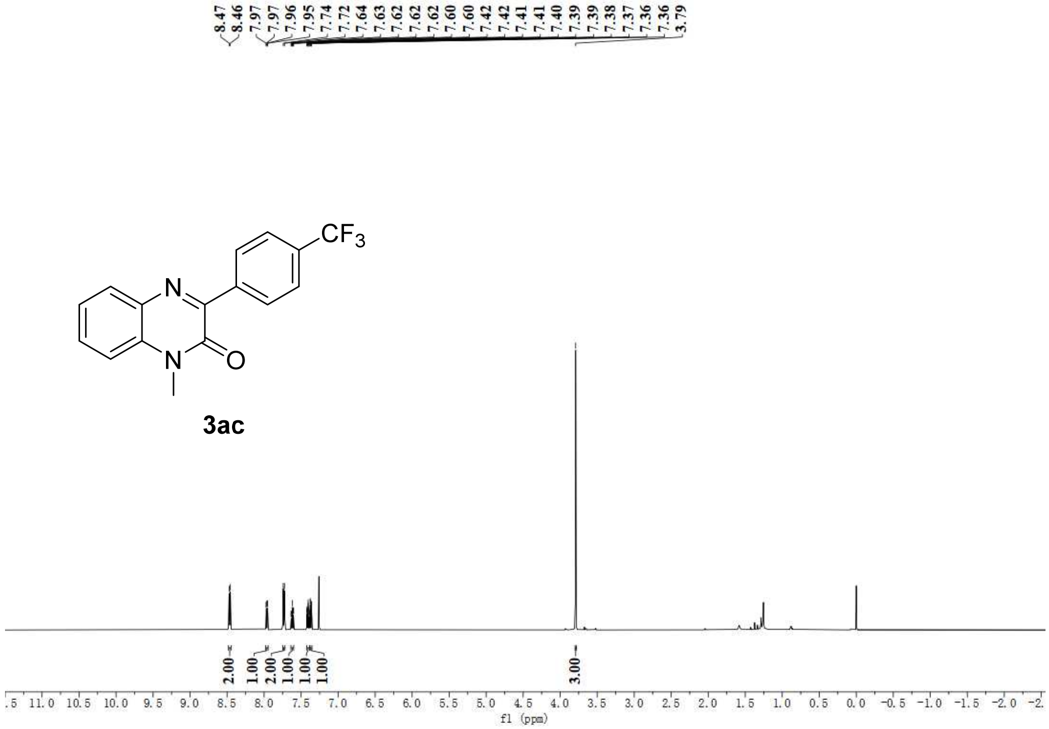

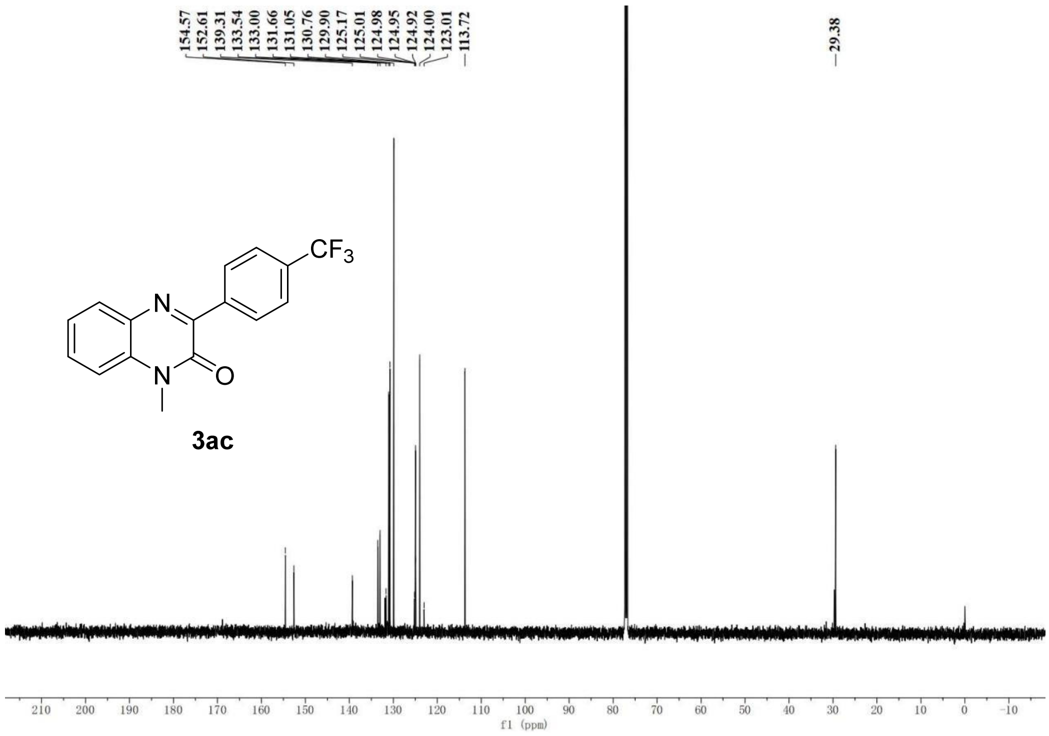

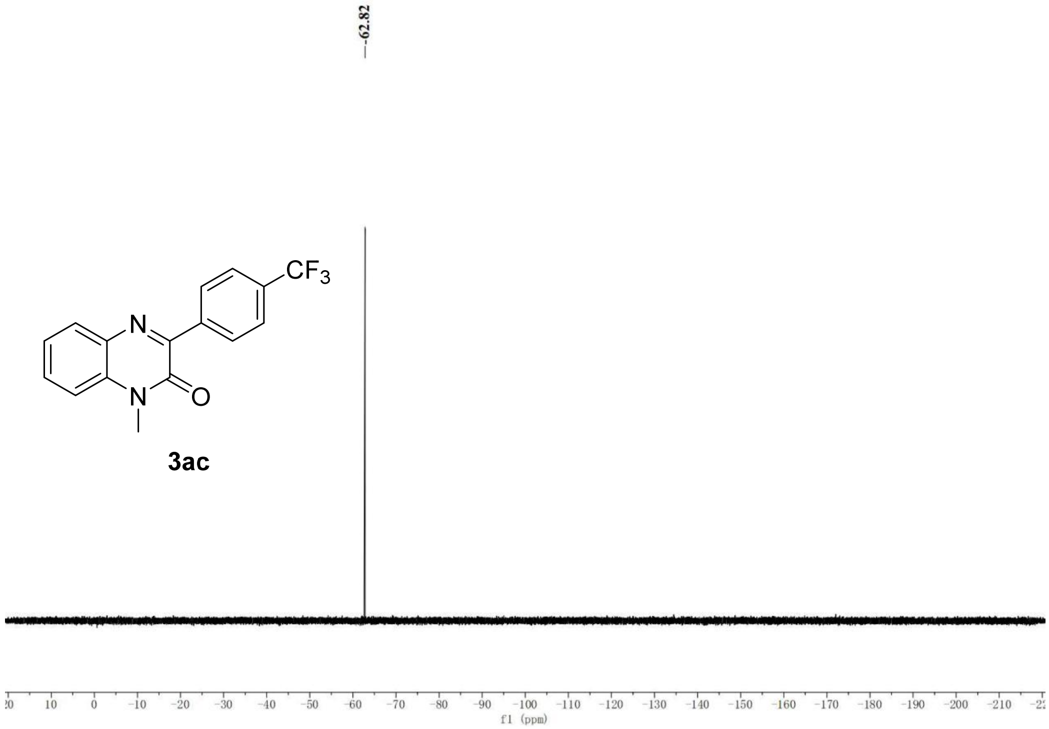

-62.82
CF3
N
N
O
3ac
f1 (ppm)

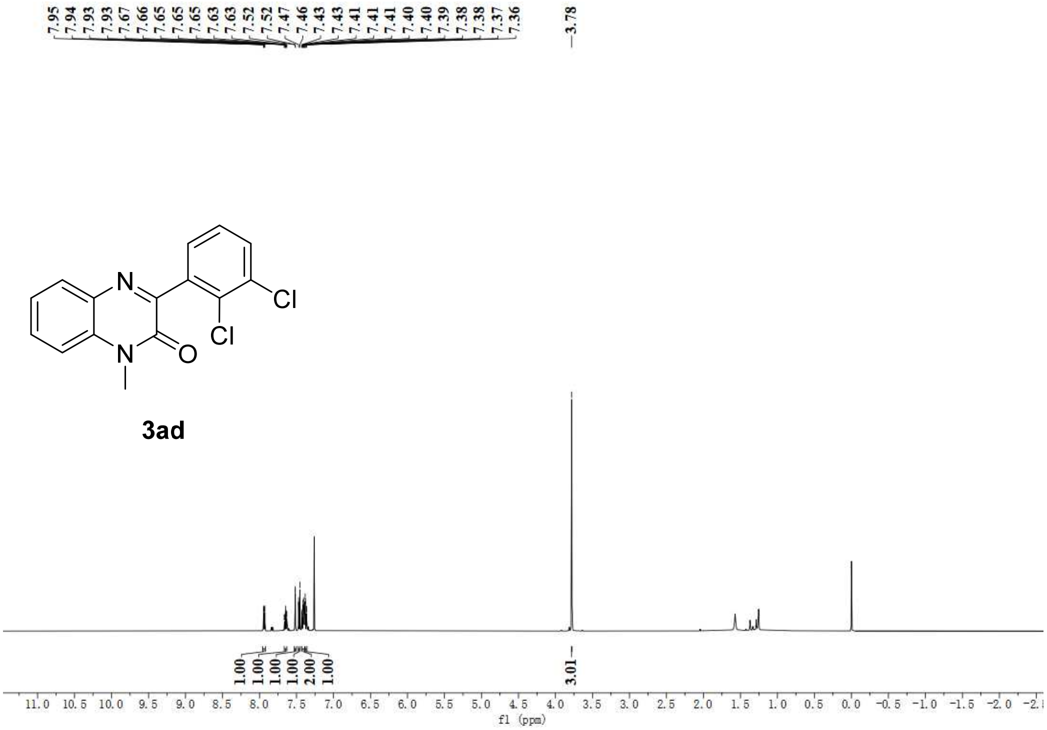

7.95 7.94 7.93 7.93 7.67 7.66 7.65 7.65 7.65 7.63 7.63 7.52 7.52 7.47 7.46 7.43 7.43 7.41 7.41 7.41 7.40 7.40 7.39 7.38 7.38 7.37 7.36
3.78
N
N
O
Cl
Cl
3ad
1.00 1.00 1.00 1.00 2.00 1.00
3.01
f1 (ppm)

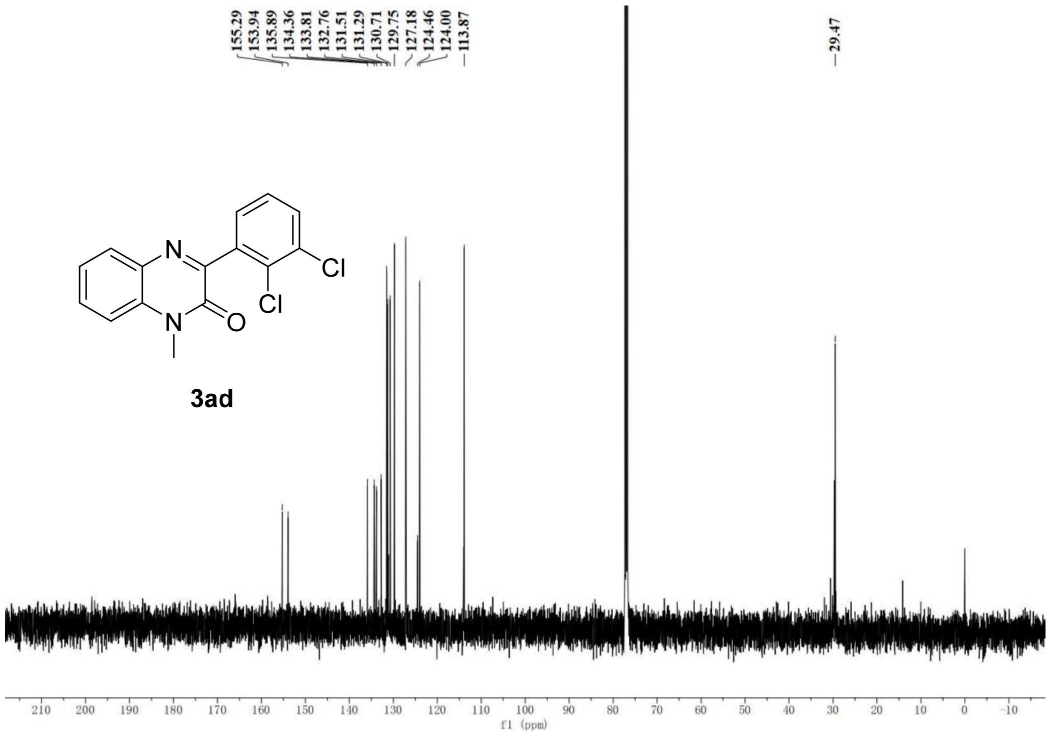
155.29
153.94
135.89
134.36
133.81
132.76
131.51
131.29
130.71
129.75
127.18
124.46
124.00
113.87
29.47
Cl
Cl
N
N
O
3ad
f1 (ppm)

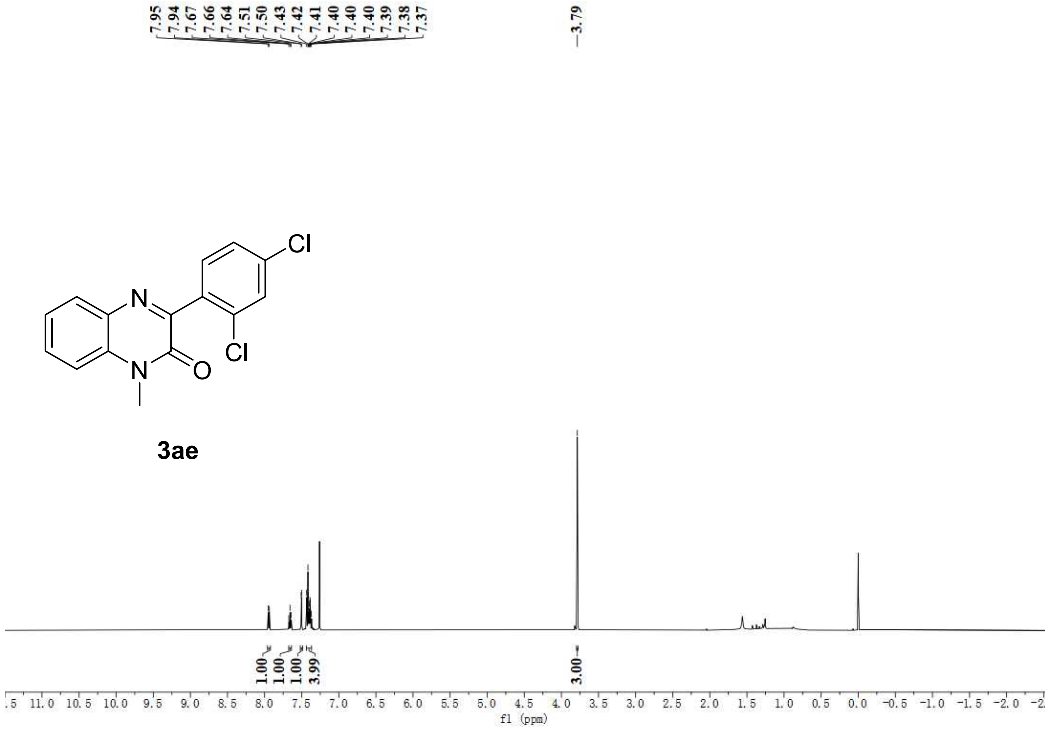
7.95
7.94
7.67
7.66
7.64
7.51
7.50
7.43
7.42
7.41
7.40
7.40
7.40
7.39
7.38
7.37
3.79
Cl
Cl
N
N
O
3ae
1.00
1.00
1.00
3.99
3.00
f1 (ppm)

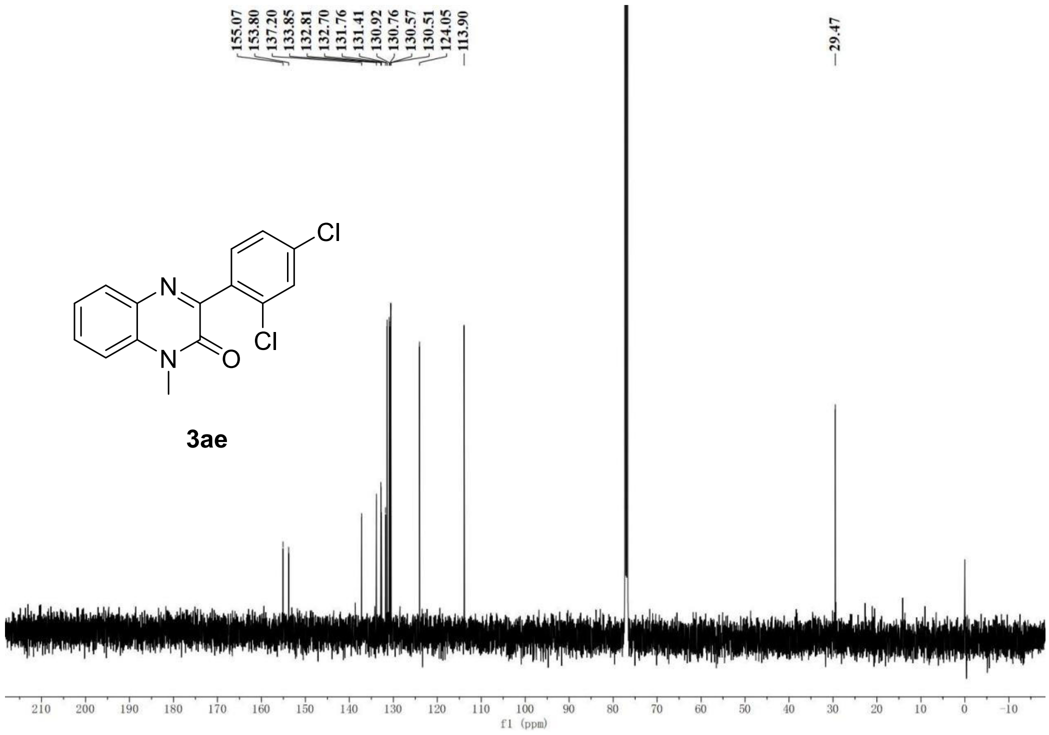
155.07
153.80
137.20
133.85
132.81
132.70
131.76
131.41
130.92
130.76
130.57
130.51
124.05
113.90
29.47
Cl
N
Cl
N
O
210 200 190 180 170 160 150 140 130 120 110 100 90 80 70 60 50 40 30 20 10 0 -10
f1 (ppm)

3ae

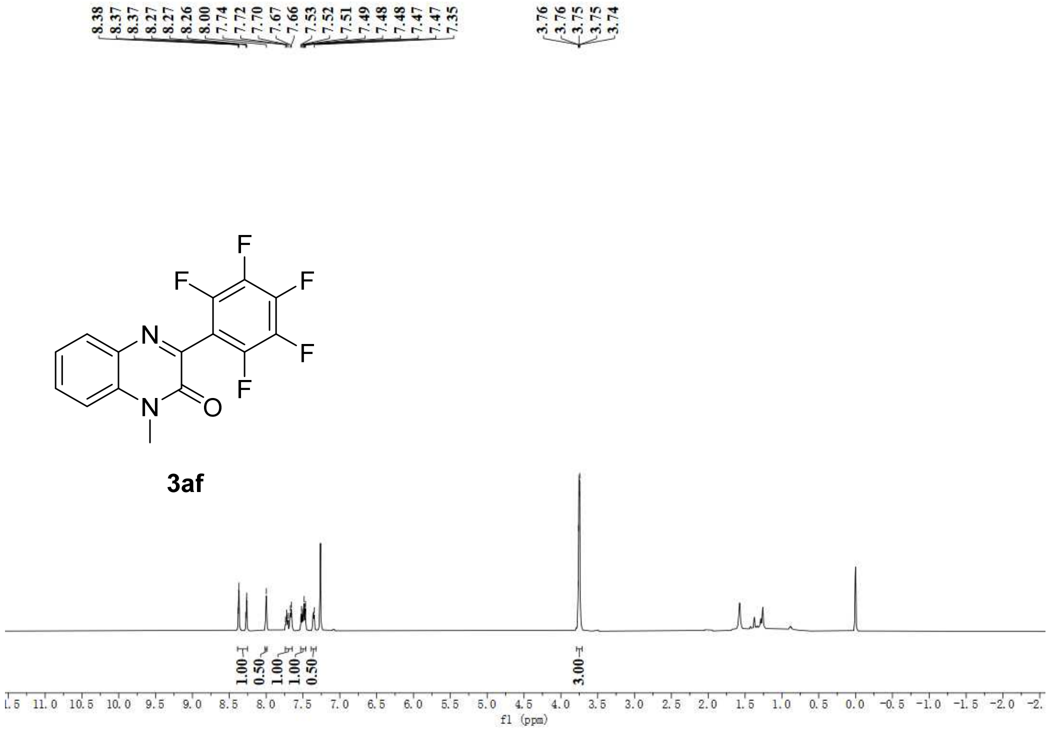
8.38
8.37
8.37
8.27
8.27
8.26
8.00
7.74
7.72
7.70
7.67
7.66
7.53
7.52
7.51
7.49
7.48
7.48
7.47
7.47
7.35
3.76
3.76
3.75
3.75
3.74
F
F
F
F
F
N
N
O
1.00
0.50
1.00
1.00
0.50
3.00
11.0 10.5 10.0 9.5 9.0 8.5 8.0 7.5 7.0 6.5 6.0 5.5 5.0 4.5 4.0 3.5 3.0 2.5 2.0 1.5 1.0 0.5 0.0 -0.5 -1.0 -1.5 -2.0
f1 (ppm)

3af

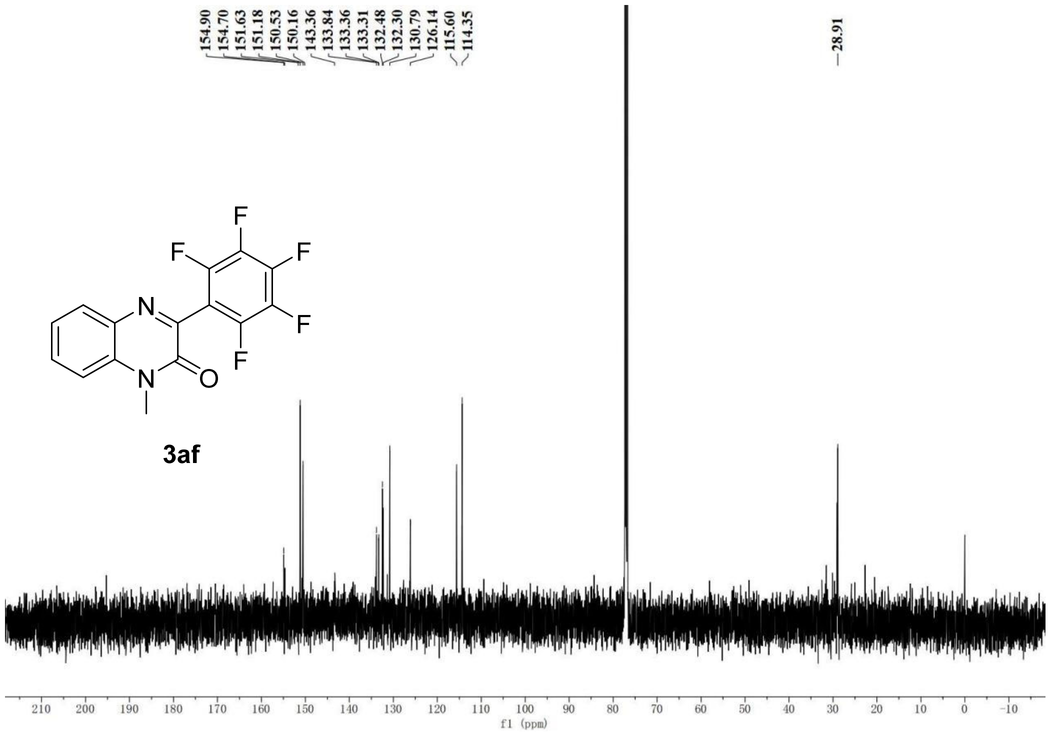
154.90
154.70
151.63
151.18
150.53
150.16
143.36
133.84
133.36
133.31
132.48
132.30
130.79
126.14
115.60
114.35
28.91
3af
f1 (ppm)

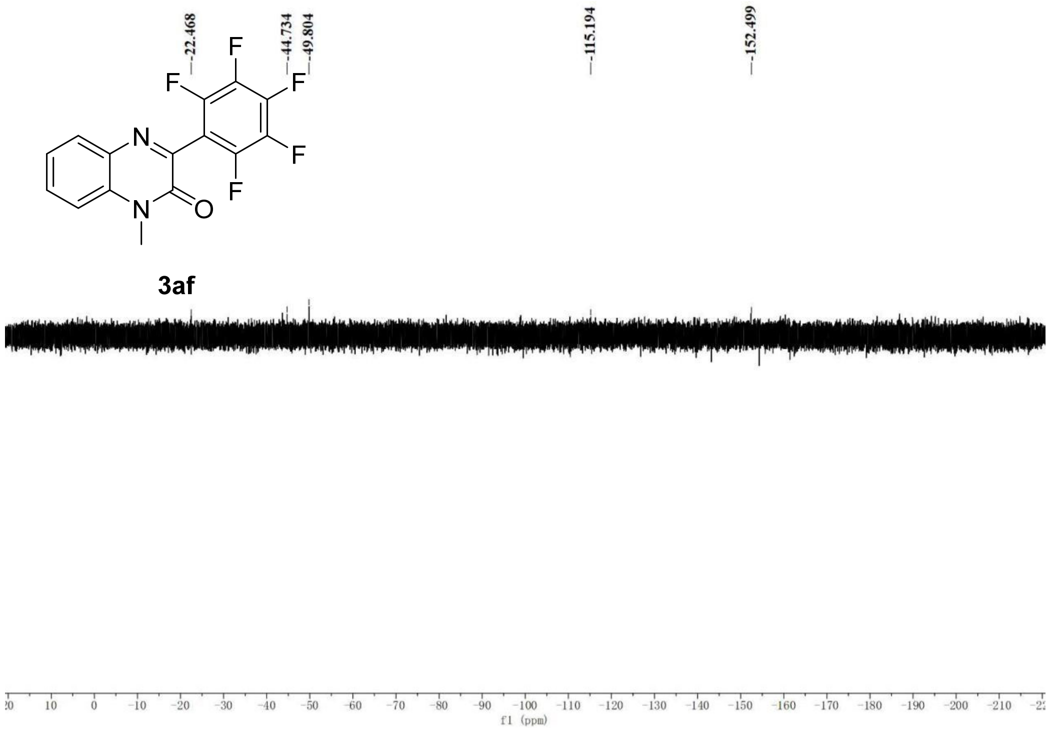
-22.468
-44.734
-49.804
-115.194
-152.499
3af
f1 (ppm)

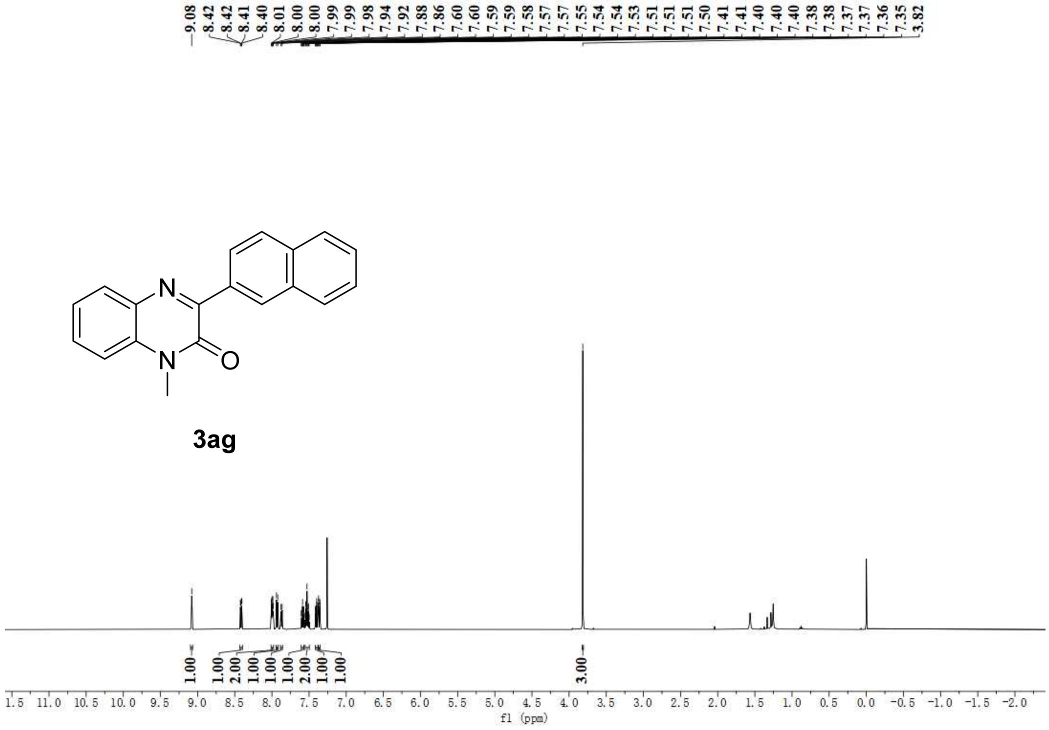
9.08
8.42
8.42
8.41
8.40
8.01
8.00
8.00
7.99
7.99
7.98
7.94
7.92
7.88
7.86
7.60
7.60
7.59
7.59
7.58
7.57
7.57
7.55
7.54
7.54
7.53
7.51
7.51
7.51
7.50
7.41
7.41
7.40
7.40
7.40
7.38
7.38
7.37
7.36
7.35
3.82
N
N
O
3ag
1.00
1.00
2.00
1.00
1.00
1.00
2.00
1.00
1.00
3.00
f1 (ppm)

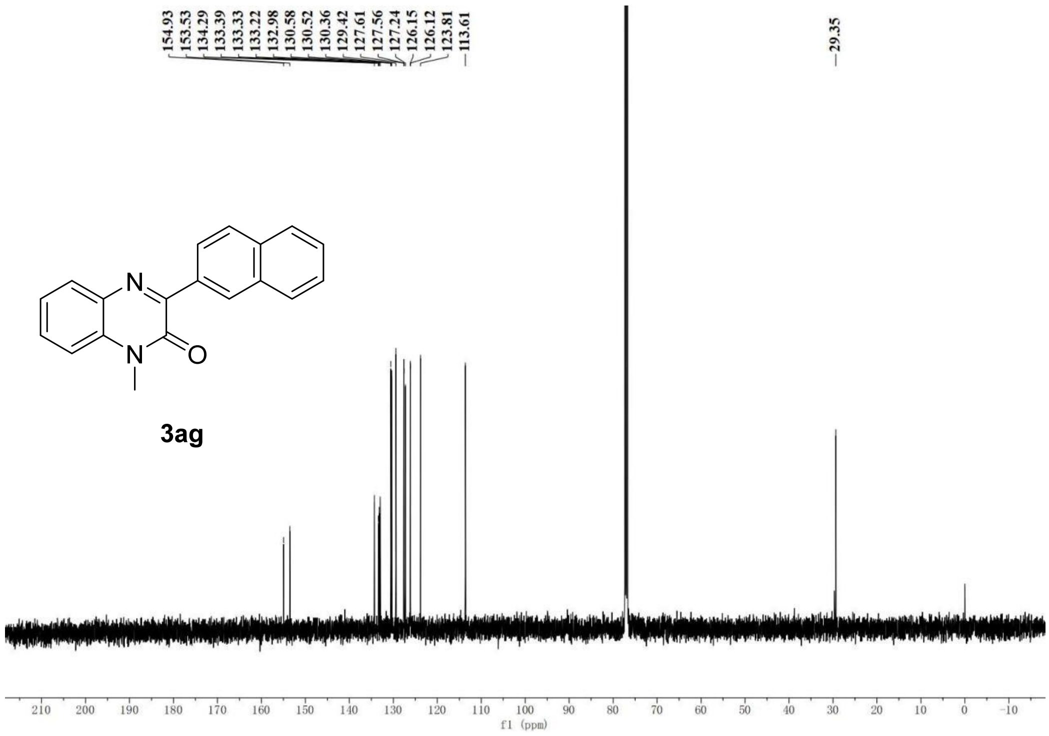
154.93
153.53
134.29
133.39
133.33
133.22
132.98
130.58
130.52
130.36
129.42
127.61
127.56
127.24
126.15
126.12
123.81
113.61
29.35
N
N
O
3ag
f1 (ppm)

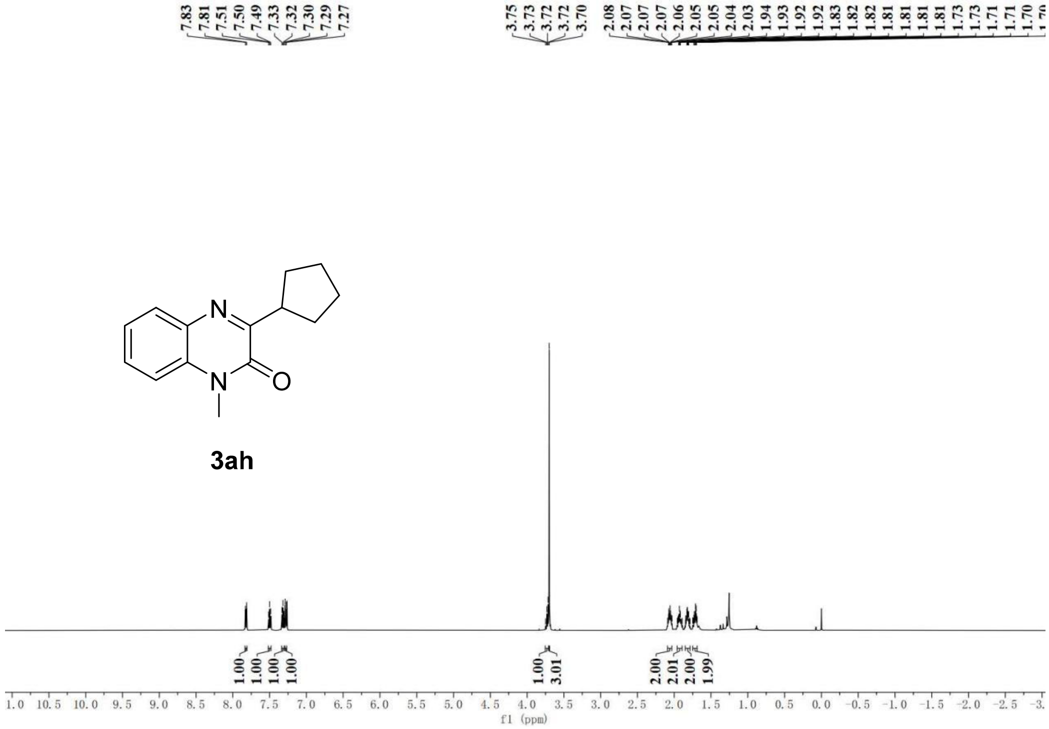
7.83
7.81
7.51
7.50
7.49
7.33
7.32
7.30
7.29
7.27
3.75
3.73
3.72
3.72
3.70
2.08
2.07
2.07
2.07
2.06
2.05
2.05
2.04
2.03
1.94
1.93
1.92
1.92
1.83
1.82
1.82
1.81
1.81
1.81
1.81
1.73
1.73
1.71
1.71
1.70
N
N
O
3ah
1.00
1.00
1.00
1.00
1.00
3.01
2.00
2.01
2.00
1.99
f1 (ppm)

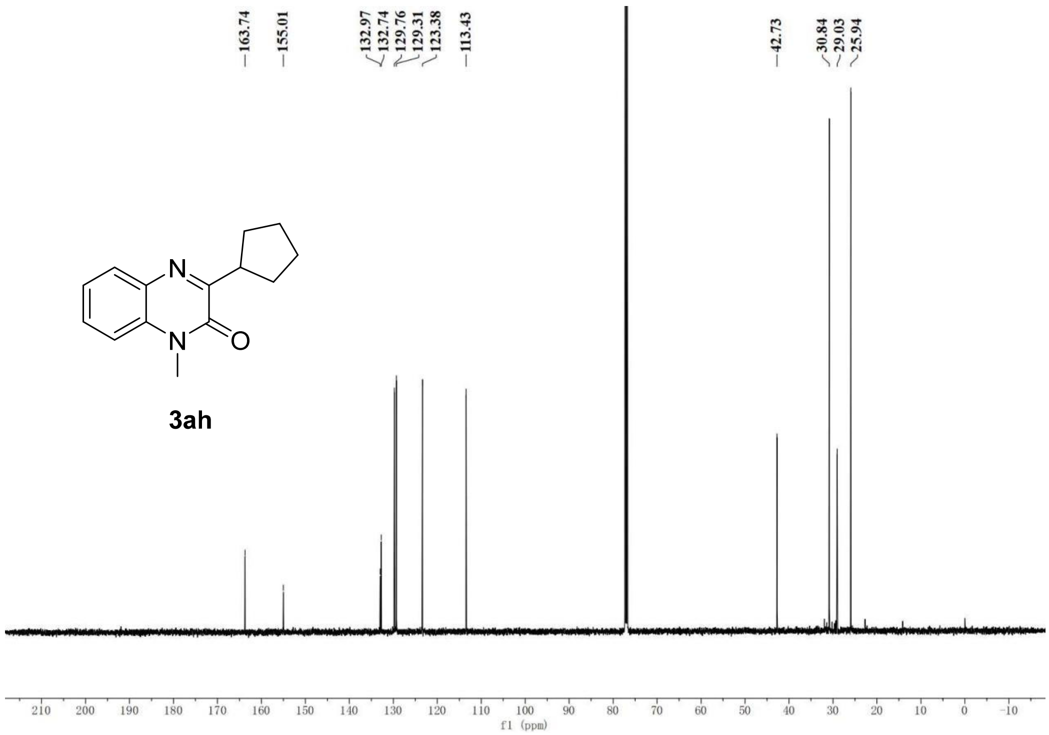
163.74
155.01
132.97
132.74
129.76
129.31
123.38
113.43
42.73
30.84
29.03
25.94
N
N
O
3ah
f1 (ppm)

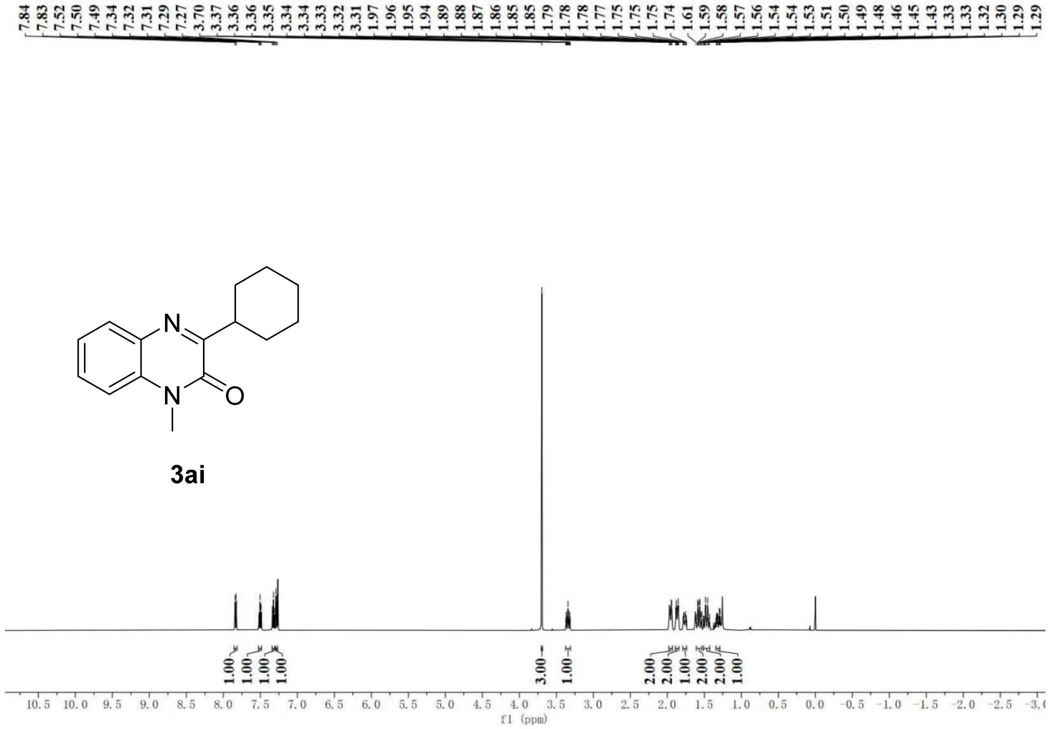
7.84 7.83 7.52 7.50 7.49 7.34 7.32 7.31 7.29 7.27 3.70 3.37 3.36 3.36 3.35 3.34 3.34 3.33 3.32 3.31 1.97 1.96 1.95 1.94 1.89 1.88 1.87 1.86 1.85 1.85 1.79 1.78 1.78 1.77 1.75 1.75 1.75 1.74 1.61 1.59 1.58 1.57 1.56 1.54 1.54 1.53 1.51 1.50 1.49 1.48 1.46 1.45 1.43 1.33 1.33 1.32 1.30 1.29 1.29
3ai
1.00 1.00 1.00 1.00 3.00 1.00 2.00 2.00 1.00 2.00 2.00 1.00
f1 (ppm)

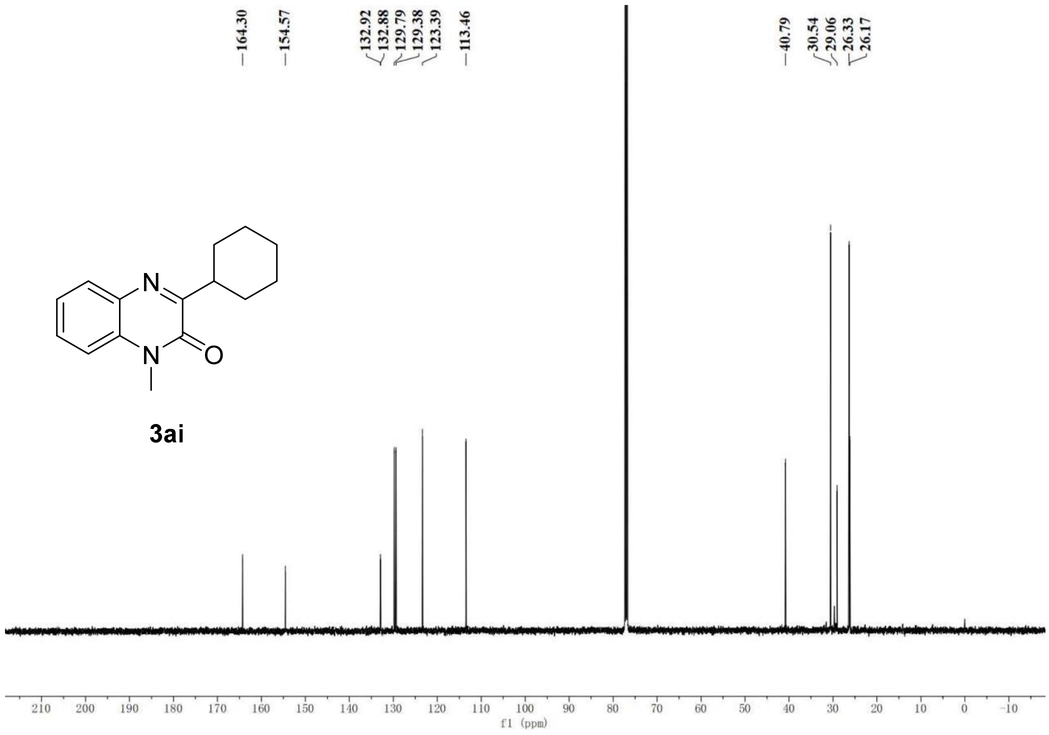
164.30 154.57 132.92 132.88 129.79 129.38 123.39 113.46 40.79 30.54 29.06 26.33 26.17
3ai
f1 (ppm)

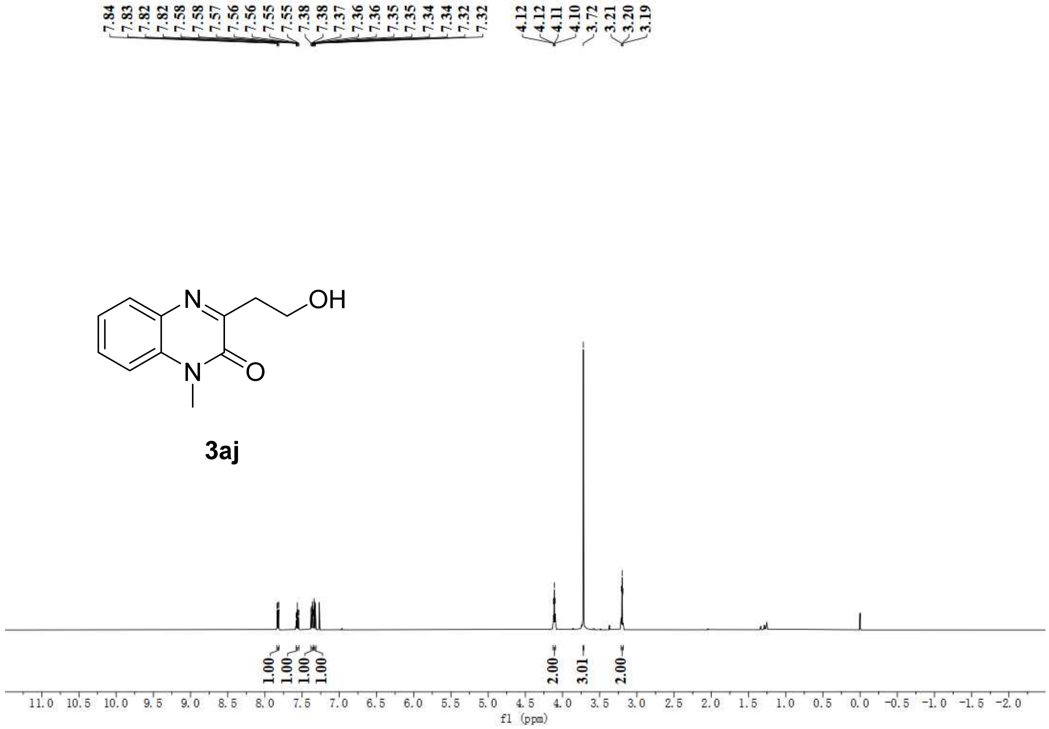
7.84 7.83 7.82 7.82 7.58 7.58 7.57 7.56 7.56 7.55 7.55 7.38 7.38 7.37 7.36 7.36 7.35 7.35 7.34 7.34 7.32 7.32
4.12 4.12 4.11 4.10 3.72 3.21 3.20 3.19
N
OH
N
O
3aj
1.00 1.00 1.00 1.00
2.00 3.01 2.00
f1 (ppm)

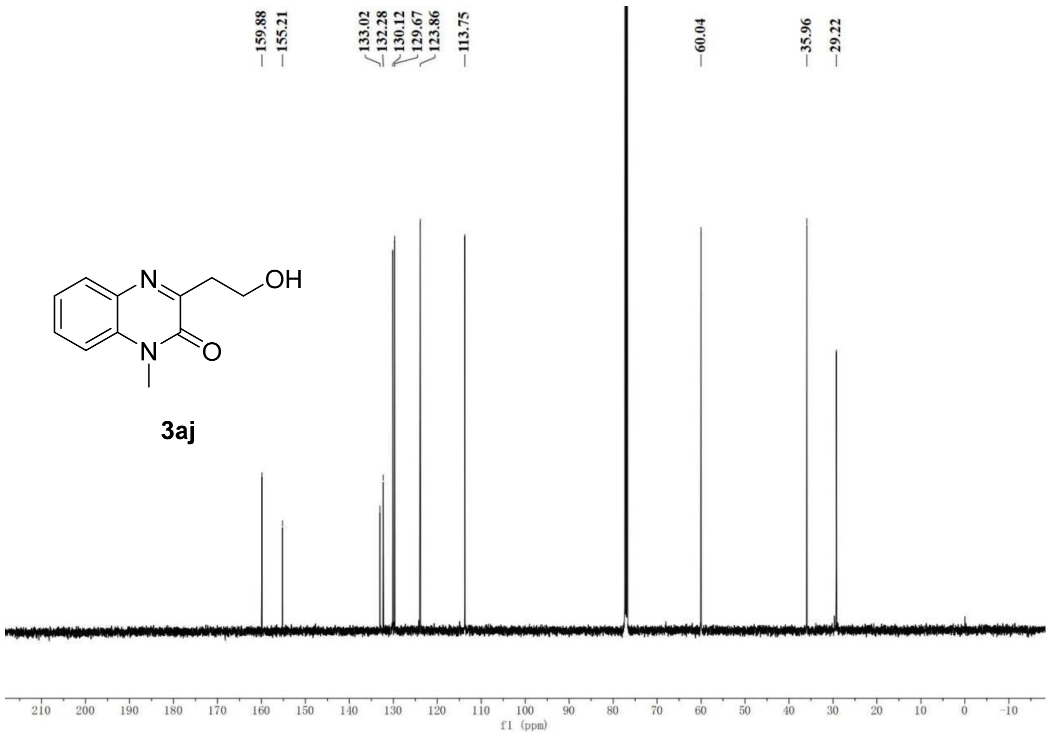
159.88 155.21
133.02 132.28 130.12 129.67 123.86 113.75
60.04
35.96 29.22
N
OH
N
O
3aj
f1 (ppm)

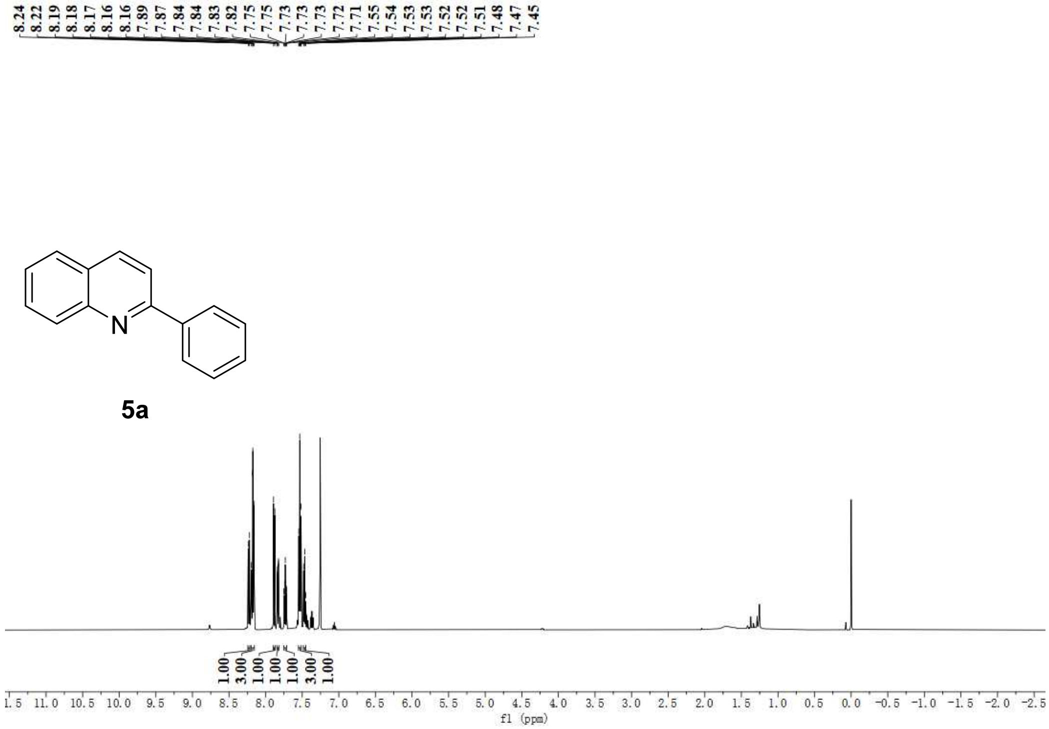
8.24
8.22
8.19
8.18
8.17
8.16
8.16
7.89
7.87
7.84
7.84
7.83
7.82
7.75
7.75
7.73
7.73
7.73
7.72
7.71
7.55
7.54
7.53
7.53
7.52
7.52
7.51
7.48
7.47
7.45
N
5a
1.00
3.00
1.00
1.00
1.00
3.00
1.00
f1 (ppm)

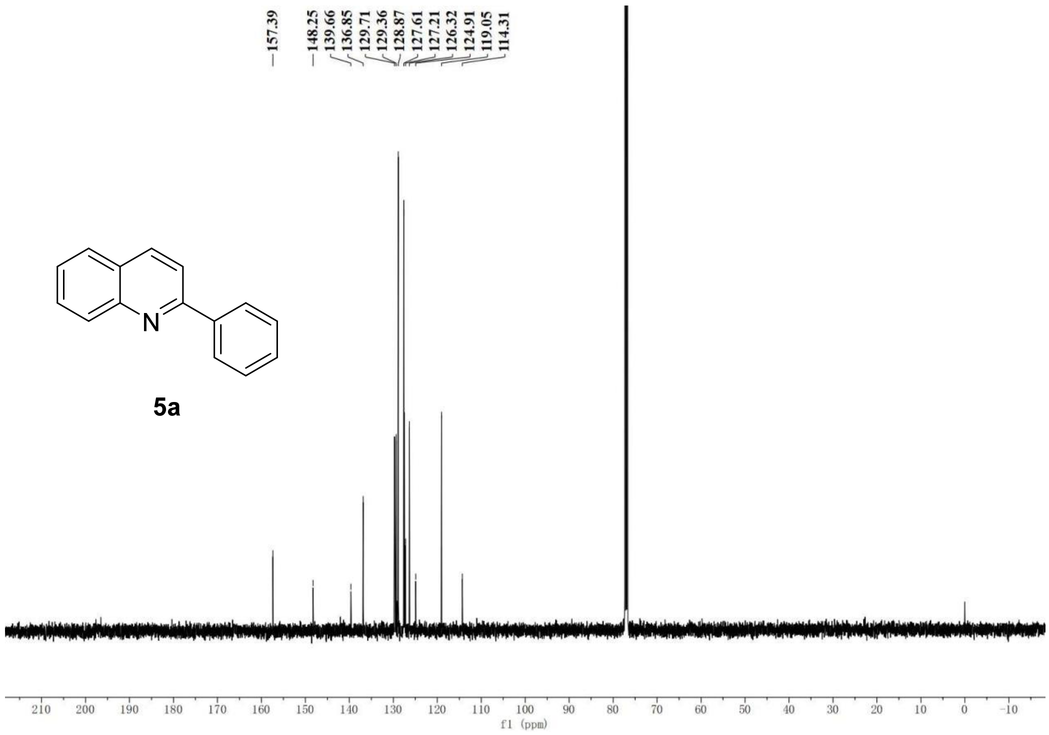
157.39
148.25
139.66
136.85
129.71
129.36
128.87
127.61
127.21
126.32
124.91
119.05
114.31
N
5a
f1 (ppm)

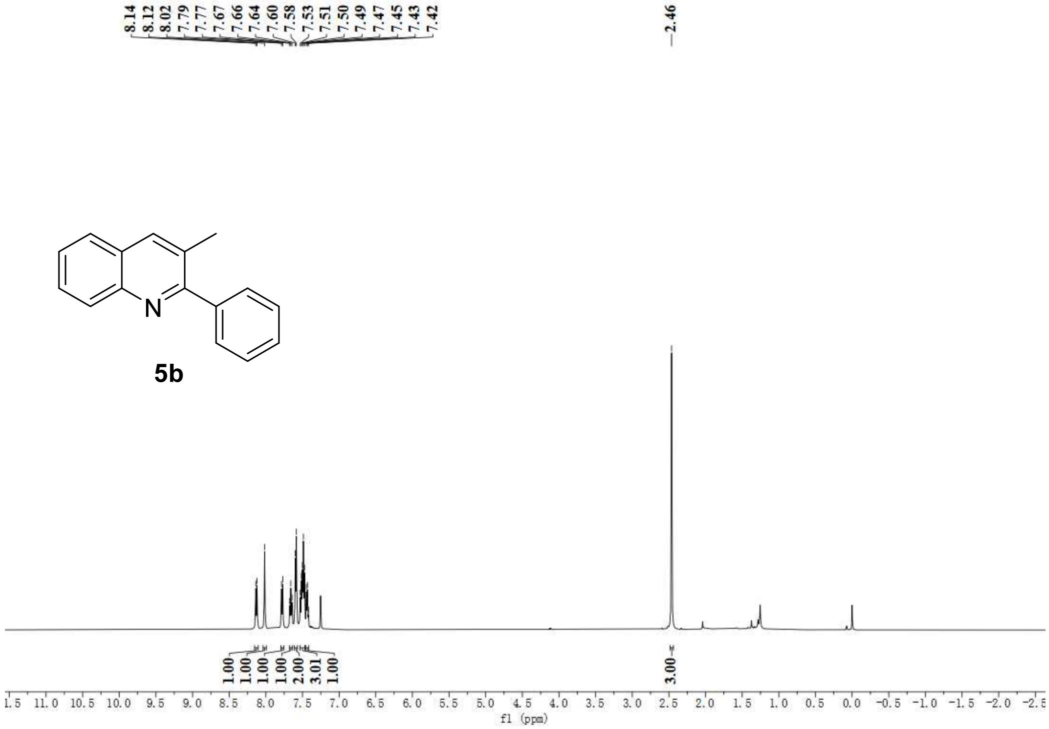
8.14
8.12
8.02
7.79
7.77
7.67
7.66
7.64
7.60
7.58
7.53
7.51
7.50
7.49
7.47
7.45
7.43
7.42
2.46
N
5b
1.00
1.00
1.00
1.00
2.00
3.01
1.00
3.00
f1 (ppm)

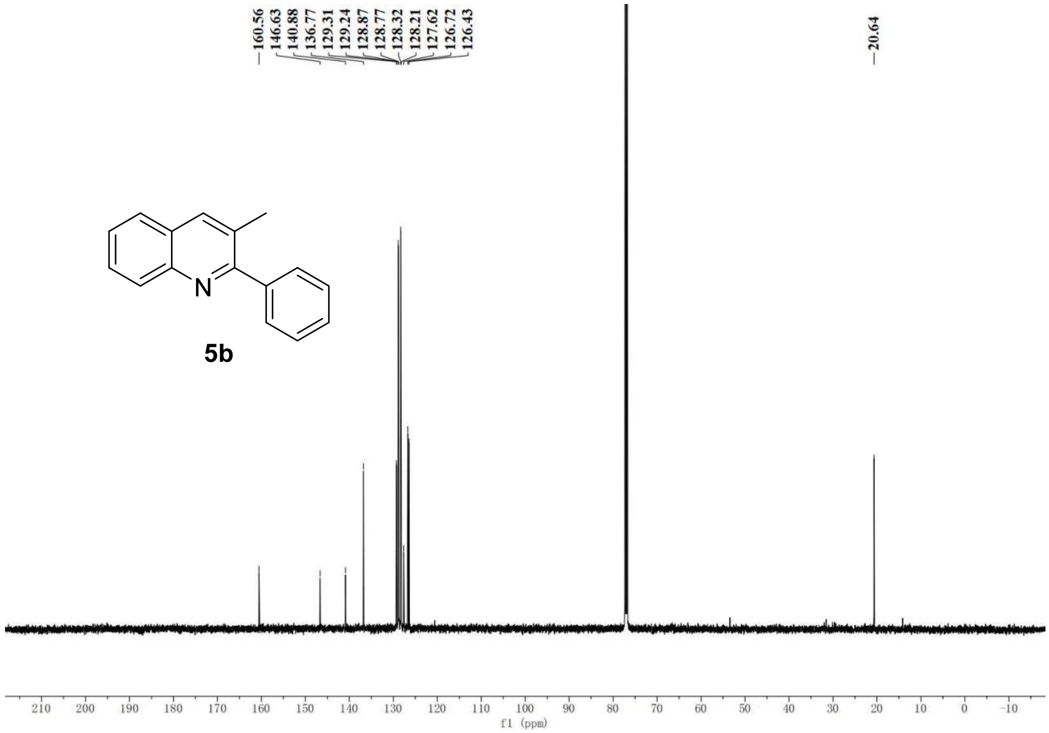
160.56
146.63
140.88
136.77
129.31
129.24
128.87
128.77
128.32
128.21
127.62
126.72
126.43
20.64
N
5b
f1 (ppm)

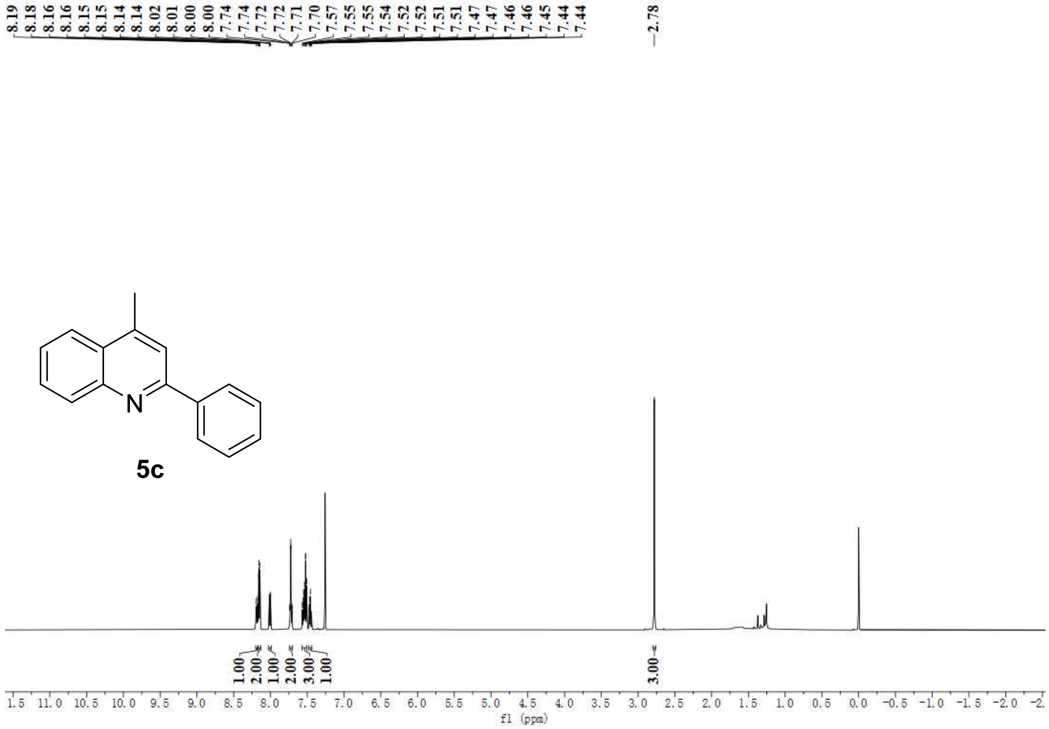
8.19
8.18
8.16
8.16
8.15
8.15
8.14
8.14
8.02
8.01
8.00
8.00
7.74
7.74
7.72
7.72
7.71
7.70
7.57
7.55
7.55
7.54
7.52
7.52
7.51
7.51
7.47
7.47
7.46
7.46
7.45
7.44
7.44
2.78
N
5c
1.00
2.00
1.00
2.00
3.00
1.00
3.00
f1 (ppm)

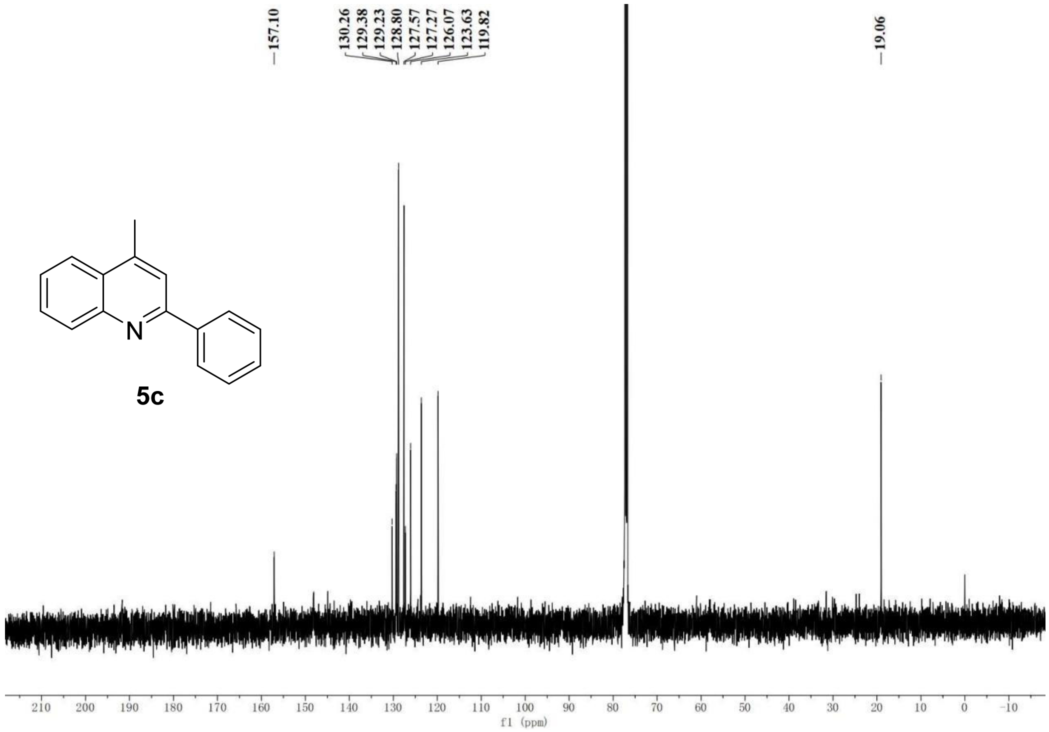
157.10
130.26
129.38
129.23
128.80
127.57
127.27
126.07
123.63
119.82
19.06
N
5c
f1 (ppm)

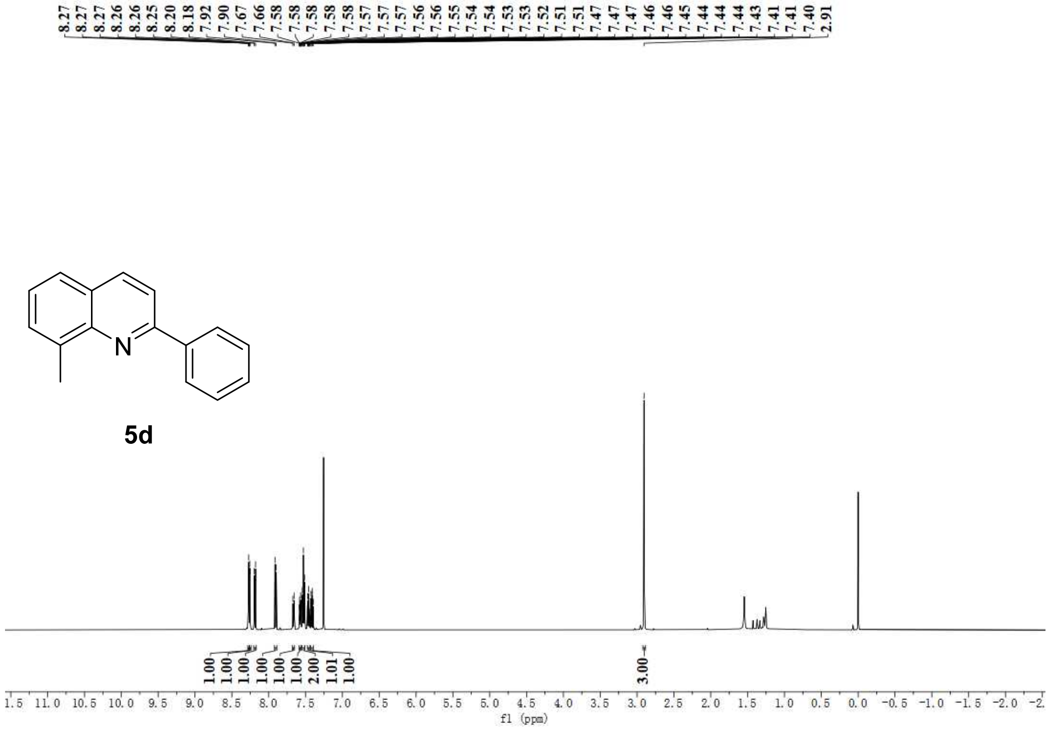

8.27 8.27 8.27 8.26 8.26 8.25 8.20 8.18 7.92 7.90 7.67 7.66 7.58 7.58 7.58 7.58 7.58 7.57 7.57 7.57 7.56 7.56 7.55 7.54 7.54 7.53 7.53 7.52 7.51 7.51 7.47 7.47 7.47 7.46 7.46 7.45 7.44 7.44 7.44 7.43 7.41 7.41 7.40 2.91
N
1.00 1.00 1.00 1.00 1.00 2.00 1.01 1.00
3.00
f1 (ppm)

5d

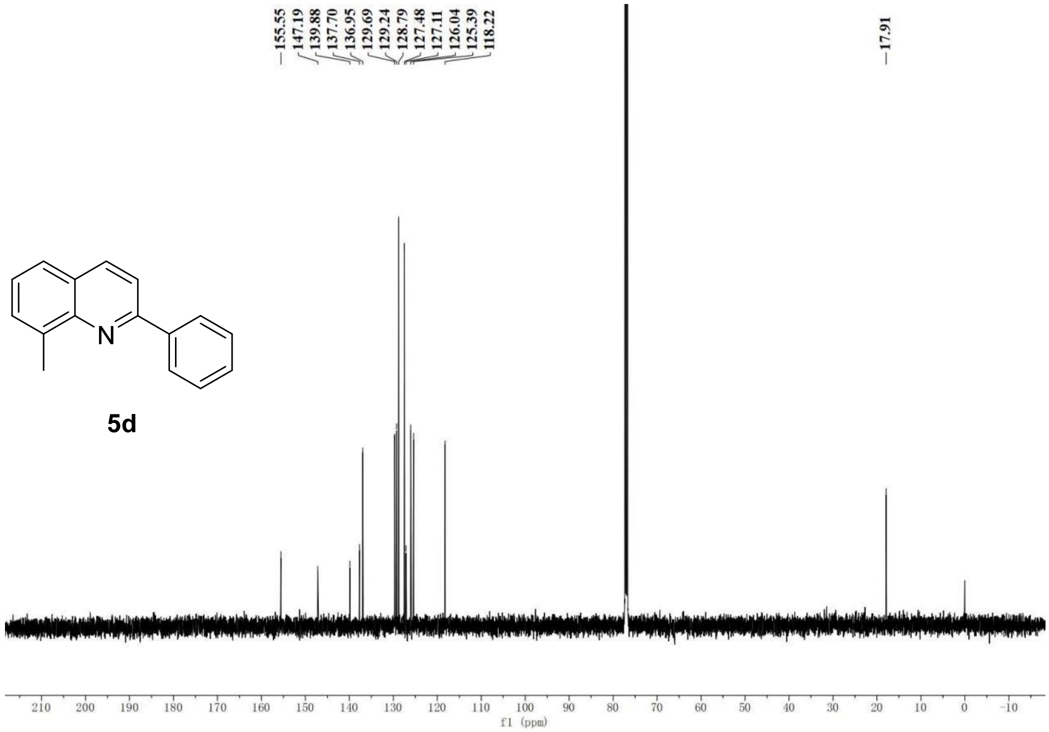

155.55 147.19 139.88 137.70 136.95 129.69 129.24 128.79 127.48 127.11 126.04 125.39 118.22
17.91
N
f1 (ppm)

5d

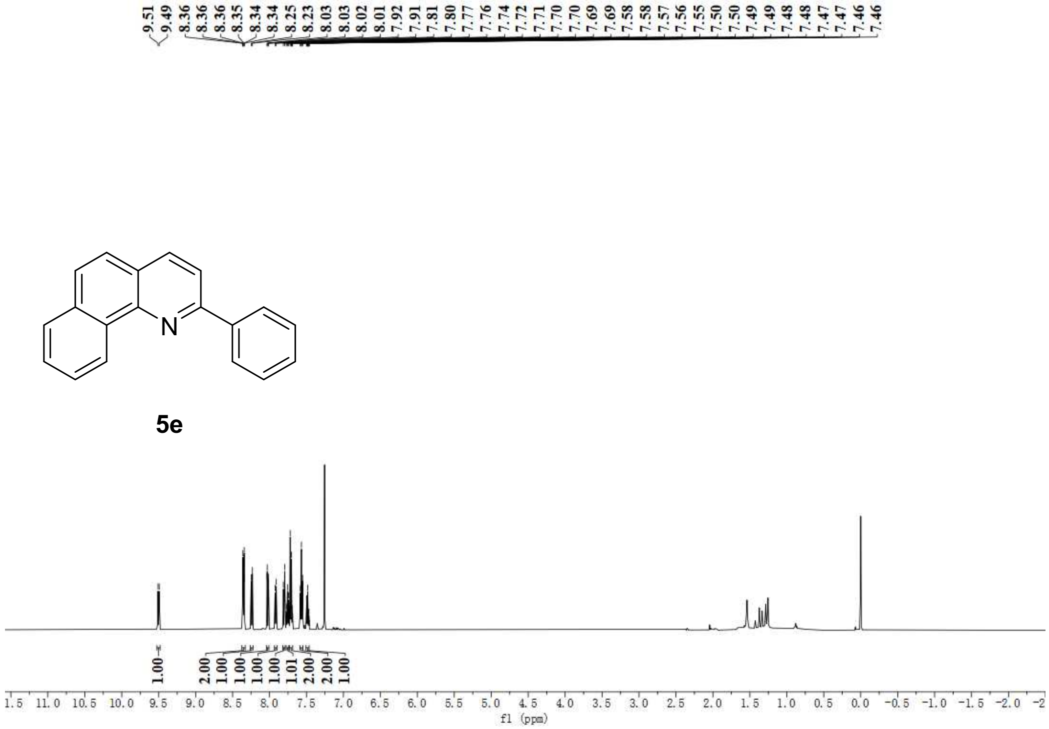
9.51 9.49 8.36 8.36 8.36 8.35 8.34 8.34 8.25 8.23 8.03 8.03 8.02 8.01 7.92 7.91 7.81 7.80 7.77 7.76 7.74 7.72 7.71 7.70 7.70 7.69 7.69 7.58 7.58 7.57 7.56 7.55 7.50 7.50 7.49 7.49 7.48 7.47 7.47 7.46 7.46
1.00 2.00 1.00 1.00 1.00 1.00 1.01 2.00 2.00 1.00
N
f1 (ppm)

5e

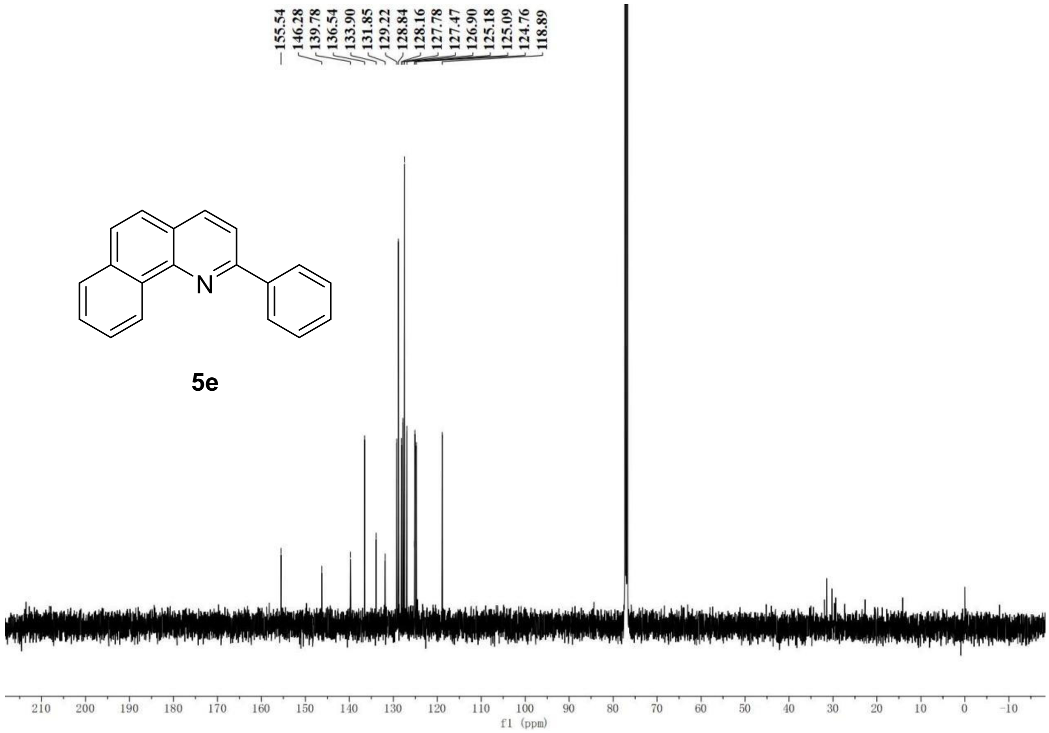
155.54 146.28 139.78 136.54 133.90 131.85 129.22 128.84 128.16 127.78 127.47 126.90 125.18 125.09 124.76 118.89
N
f1 (ppm)

5e

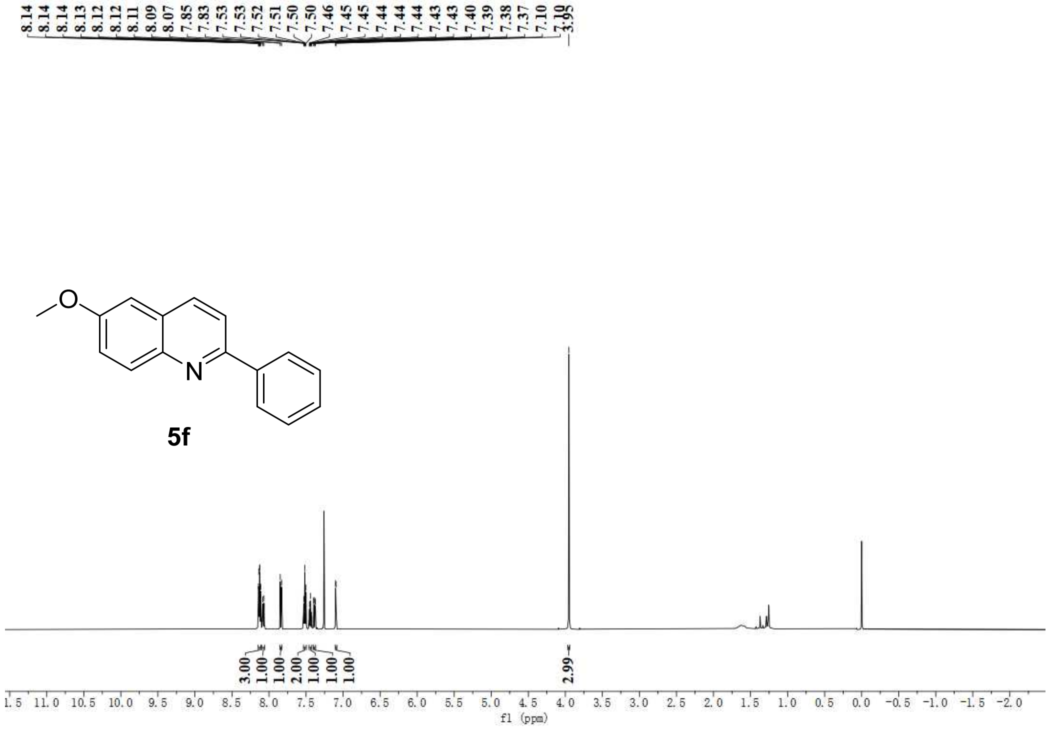

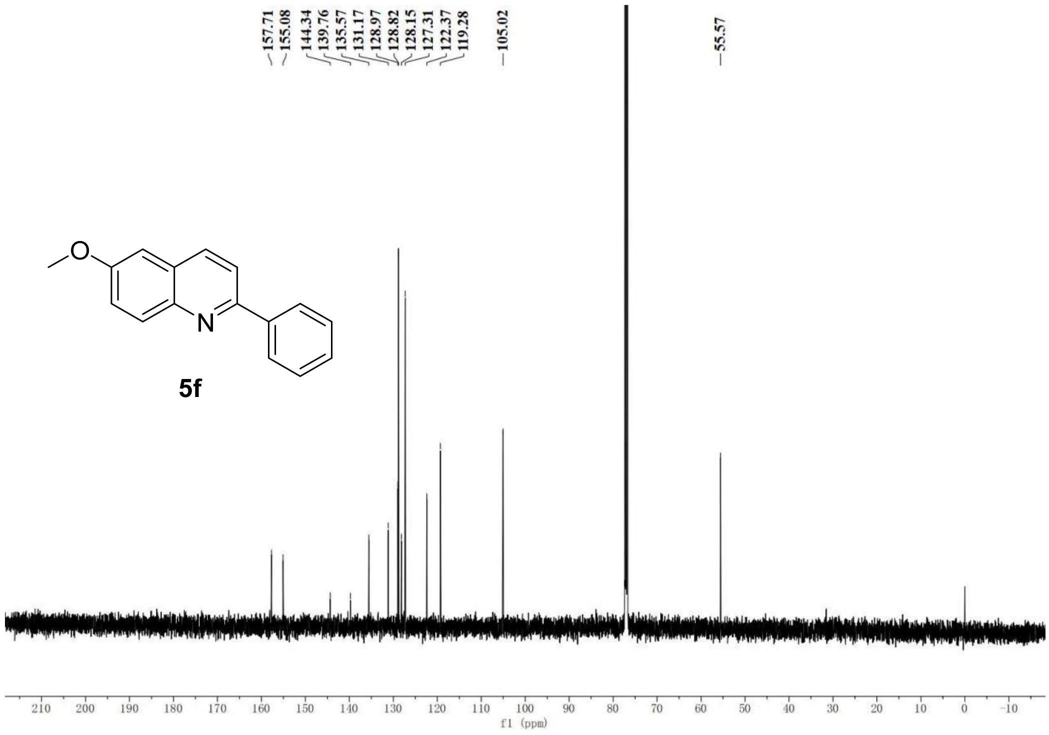

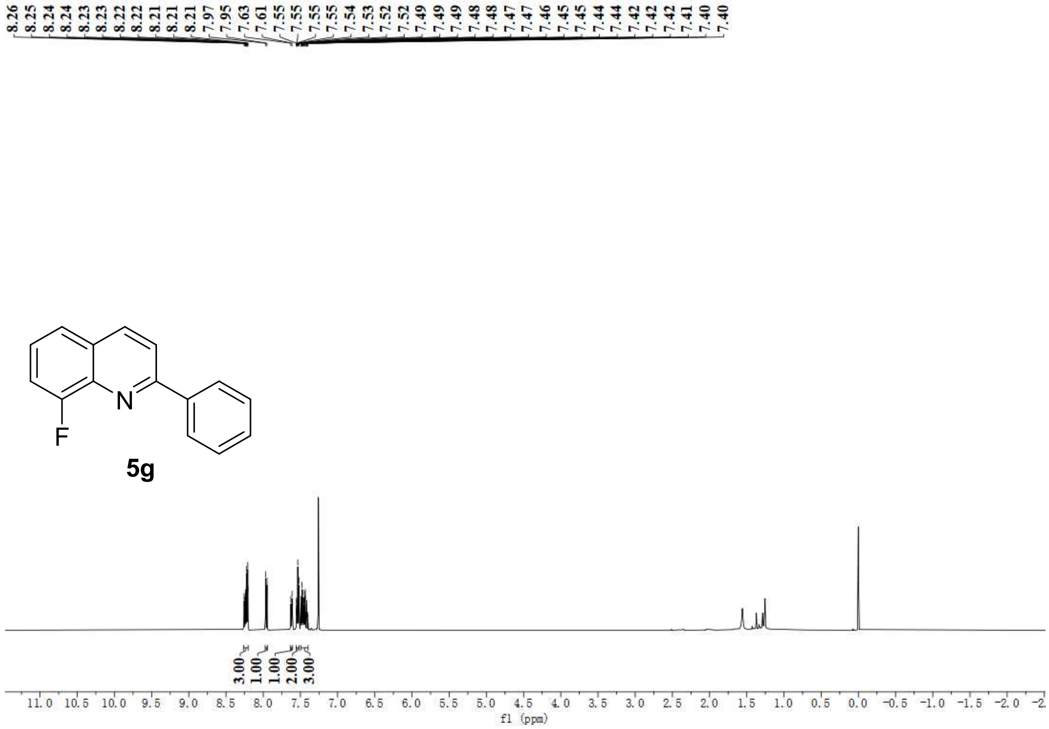
8.26
8.25
8.24
8.24
8.23
8.23
8.22
8.22
8.21
8.21
8.21
7.97
7.95
7.63
7.61
7.55
7.55
7.55
7.55
7.54
7.53
7.52
7.52
7.49
7.49
7.49
7.48
7.48
7.47
7.47
7.46
7.45
7.45
7.44
7.44
7.42
7.42
7.42
7.41
7.40
7.40
N
F
5g
3.00
1.00
1.00
2.00
3.00
f1 (ppm)

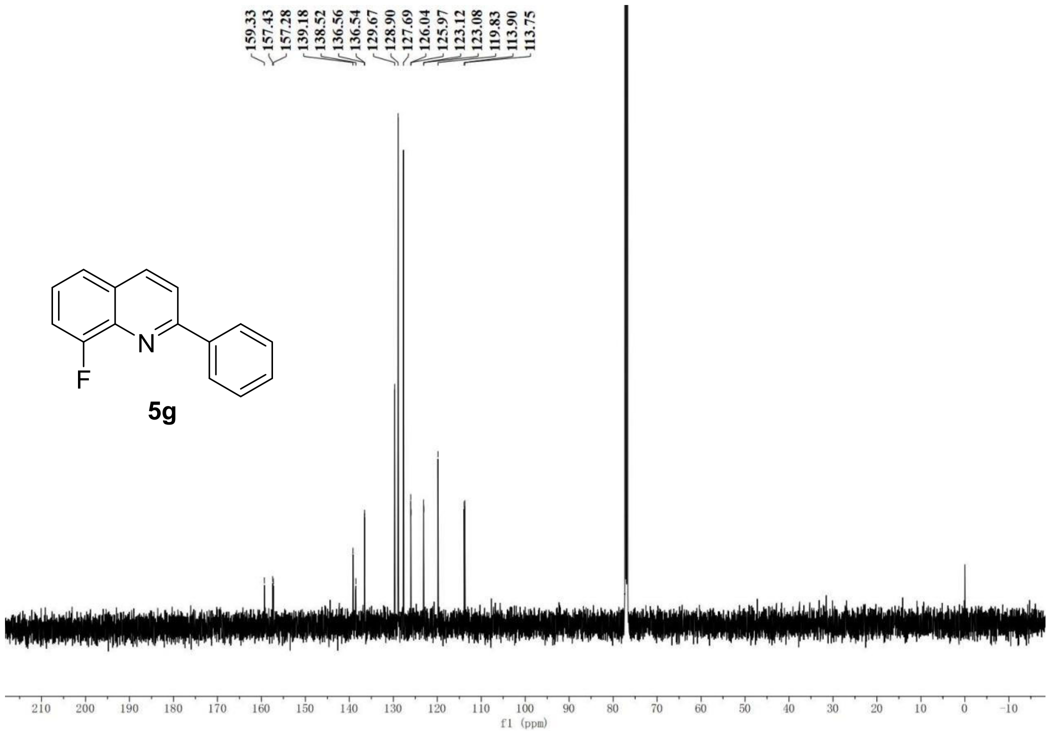
159.33
157.43
157.28
139.18
138.52
136.56
136.54
129.67
128.90
127.69
126.04
125.97
123.12
123.08
119.83
113.90
113.75
N
F
5g
f1 (ppm)

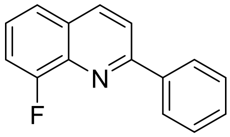

F
N

5g

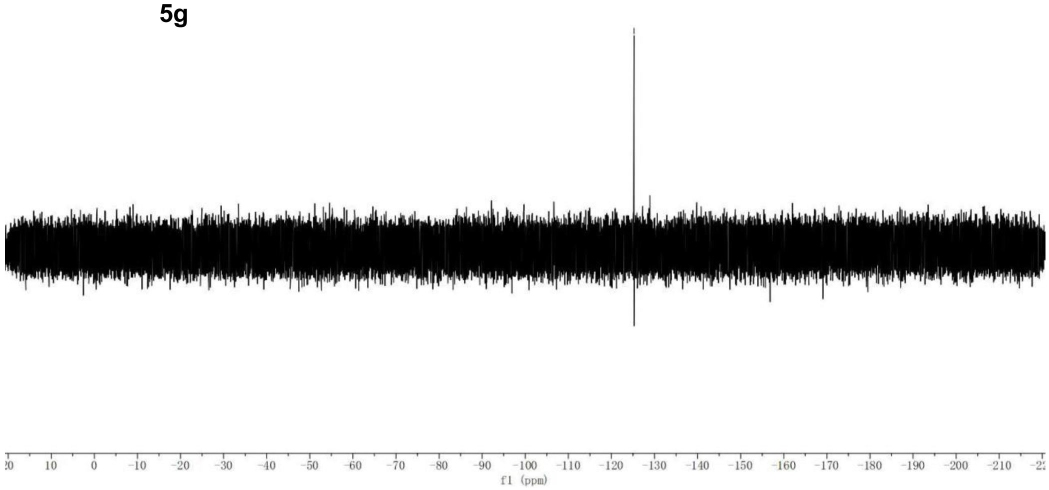

-125.26
f1 (ppm)

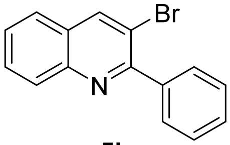

Br
N

5h

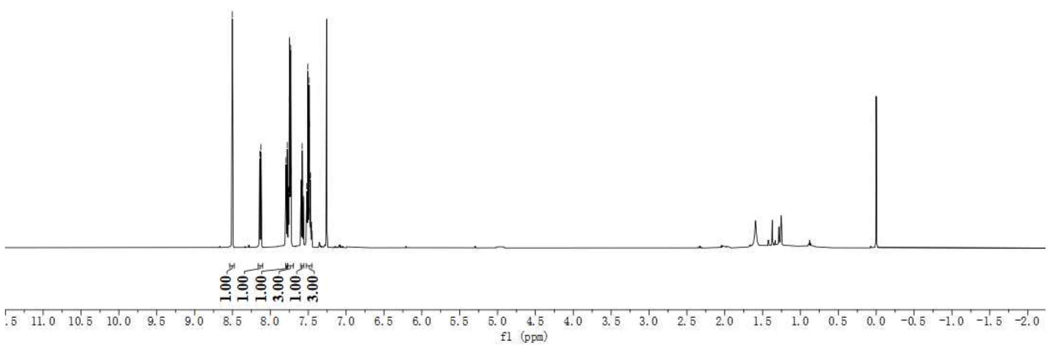

8.50 8.14 8.12 7.79 7.78 7.76 7.76 7.75 7.75 7.75 7.74 7.74 7.73 7.73 7.73 7.60 7.59 7.58 7.58 7.58 7.57 7.56 7.52 7.52 7.51 7.50 7.50 7.49 7.49 7.48 7.48 7.48 7.47 7.46 7.46 7.45 7.45
1.00 1.00 1.00 3.00 1.00 3.00
f1 (ppm)

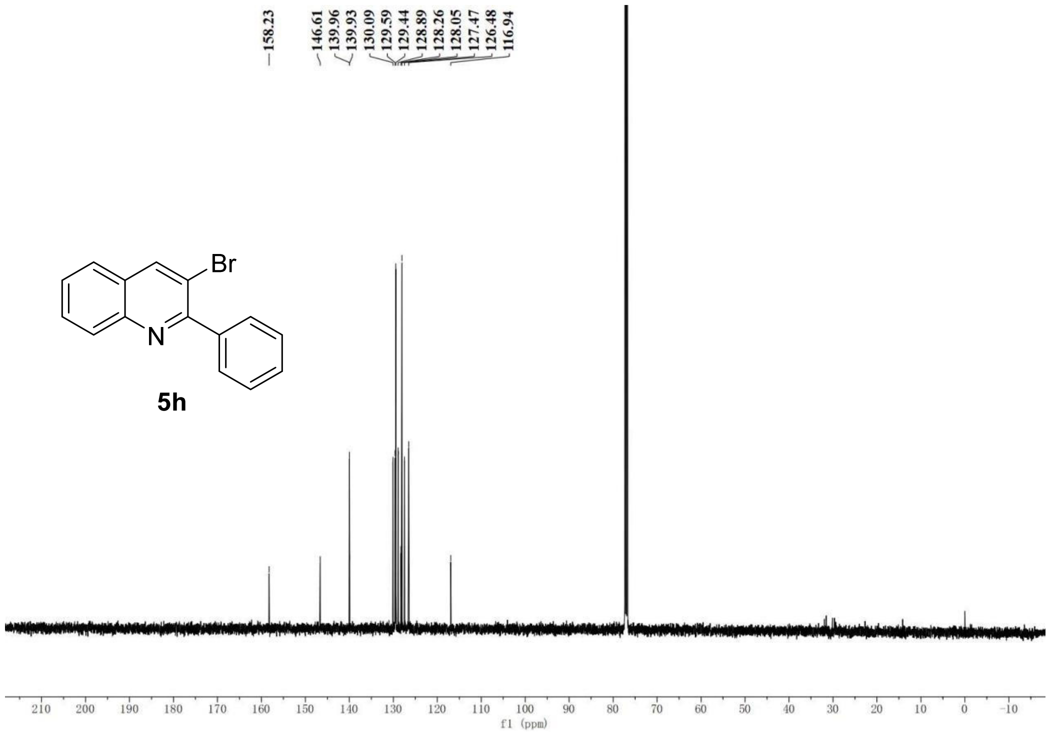
158.23
146.61
139.96
139.93
130.09
129.59
129.44
128.89
128.26
128.05
127.47
126.48
116.94
Br
N
5h
210 200 190 180 170 160 150 140 130 120 110 100 90 80 70 60 50 40 30 20 10 0 -10
f1 (ppm)

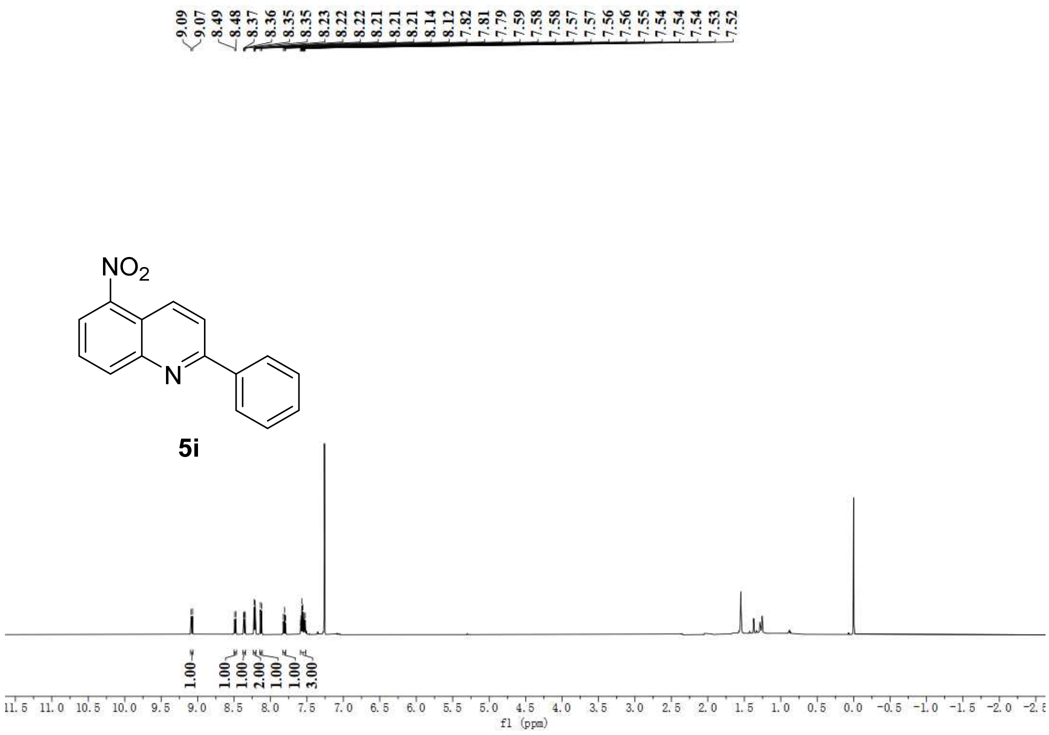
9.09
9.07
8.49
8.48
8.37
8.36
8.35
8.35
8.23
8.22
8.22
8.21
8.21
8.21
8.14
8.12
7.82
7.81
7.79
7.59
7.58
7.58
7.57
7.56
7.56
7.55
7.54
7.54
7.54
7.53
7.52
NO_2
N
5i
1.00
1.00
1.00
2.00
1.00
1.00
3.00
11.5 11.0 10.5 10.0 9.5 9.0 8.5 8.0 7.5 7.0 6.5 6.0 5.5 5.0 4.5 4.0 3.5 3.0 2.5 2.0 1.5 1.0 0.5 0.0 -0.5 -1.0 -1.5 -2.0
f1 (ppm)

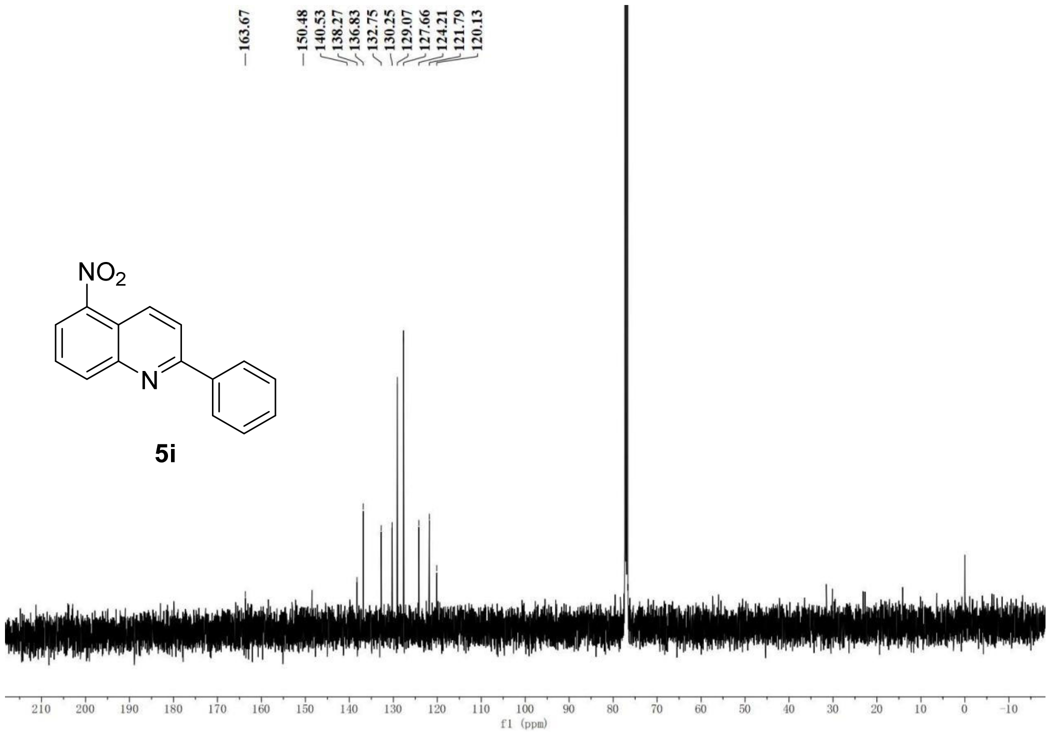

163.67
150.48
140.53
138.27
136.83
132.75
130.25
129.07
127.66
124.21
121.79
120.13
NO2
N
5i
210 200 190 180 170 160 150 140 130 120 110 100 90 80 70 60 50 40 30 20 10 0 -10
f1 (ppm)

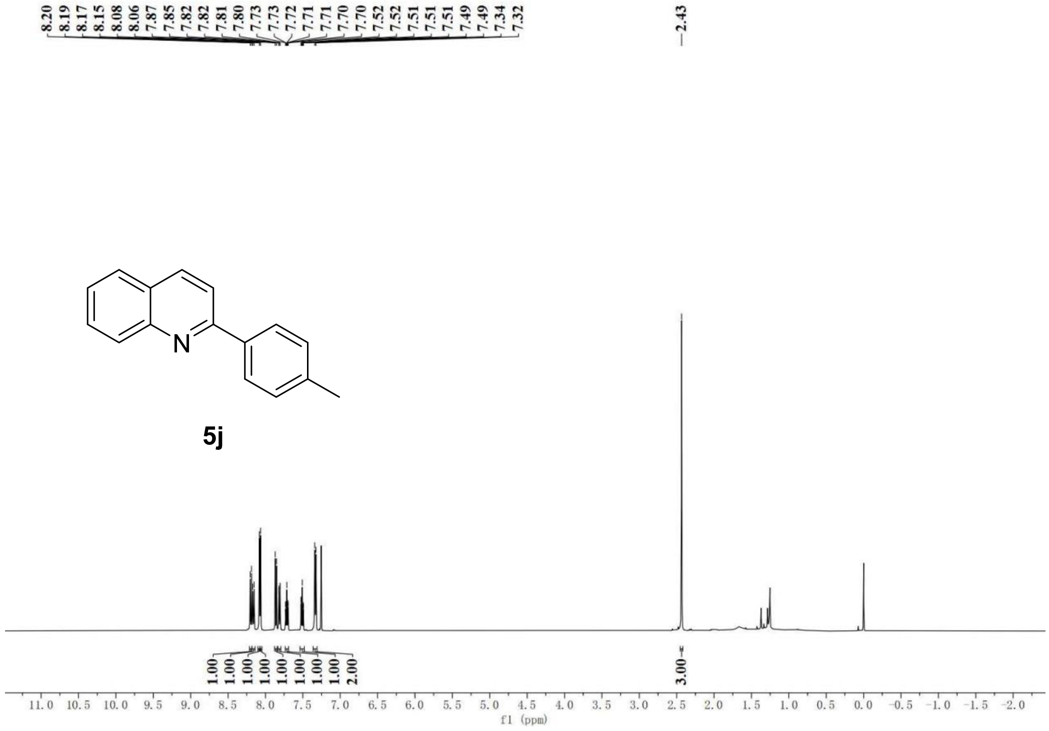

8.20
8.19
8.17
8.15
8.08
8.06
7.87
7.85
7.82
7.82
7.81
7.80
7.73
7.73
7.72
7.71
7.71
7.70
7.70
7.52
7.52
7.51
7.51
7.51
7.49
7.34
7.32
2.43
N
5j
1.00
1.00
1.00
1.00
1.00
1.00
1.00
2.00
3.00
11.0 10.5 10.0 9.5 9.0 8.5 8.0 7.5 7.0 6.5 6.0 5.5 5.0 4.5 4.0 3.5 3.0 2.5 2.0 1.5 1.0 0.5 0.0 -0.5 -1.0 -1.5 -2.0
f1 (ppm)

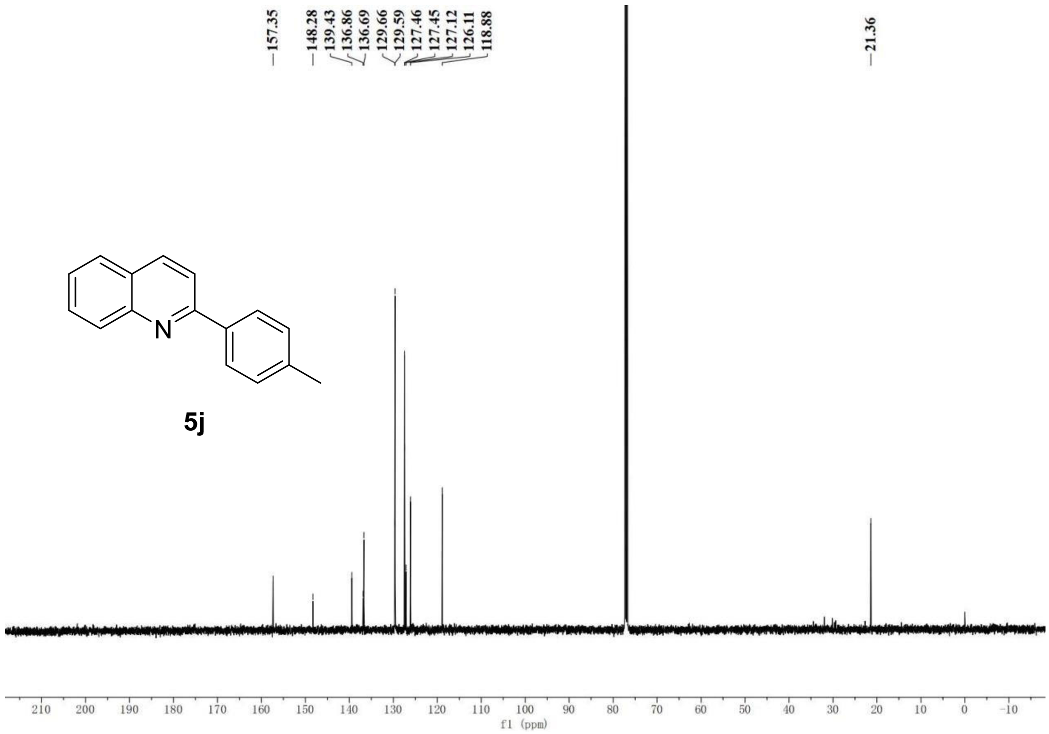
157.35
148.28
139.43
136.86
136.69
129.66
129.59
127.46
127.45
127.12
126.11
118.88
21.36
N
5j
210 200 190 180 170 160 150 140 130 120 110 100 90 80 70 60 50 40 30 20 10 0 -10
f1 (ppm)

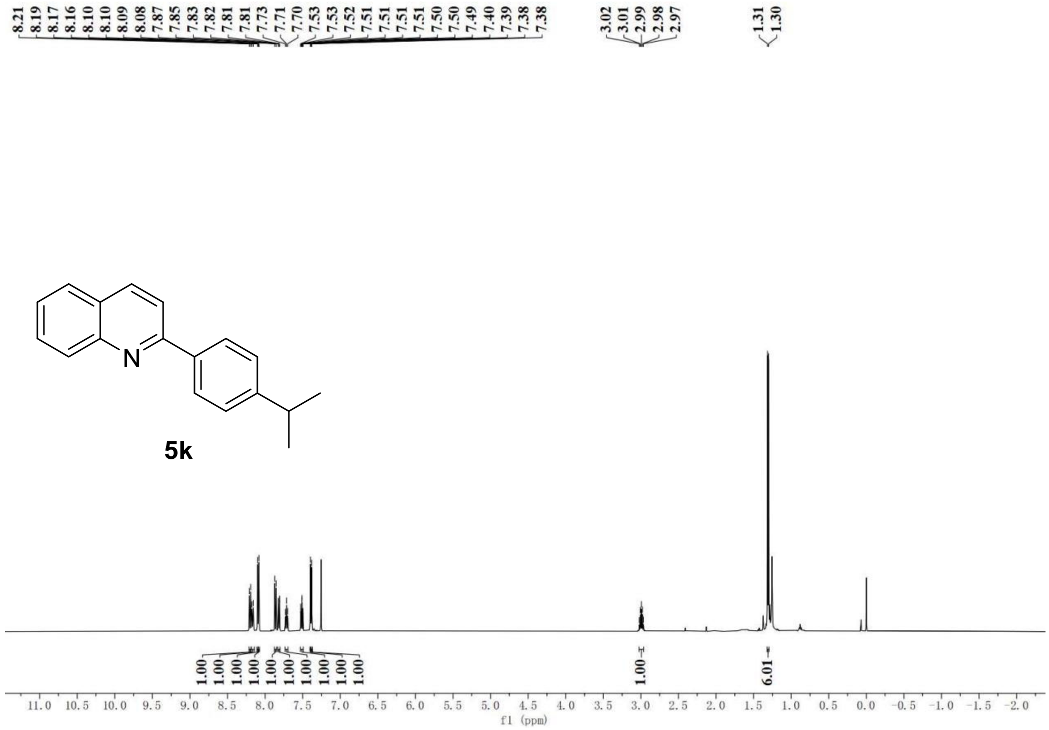
8.21
8.19
8.17
8.16
8.10
8.10
8.09
8.08
7.87
7.85
7.83
7.82
7.81
7.81
7.73
7.71
7.70
7.53
7.53
7.52
7.51
7.51
7.51
7.51
7.50
7.50
7.49
7.40
7.39
7.38
7.38
3.02
3.01
2.99
2.98
2.97
1.31
1.30
N
5k
1.00
1.00
1.00
1.00
1.00
1.00
1.00
1.00
1.00
1.00
1.00
6.01
11.0 10.5 10.0 9.5 9.0 8.5 8.0 7.5 7.0 6.5 6.0 5.5 5.0 4.5 4.0 3.5 3.0 2.5 2.0 1.5 1.0 0.5 0.0 -0.5 -1.0 -1.5 -2.0
f1 (ppm)

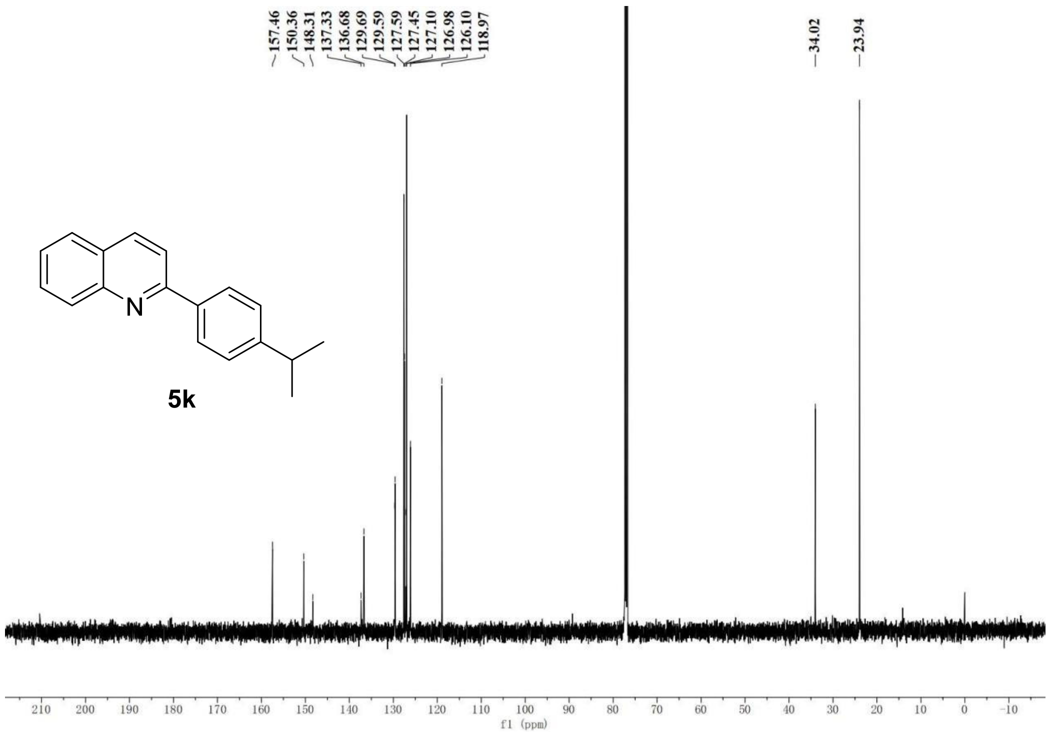
157.46
150.36
148.31
137.33
136.68
129.69
129.59
127.59
127.45
127.10
126.98
126.10
118.97
34.02
23.94
f1 (ppm)

5k

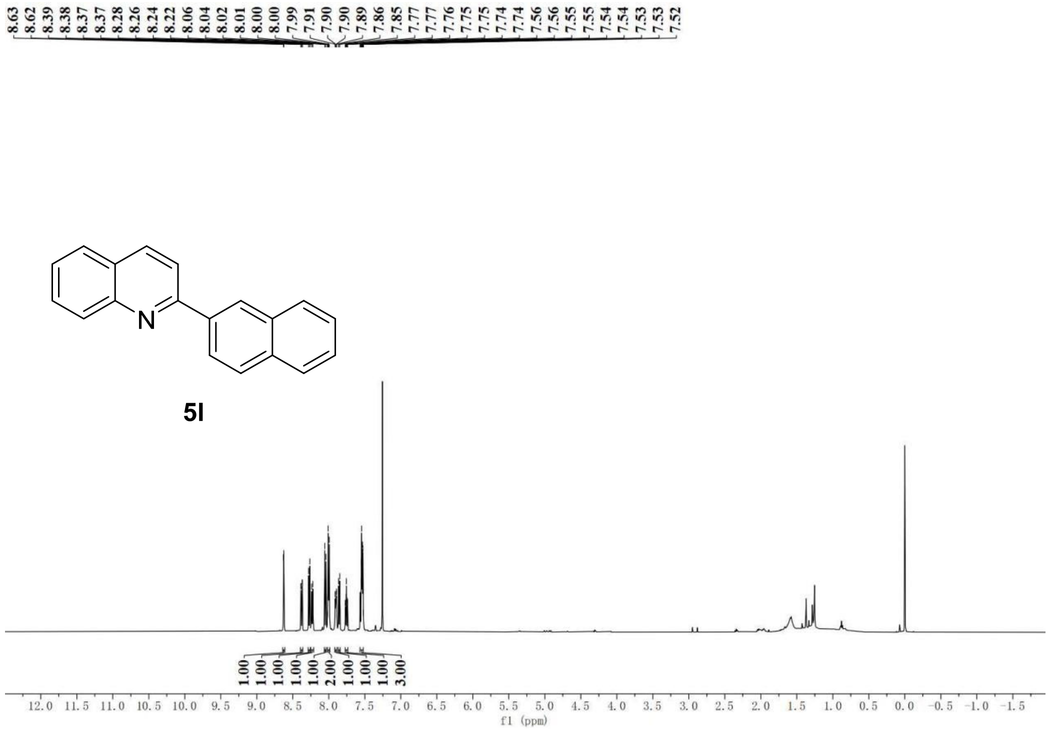
8.63
8.62
8.39
8.38
8.37
8.37
8.28
8.26
8.24
8.22
8.06
8.04
8.02
8.01
8.00
8.00
7.99
7.91
7.90
7.90
7.89
7.86
7.85
7.77
7.77
7.76
7.75
7.75
7.74
7.74
7.56
7.56
7.55
7.55
7.54
7.54
7.53
7.53
7.52
1.00
1.00
1.00
1.00
1.00
2.00
1.00
1.00
1.00
3.00
f1 (ppm)

5l

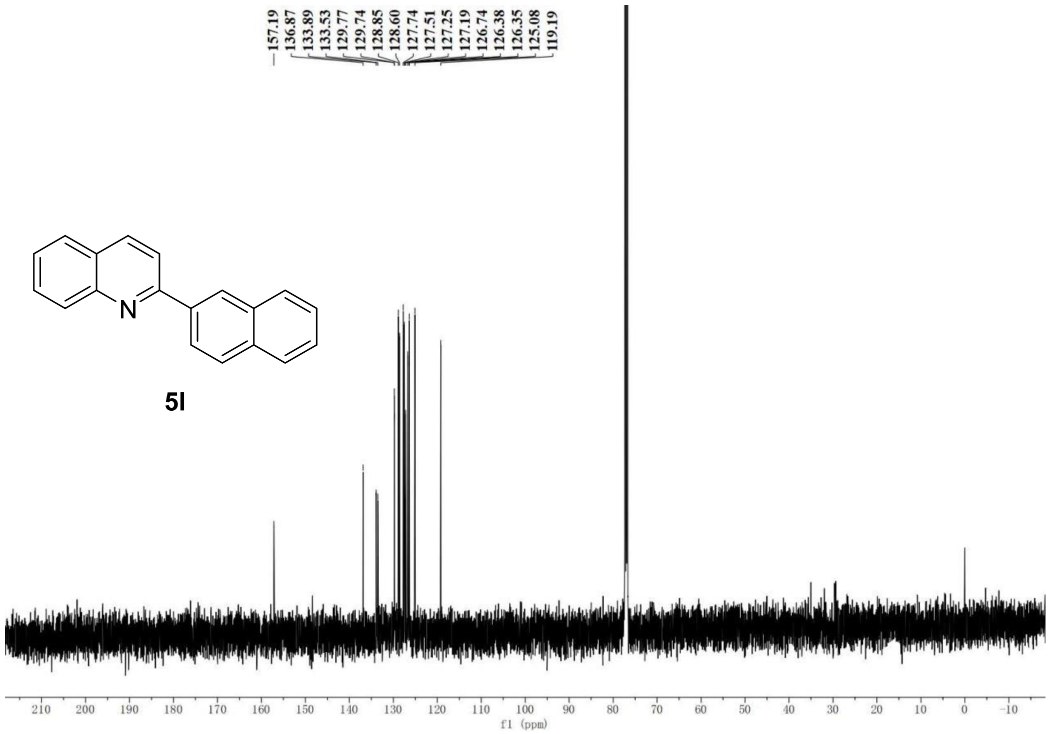
157.19
136.87
133.89
133.53
129.77
129.74
128.85
128.60
127.74
127.51
127.25
127.19
126.74
126.38
126.35
125.08
119.19
N
5l
f1 (ppm)

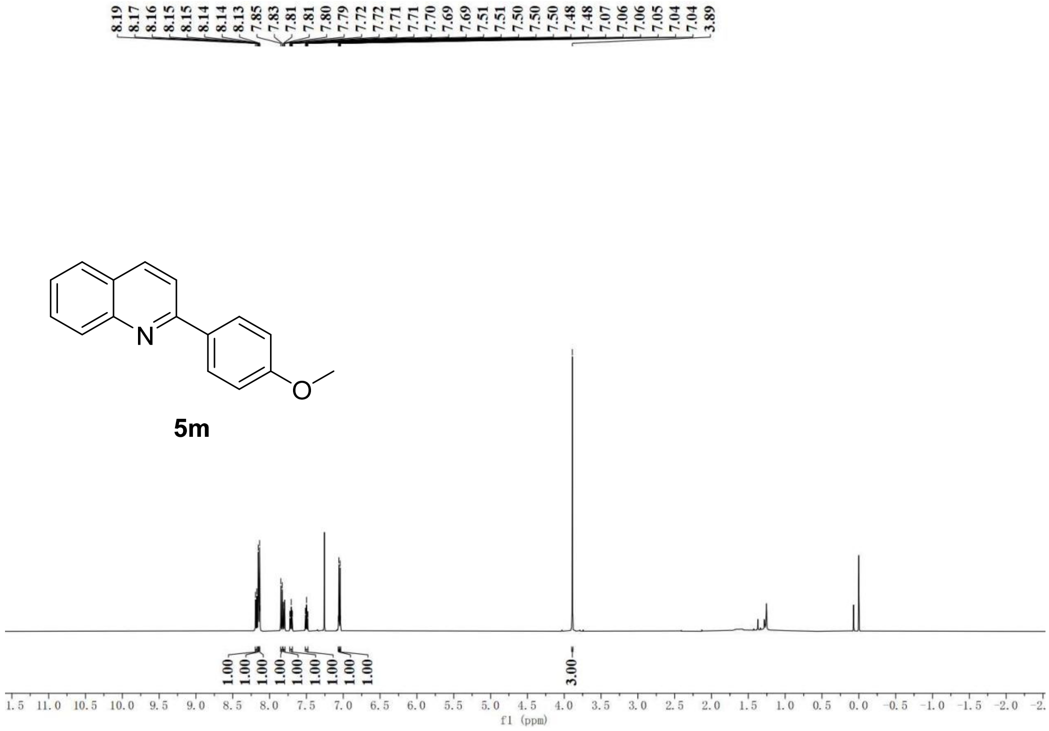
8.19
8.17
8.16
8.15
8.15
8.14
8.14
8.13
7.85
7.83
7.81
7.81
7.80
7.79
7.72
7.72
7.71
7.71
7.70
7.69
7.69
7.51
7.51
7.50
7.50
7.50
7.48
7.07
7.06
7.06
7.05
7.04
7.04
3.89
N
O
5m
1.00
1.00
1.00
1.00
1.00
1.00
1.00
1.00
3.00
f1 (ppm)

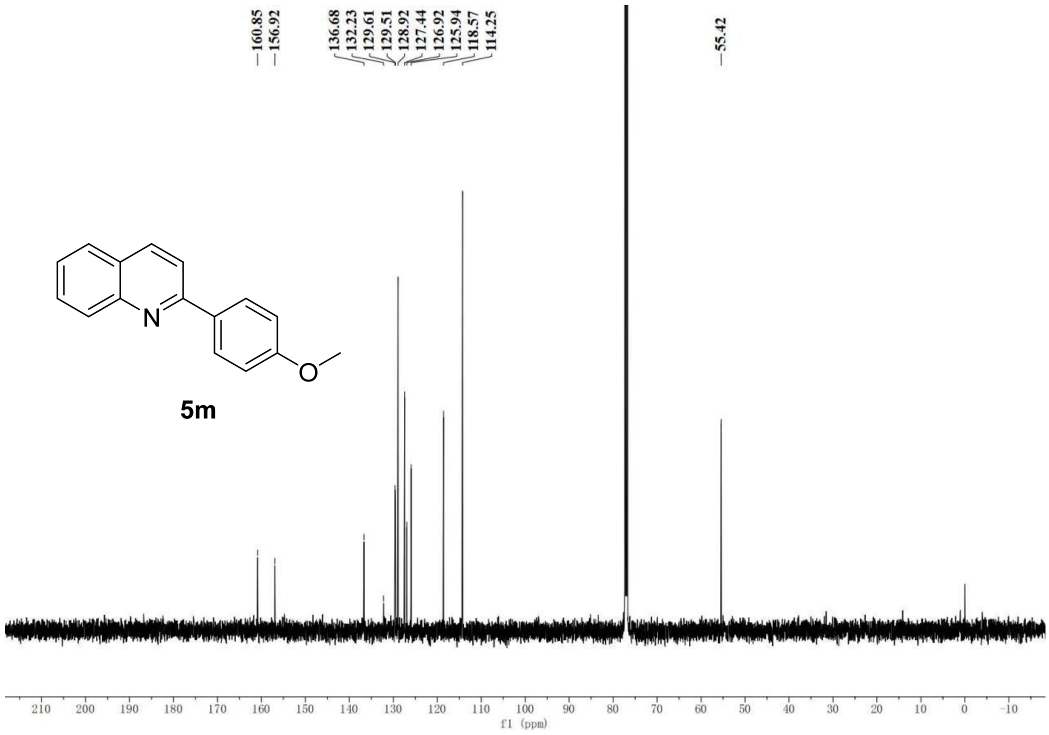

160.85
156.92
136.68
132.23
129.61
129.51
128.92
127.44
126.92
125.94
118.57
114.25
55.42
N
O
5m
210 200 190 180 170 160 150 140 130 120 110 100 90 80 70 60 50 40 30 20 10 0 -10
f1 (ppm)

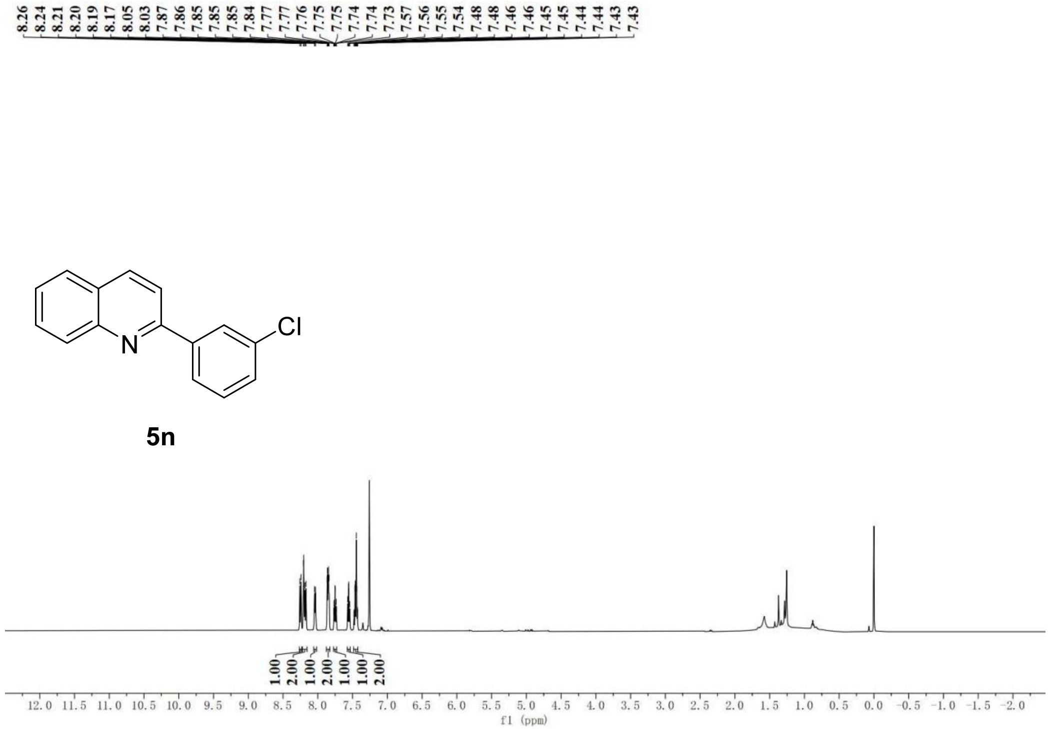

8.26 8.24 8.21 8.20 8.19 8.17 8.05 8.03 7.87 7.86 7.85 7.85 7.85 7.84 7.77 7.77 7.76 7.75 7.75 7.74 7.74 7.73 7.57 7.56 7.55 7.54 7.48 7.48 7.46 7.46 7.45 7.45 7.44 7.44 7.43 7.43
N
Cl
5n
1.00 2.00 1.00 2.00 1.00 1.00 2.00
12.0 11.5 11.0 10.5 10.0 9.5 9.0 8.5 8.0 7.5 7.0 6.5 6.0 5.5 5.0 4.5 4.0 3.5 3.0 2.5 2.0 1.5 1.0 0.5 0.0 -0.5 -1.0 -1.5 -2.0
f1 (ppm)

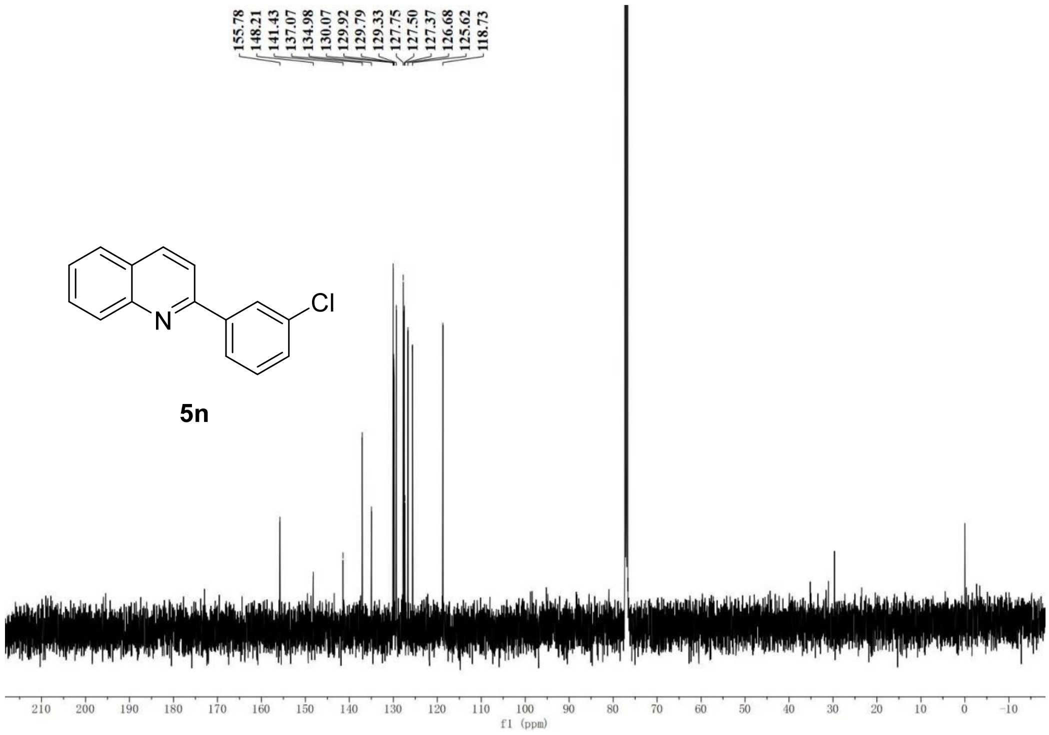
155.78
148.21
141.43
137.07
134.98
130.07
129.92
129.79
129.33
127.75
127.50
127.37
126.68
125.62
118.73
Cl
N
f1 (ppm)

5n

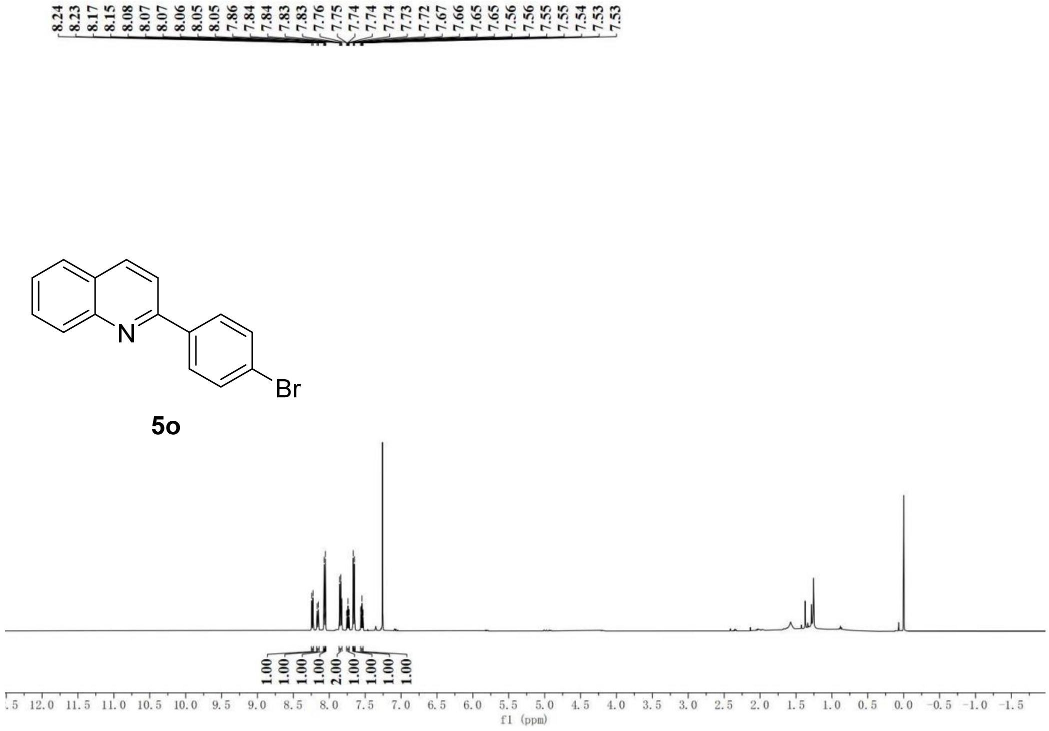
8.24
8.23
8.17
8.15
8.08
8.07
8.06
8.05
7.86
7.84
7.84
7.83
7.83
7.76
7.75
7.74
7.74
7.74
7.73
7.72
7.67
7.66
7.65
7.65
7.56
7.56
7.55
7.55
7.54
7.53
7.53
N
Br
1.00
1.00
1.00
2.00
1.00
1.00
1.00
f1 (ppm)

5o

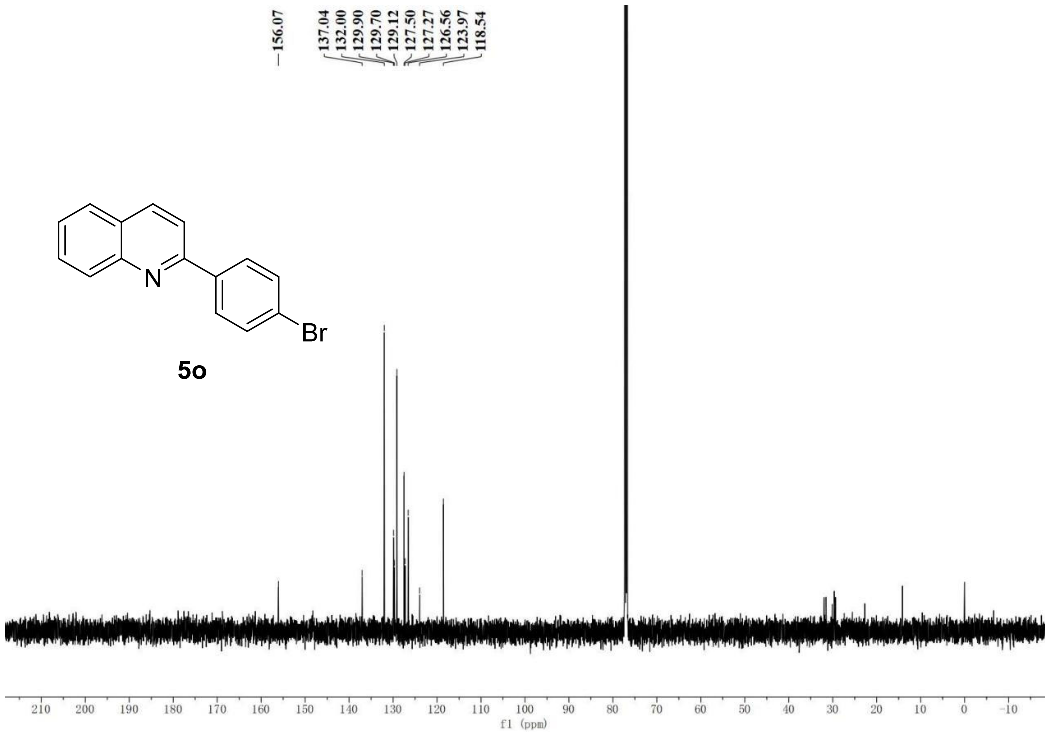
156.07
137.04
132.00
129.90
129.70
129.12
127.50
127.27
126.56
123.97
118.54
N
Br
5o
210 200 190 180 170 160 150 140 130 120 110 100 90 80 70 60 50 40 30 20 10 0 -10
f1 (ppm)
